# Novel biomarkers and prediction model for the pathological complete response to neoadjuvant treatment of triple-negative breast cancer

**DOI:** 10.7150/jca.52439

**Published:** 2021-01-01

**Authors:** Yiqun Han, Jiayu Wang, Binghe Xu

**Affiliations:** Department of Medical Oncology, National Cancer Center/National Clinical Research Center for Cancer/Cancer Hospital, Chinese Academy of Medical Sciences and Peking Union Medical College. No. 17, Panjiayuan Nanli, Chaoyang District, Beijing 100021, China.

**Keywords:** triple-negative breast cancer, neoadjuvant chemotherapy, pathological complete response, nomogram, molecular heterogeneity

## Abstract

**Objective:** To develop and validate a prediction model for the pathological complete response (pCR) to neoadjuvant chemotherapy (NCT) of triple-negative breast cancer (TNBC).

**Methods:** We systematically searched Gene Expression Omnibus, ArrayExpress, and PubMed for the gene expression profiles of operable TNBC accessible to NCT. Molecular heterogeneity was detected with hierarchical clustering method, and the biological profiles of differentially expressed genes were investigated by Gene Ontology, Kyoto Encyclopedia of Genes and Genomes analyses, and Gene Set Enrichment Analysis (GSEA). Next, machine-learning algorithms including random-forest analysis and least absolute shrinkage and selection operator (LASSO) analysis were synchronously performed and, then, the intersected proportion of significant genes was undergone binary logistic regression to fulfill variables selection. The predictive response score (pRS) system was built as the product of the gene expression and coefficient obtained from the logistic analysis. Last, the cohorts were randomly divided in a 7:3 ratio into training cohort and validation cohort for the introduction of a robust model, and a nomogram was constructed with the independent predictors for pCR rate.

**Results:** A total of 217 individuals from four cohort datasets (GSE32646, GSE25065, GSE25055, GSE21974) with complete clinicopathological information were included. Based on the microarray data, a six-gene panel (ATP4B, FBXO22, FCN2, RRP8, SMERK2, TET3) was identified. A robust nomogram, adopting pRS and clinical tumor size stage, was established and the performance was successively validated by calibration curves and receiver operating characteristic curves with the area under curve 0.704 and 0.756, respectively. Results of GSEA revealed that the biological processes including apoptosis, hypoxia, mTORC1 signaling and myogenesis, and oncogenic features of EGFR and RAF were in proactivity to attribute to an inferior response.

**Conclusions:** This study provided a robust prediction model for pCR rate and revealed potential mechanisms of distinct response to NCT in TNBC, which were promising and warranted to further validate in the perspective.

## Introduction

Triple-negative breast cancer (TNBC) is a special molecular subtype of breast cancer, of which accounts for around 15% proportion, and marked by the absent expression of estrogen receptor (ER), progesterone receptor (PR), and human epidermal growth factor receptor 2 (HER2) 1. Biological profiles of TNBC tend to be aggressive, which is characterized by the early relapse and distant metastases in addition to the inferior prognosis with 5-year survival rate of less than 30% 2. Systemic treatment has been taken into account as the mainstay, which cytotoxic agents are widely applied in the overall course of therapeutics for TNBC. Considering the acknowledged aggressiveness, the neoadjuvant chemotherapy (NCT) has been recommended for TNBC within a broader range, in comparisons with the other subtypes, and pathological complete response (pCR) rate was used to assess the efficacy in associations with a better prognosis of TNBC undergone NCT 3.

Although systemic treatment is the essential composition of the therapeutic introduction for TNBC, the pCR rate is just 35-40% of the whole group of patients based on the application of standard-of-care protocols. Besides, several efforts have been put in the field of pCR prediction including both clinical and experimental management. However, except for a few predictors such as BRCA1 deficiency for TNBC treated with platinum-containing NCT, no efficient and robust biomarkers have been yet generally recommended 4-9. This dilemma could be the result of molecular heterogeneities of TNBC. Previous studies have managed to explore the heterogenous subtypes of TNBC and propose different classifications with distinct biological profiles, which some specific subtypes tend to chemosensitive to neoadjuvant therapy 10, 11. Additionally, current findings remain still in the translational or even experimental phases, indicating the fulfillment of general application in clinical practice is challenging.

Recently, dissection of multi-omics data through bioinformatic tools has been used to provide precise insight on the cancer biology and novel parameters for cancer therapy 12, 13. Howbeit, it is crucial to systematically integrate the molecular data and clinicopathological characteristics for the broad application. Herein, we carried out this study to construct and validate a prediction model for the pCR rate of neoadjuvant chemotherapy for TNBC patients, with the aim of the accumulation of solid evidence for clinical practice and the potential survival benefit.

## Materials and Methods

### Datasets selection and preprocessing

Gene expression profiles of operable TNBC accessible to NCT were systematically searched on Gene Expression Omnibus (GEO), ArrayExpress, and PubMed. Cohorts datasets were eligible if the met the inclusion criteria: (1) operable TNBC patients with complete clinicopathological information were adopted; (2) definite clinical efficacy of neoadjuvant treatment were recorded. Raw gene expression matrix and supplementary materials were obtained and data from TNBC patients were identified through subtype selection. The multiple gene panel corresponding to a single probe was divided into individuals, and only the maximum expression values of genes were preserved for the following analysis. Each cohort dataset was independently processed and further examined for batch effect with the removal using R package limma (version 3.42.2) 14.

### Exploration of subtypes and biologic profiles

To determine the optimal numbers of TNBC subtypes with the potentially distinct response to therapeutics, the consensus clustering method using R package ConsensusClusterPlus (version 1.50.0) was performed using 1000 iterations of hierarchical clustering based on Pearson correlation to assess the relative closeness of each clusters. The differential expressed genes (DEGs) were retrieved using R package limma, which the absolute of fold change (FC) more than 1 and P value adjusted by Benjamini-Hochberg method less than 0.05 were considered as the criteria for significant DEGs. Biological profiles of both up-regulated and down-regulated genes were respectively elucidated through enrichment analyses on the basis of Gene Ontology (GO) including biological processes, cellular components, and molecular functions, and the Kyoto Encyclopedia of Genes and Genomes (KEGG) comprising significantly enriched signaling pathways, by virtue of R package clusterProfiler (version 3.14.3), where the identified GO terms and pathways with FDR less than 0.05 were regarded as significant and presented with the leading proportions. The STRING database (http://string.db.org/) was used to clarify the interactive correlations among DEGs with protein-protein interaction (PPI) networks established and further visualized by Cytoscape software (version 3.8.0).

### Identification of predictive biomarkers

The retrieved DEGs were prepared for machine-learning algorithms to facilitate the dimensionally reduction and selection of features with the simultaneous performance of random-forest analysis and least absolute shrinkage and selection operator (LASSO) analysis using R package RandomForest (version 4.6-14) and glmnet (version 4.0-2) 15, which variable importance in random-forest analysis and the interpreting degree of variables in lasso regression analysis were used to successively assess the entire DEGs, respectively. Then, the significant genes identified from the couple methods were collected for the intersected proportions. Next, binary logistic regression analysis was carried out to discover the genes with the predictive values for pCR rate using R package survival (version 3.2-3). To quantitatively assess the promising values of biomarkers, the predictive response score (pRS) was defined as:


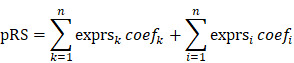


Where *n* is the number of genes. The exprs_k_ and coef_k_ was the gene expression and regression coefficient for DEGs in which the odds ratio (OR) more than 1, while exprs_i_ and coef_i_ was for the genes of which OR less than 1. The pRS of each patient was calculated and recorded for the following analysis.

### Construction and validation of prediction model for pCR rate

The included datasets were randomly in a 7:3 ratio divided into training cohort and validation cohort. Subsequently, the predictive model was constructed in training cohort with the combination of pRS and the clinicopathological factors through logistic regression analysis, and the characteristics with independent predictive values for pCR rate were adopted for nomogram using R package rms (version 6.0-0). Receiver operating characteristic (ROC) curves with the calculated area under the curve (AUC), using R package pROC (version 1.16.2) were utilized to validate the discriminative power of this model, while calibration plot was adopted for calibrating capability.

### Quest for promising clinical response

Eligible patients were classified into high pRS group and low pRS group with the borderline of the median value, which was followed by differential analysis to investigate the potential mechanisms of distinct response. After the DEGs were identified, the Gene Set Enrichment Analysis (GSEA) was performed with 1000 permutations referred to gene sets of h: hallmarks (h.all. v7.1. symbols) and c6: oncogenic signatures (c6.all.v7.1. symbols) downloaded from Molecular Signatures Database (MSigDB, https://gsea-msigdb.org/) using R package ClusterProfiler (version 3.14.3).

### Statistical analysis

In this study, statistical tests were two-sided and P value less than 0.05 was considered as significant. All the statistical analyses were accomplished by SPSS (version 26.0) and R software (version 3.6.4).

## Results

A total of eighteen cohort datasets were initially retrieved, and 217 TNBC patients from four GEO datasets (GSE32646, GSE25065, GSE25055, GSE21974) were eligible with 193 patients finally adopted. The median age of selected cohort was 49 years. The procedure of population selection and flow diagram was presented in Figure S1, and the detailed information was listed in Table S1, Table S2, and Table S3.

### Three phenotypes and differential signatures of TNBC

**Figure 1:**
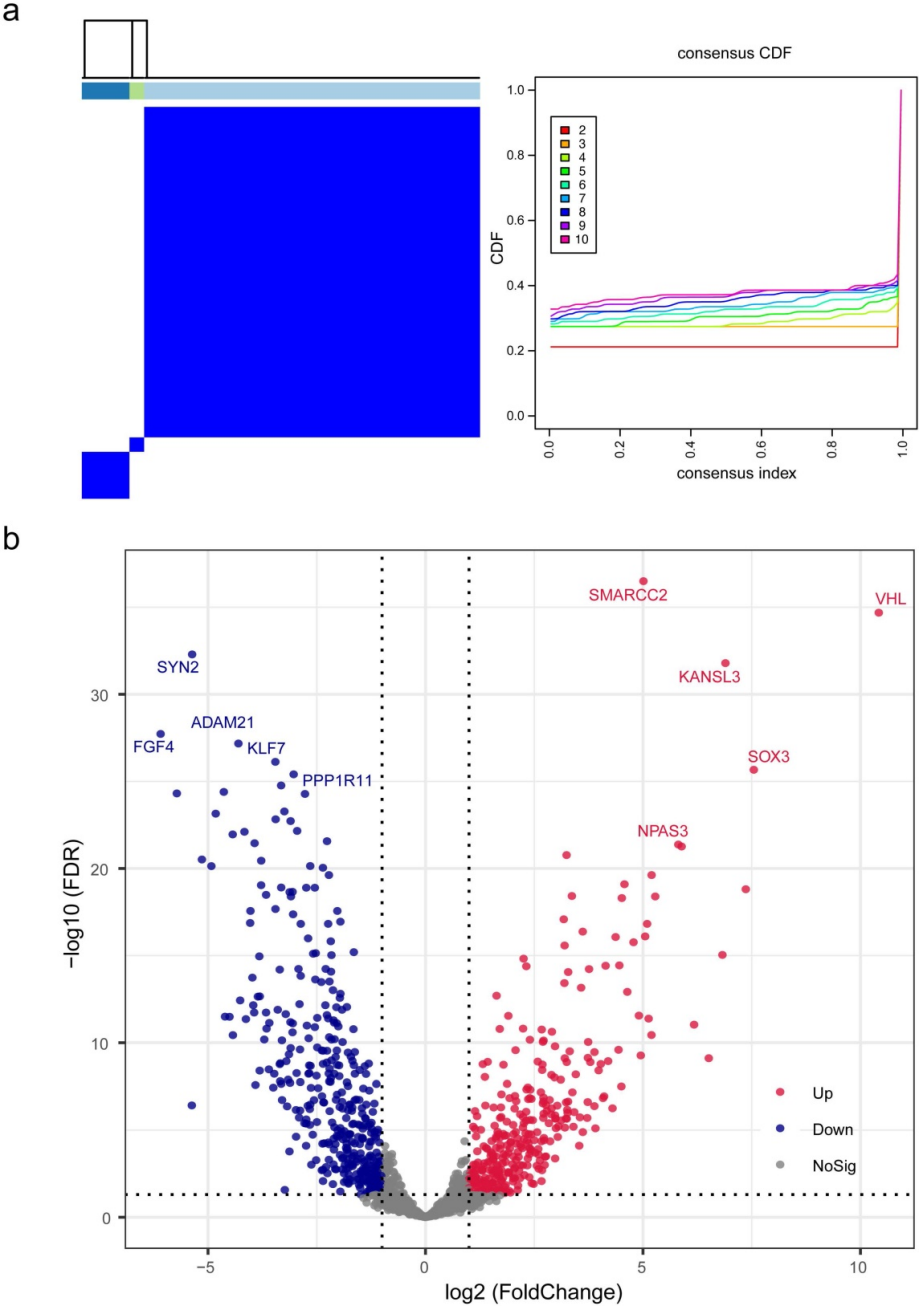
** Identification and differential analysis of triple-negative breast cancer subtypes.** (a) Three stable phenotypes of TNBC subject to neoadjuvant chemotherapy were identified through consensus clustering algorithms based on hierarchical clustering method. (b) A total of 1487 differentially expressed genes were recognized by differential analysis, which included 326 up-regulated genes and 381 down-regulated genes with statistical significance. Abbreviations: CDF: cumulative distribution functions.

On the basis of gene expression profiles, three stable phenotypes of TNBC subject to neoadjuvant chemotherapy were identified (Figure 1a), with the assumption that the response to therapeutics and clinical evolvements was intrinsically heterogenous. Differential analysis was performed to clarify potential mechanisms attributed from DEGs, and there were 1487 DEGs identified including 326 up-regulated genes and 381 down-regulated genes with statistical significance (Figure 1b; Table S4). Among this proportion of DEGs, SMARCC2, VHL, KANSL3, SOX3, and NPAS3 were the utmost significant up-regulated genes, while SYN2, FGF4, ADAM21, KLF7, and PPP1R11 were the foremost down-regulated genes.

**Figure 2:**
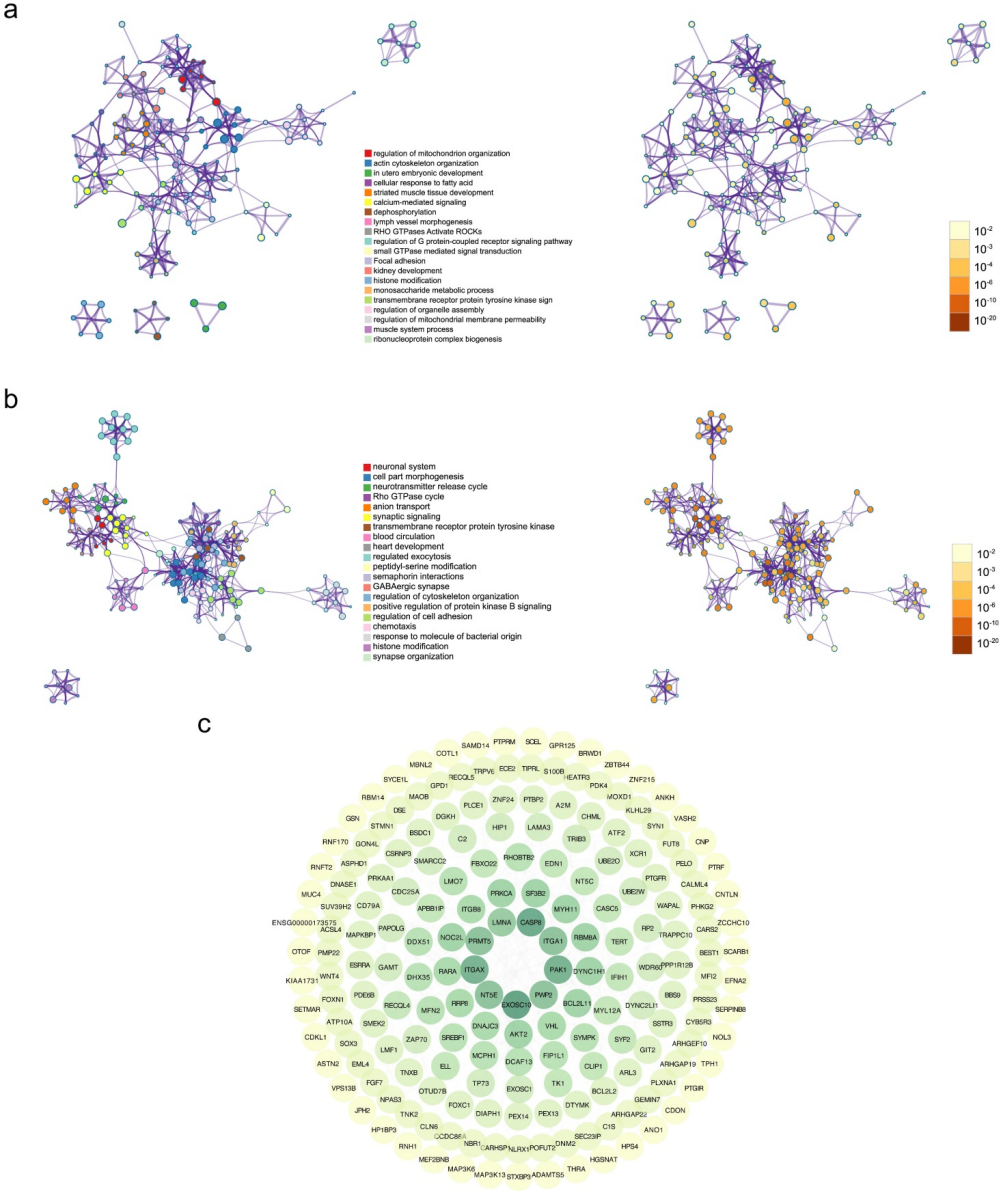
** Gene Ontology (GO) analysis and Kyoto Encyclopedia of Genes and Genomes (KEGG) analysis of differentially expressed genes.** (a) The enrichment results of GO and KEGG analyses for up-regulated genes, with the mapped items and the significance were presented respectively. (b) The enrichment results of GO and KEGG analyses for down-regulated genes, with the mapped items and the significance were presented respectively. In (a) and (b), the cycle size represented the relative enriched count, and the color intensity indicated the enriched significance. (c) Protein-protein network of identified differentially expressed genes.

To illustrate biologic profiles of the DEGs, the significant up-regulated genes and down-regulated genes were undergone enrichment analyses, respectively. Results from enrichment analyses demonstrated that the up-regulated DEGs were remarkably mapped to the biological terms including regulation of mitochondrion organization, action cytoskeleton organization, and utero embryonic development, and the down regulated DEGs were substantially enriched in neuronal system, cell part morphogenesis, and neurotransmitter release cycle (Figure 2a-b). Interactive correlations among functional products of gene expression were measured in degree, and visualized in circular output as protein-protein interactive networks (Figure 2c). It was evident that the core functional expression lay in the panels comprising CASP8, ITGA1, ITGAX, PAK1, and EXOSC10.

### Predictive biomarkers and score system

**Figure 3:**
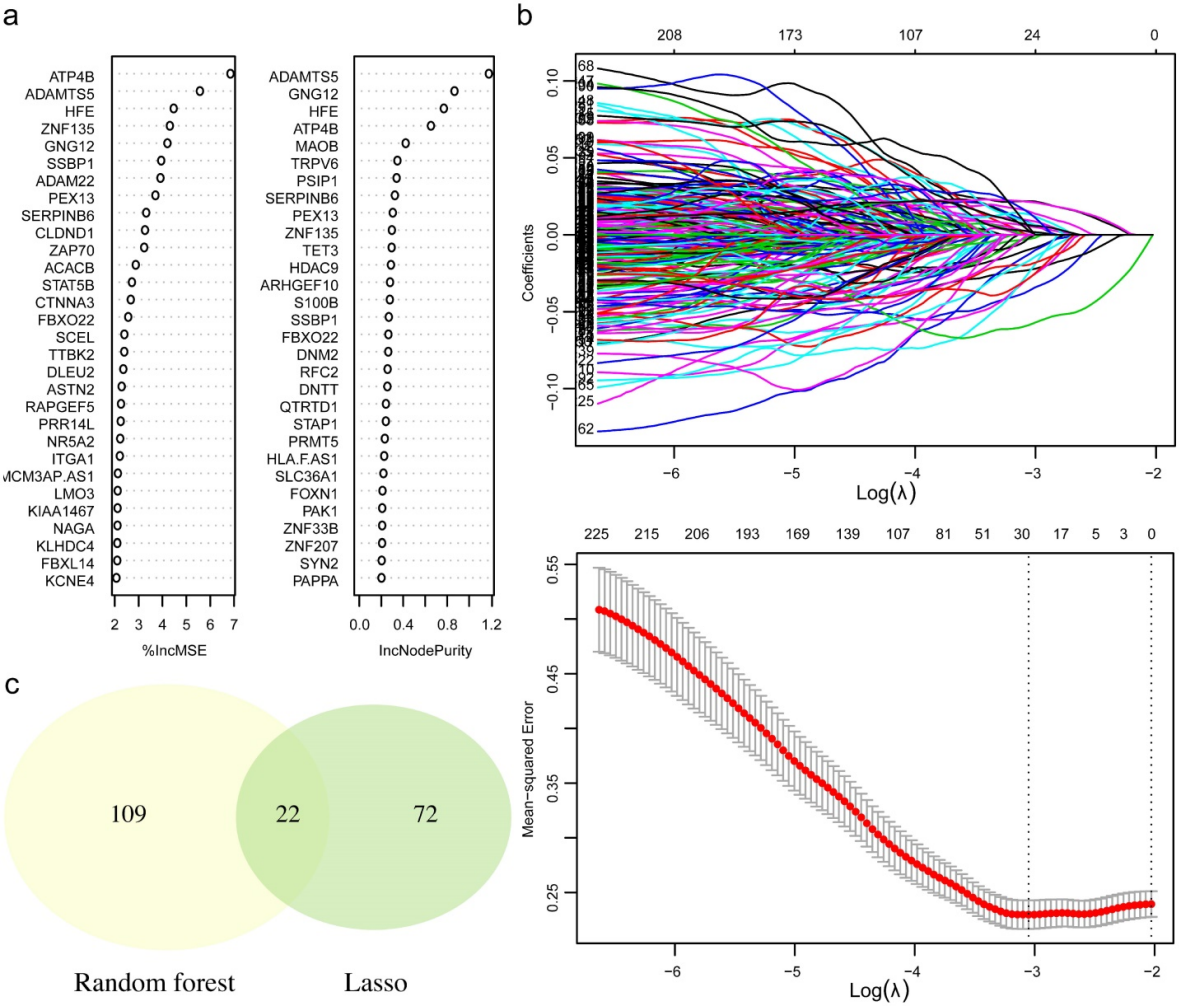
** The process of variables selection through machine-learning algorithms.** (a) The top 30 significant genes recognized from random forest analysis. (b) The performance and validation of least absolute shrinkage and selection operator (LASSO) analysis. (c) The intersected genes of these two analyses were selected. Abbreviations: IncMSE: increase in mean square error, IncNodePurity: increase in node purity.

**Figure 4:**
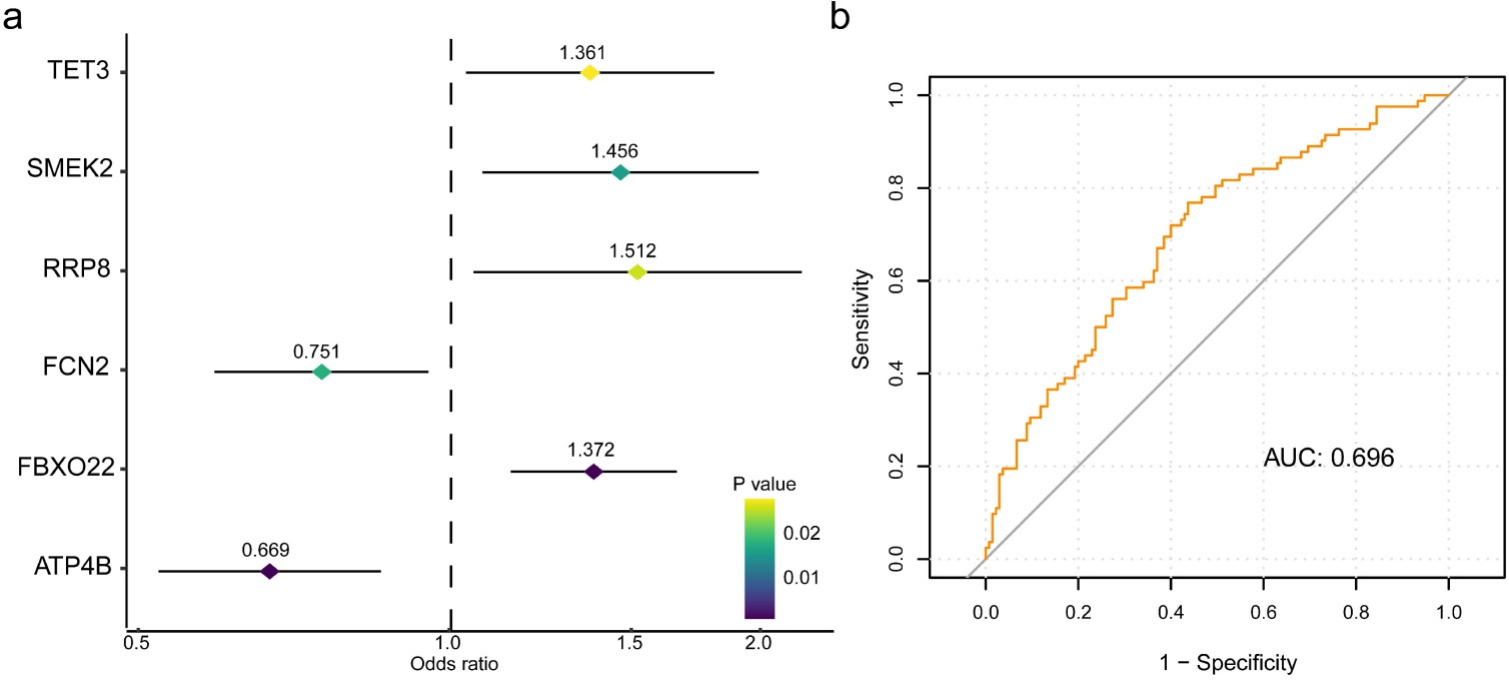
** The six-gene panel and validation of predictive response score (pRS) system.** (a) The six-gene panel including ATP4B, FBXO22, FCN2, RRP8, SMERK2, TET3 were identified through binary logistic analysis. (b) Receiver operating characteristic curve was built with the area under curve of 0.696.

To retrieve the most significant variables with predictive values, we dimensionally reduced the volume and selected the foremost characteristics based on machine-learning algorithms, which random forest analysis and Lasso regression analysis were concurrently carried out. There were 131 genes and 94 genes were respectively chosen, and 22 genes in total intersected which were considered to carry the foremost predictive power for pCR rate (Figure 3a-c). To further achieve the shrinkage of variables, the binary logistic regression analysis was performed toward each gene, of which the results revealed a total of 6 genes were statistically significant (Figure 4a; Table S5). Next, the pRS of each patient was determined as the product of gene expression and the corresponding coefficient obtained from the logistic analysis. ROC curve was used to depict the predictive power of this score system and the computational AUC was 0.696 (Figure 4b).

### Prediction model for pCR rate

Populations were randomly divided in a 7:3 ratio to training cohort and validation cohort to establish and validate the model. The integrity of characteristics consisted of clinicopathological factors, including age at diagnosis, clinical stage, histopathological grade, tumor size, nodal status, and pRS was adopted to construct the predictive model for pCR rate of NCT. Through binary logistic regression analysis, the significant variables were identified, which pRS (*P*<0.0001) and tumor size (*P*=0.024) were in statistical significance to this predictive value (Table S6). Then, nomogram was established and validated through the ROC curve for the discrimination power and calibration curve for the calibrating capability, respectively. The AUC of ROC curves from training cohort was 0.704 and from validation cohort was 0.756, while the calibration curve presented a slope at around 45 angles. Collectively, this predictive model for the pCR rate was well-performed.

### Potential mechanisms of distinct response

To further investigate the potential mechanisms of distinct response to neoadjuvant therapy in TNBC, differential analysis was performed between high pRS group and low pRS group, and followed by enrichment analysis based on the rendered gene sets. A total of 98 genes were considered as statistically significant, which GIT2, DYNC1H1, FN1, CNPY2, PTPN11 were evidently up-regulated, and AFF4, FGD2, MYO16, GLS2, CCDC132 were of the foremost down-regulated proportions (Table S7). Results of GSEA revealed that the biological processes associated with apoptosis, hypoxia, mTORC1 signaling, and myogenesis were up regulated, while the oncogenic signatures including EGFR and RAF were up regulated and CTIP and RELA were down regulated.

## Discussion

Overall, this study identified a prediction score system curated from a six-gene panel, and constructed a predictive model adopting clinicopathological characteristics for pCR rate of NCT in TNBC. Given the distinct response to systemic therapy, potential mechanisms were investigated and the promising signatures were identified.

Indeed, the heterogeneity of molecular features and the optimal numbers of TNBC subtypes have been long explored, and it is well recognized that this kind of divergence could result in distinct clinical profiles and survival outcomes 10, 11, 16. With the consideration of molecular heterogeneities, we identified three distinct subtypes of TNBC with the receipt of NCT through consensus clustering method, and further investigated the genomic difference based on gene expression profiles. Previously, following the 7 subtypes of TNBC identified by Lehmann et al, Masuda and colleagues firstly confirmed the promising correlations between the cancer heterogeneity and response to NCT in TNBC, which was followed by increasing studies detecting this kind of association 10, 11. Our study consistently corroborated the molecular heterogeneity and acquired three stable classifications with potential divergent response to clinical therapeutics. Then, enrichment analyses were carried out to comprehensively explore the cellular component, molecular functions, and biological process. Both the up-regulated genes and down-regulated genes were undergone analyses, respectively, of which the results indicated that the changes in the identified dysregulated biological profiles could exert critical effects on the inherent heterogeneity and discordant response to NCT in TNBC. The core functional group of genes, including CASP8, ITGA1, ITGAX, PAK1, and EXOSC10, was presented in PPI network, suggestive of the significant roles in the biological course. Previous studies have been managed to discuss the biological profiles of this group of genes. As the critical factor in the course of extrinsic apoptosis, CASP8 was demonstrated to exhibit a potential relationship with breast cancer risk, clinicopathological features, and prognostic outcomes of breast cancer from prior cohort-based analyses 17-19. ITGAX, a component of integrin family, was proven to promote angiogenesis in cancer development and aggressiveness 20. The associations between PAK1 and breast cancer have been extensively investigated, of which the results indicated that this kind of potential therapeutic target participated in several progressive courses in malignancies 21-23. Although the association between this genetic panel and the specific molecular subtypes of breast cancer has not been fully elucidated in clinical cohorts 19, or even some findings were just lied in preclinical theories, the existing evidence suggested that this kind of relationship was compelling enough to be further explored.

To facilitate the introduction of a practical prediction model, we conducted analyses on the basis of random forest analysis and Lasso analysis to retrieve the intersected proportion for dimensionally reduction and features selection, which has been widely used for characteristics of cancer diagnosis and therapy 24-26. After this group of genes identified, the respective prediction values for pCR were successively evaluated by logistic analysis, and the six-gene panel comprising ATP4B, FBXO22, FCN2, RRP8, SMERK2, TET3 were finally recognized. With the application of mathematics formula, pRS system was quantitatively established with a decent value for pCR rate. To optimize this prediction method, the clinicopathological characteristics were integrated and assessed the predictive values to select the value multi-variables, which pRS and clinical stage of tumor size were ultimately determined.

With the selected gene panel and variables, the nomogram was constructed to predict the estimated pCR rate in TNBC 27. Results from validation analyses were suggestive of the well performance of this model and the rationale of broad application in clinical practice. Several studies have managed to identify predictive factors for pCR of NCT in TNBC which was estimated to perform well in clinical practice 28-30. However, most of them basically focused on the characteristics from imaging or laboratory indexes. From molecular perspective, Hamy et al focused on the predictive values of immunological infiltration for the response to NCT in TNBC, while Ocana and colleagues investigated the immunologic phenotypes of TNBC and the potential associations with the clinical outcomes, which was inconsistent with the findings curated from the study conducted by Fournier et al. 31-33. Although the heterogenous profiles of TNBC have been extensively discussed, these predictive techniques were primarily centered on the molecular characteristics with few clinical features adopted. In the present study, we took full consideration of the molecular heterogeneities detected from transcriptome information and clinicopathological characteristics to provide practical prediction of pCR rate for TNBC patients planned to NCT. Besides, we also discussed the potential mechanisms of benefit and non-benefit phonotypes in order to provide promising evidence for practice. Biological processes including apoptosis, hypoxia, mTORC1 signaling and myogenesis, and oncogenic features of EGFR and RAF were in proactivity to attribute to an inferior response. In fact, the potential triggers of distinct response to NCT remain undetermined, considering these findings to some extent enlighten the quest for improvement of efficacy, which were in accordance with the previous studies 34-37. Current trials have exerted efforts and assess the efficacy of this kind of targeted therapies in TNBC 38, 39, while these results were controversial and remained to be updated through randomized controlled trials with a large sample cohort.

Indeed, there were some inevitable limitations of this study. Firstly, this was a retrospective analysis of the adoption of the identified datasets from publicly databases, and the heterogeneities among populations from different cohorts could not be removed. Secondly, a few characteristics, such as the Ki67 index and therapeutic protocols, could not be taken into considerations due to the lack of records in a publicly available database, which could potentially weaken the prediction power of this model. Thirdly, the cutoff value of pRS was determined as the median which was practical yet less precise, which was necessary to be validated and optimized. Last, both experimental and clinical research is supposed to conduct and validate these findings obtained this study.

## Conclusion

In conclusions, our study established a robust prediction model based on the transcriptome signatures and clinicopathological features, taking molecular heterogeneities into consideration, for the pCR rate, and discussed the potential mechanisms of distinct response to NCT in TNBC, which were promising for the exploration of novel therapeutics. This prediction model is warranted to be further applied and validated among large-scale cohorts in the upcoming future.

## Supplementary Material

Supplementary figures and tables.

## Authors' contributions

- Conception and design: Binghe Xu, Jiayu Wang, Yiqun Han;
- Development of methodology: Yiqun Han;
- Acquisition of data: Yiqun Han;
- Analysis and interpretation of data: Yiqun Han, Binghe Xu, Jiayu Wang;
- Writing of the manuscript: Yiqun Han;
- Review and revision of the manuscript: Binghe Xu, Jiayu Wang, Yiqun Han;
- Study supervision: Binghe Xu, Jiayu Wang.

## Availability of data and materials

The datasets and/or analysis results used during the current study are available from the corresponding author upon reasonable request.

## Figures and Tables

**Figure 5:**
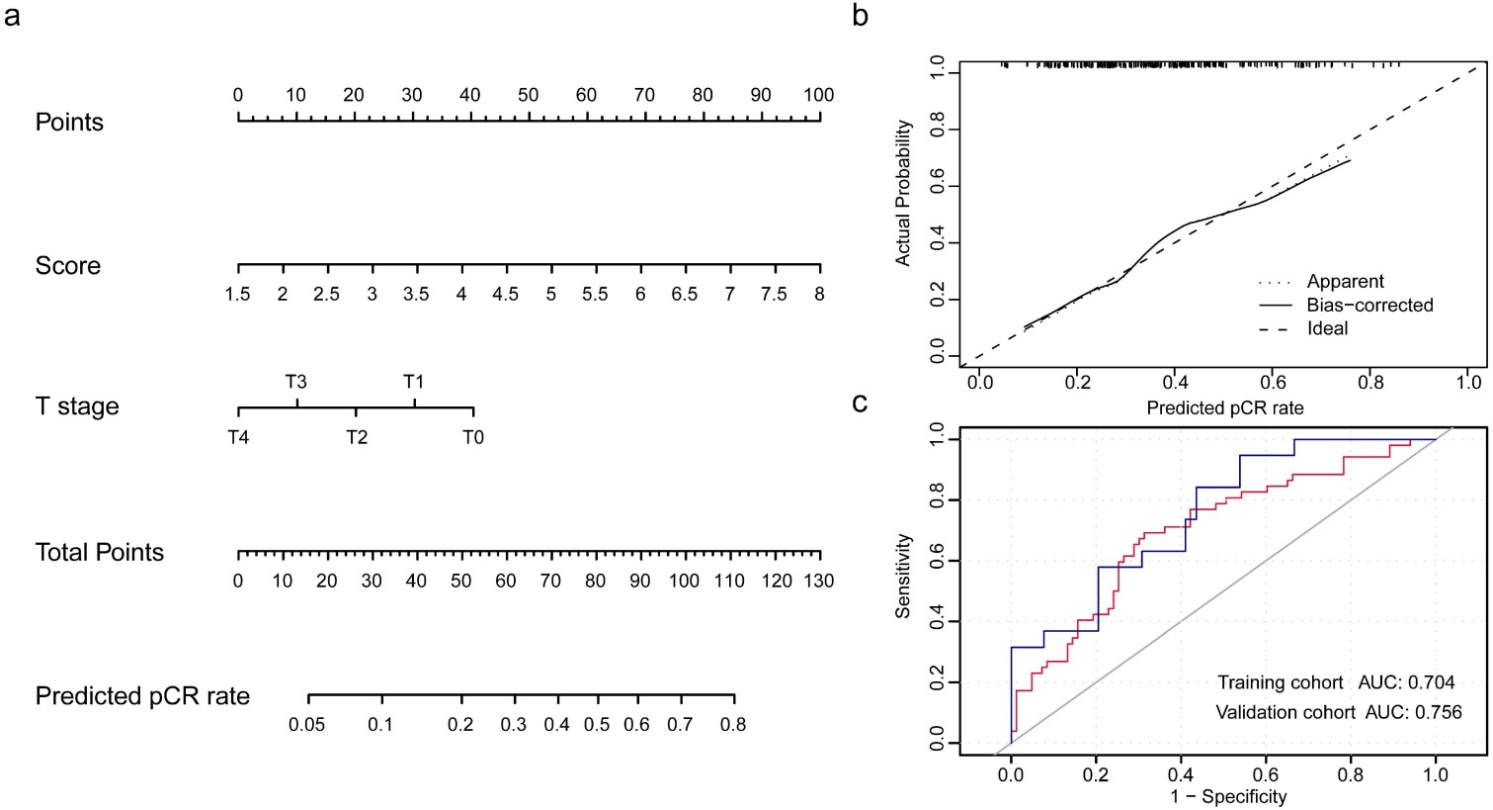
** Development and validation of nomogram for the prediction of pCR rate.** (a) The nomogram was established based on the independent predictors for the pCR rate in TNBC. (b) Calibration curve was presented with a slope at around 45 angles. (c) Receiver operating characteristic curves were built with the area under curve of 0.704 in training cohort and of 0.756 in validation cohort.

**Figure 6:**
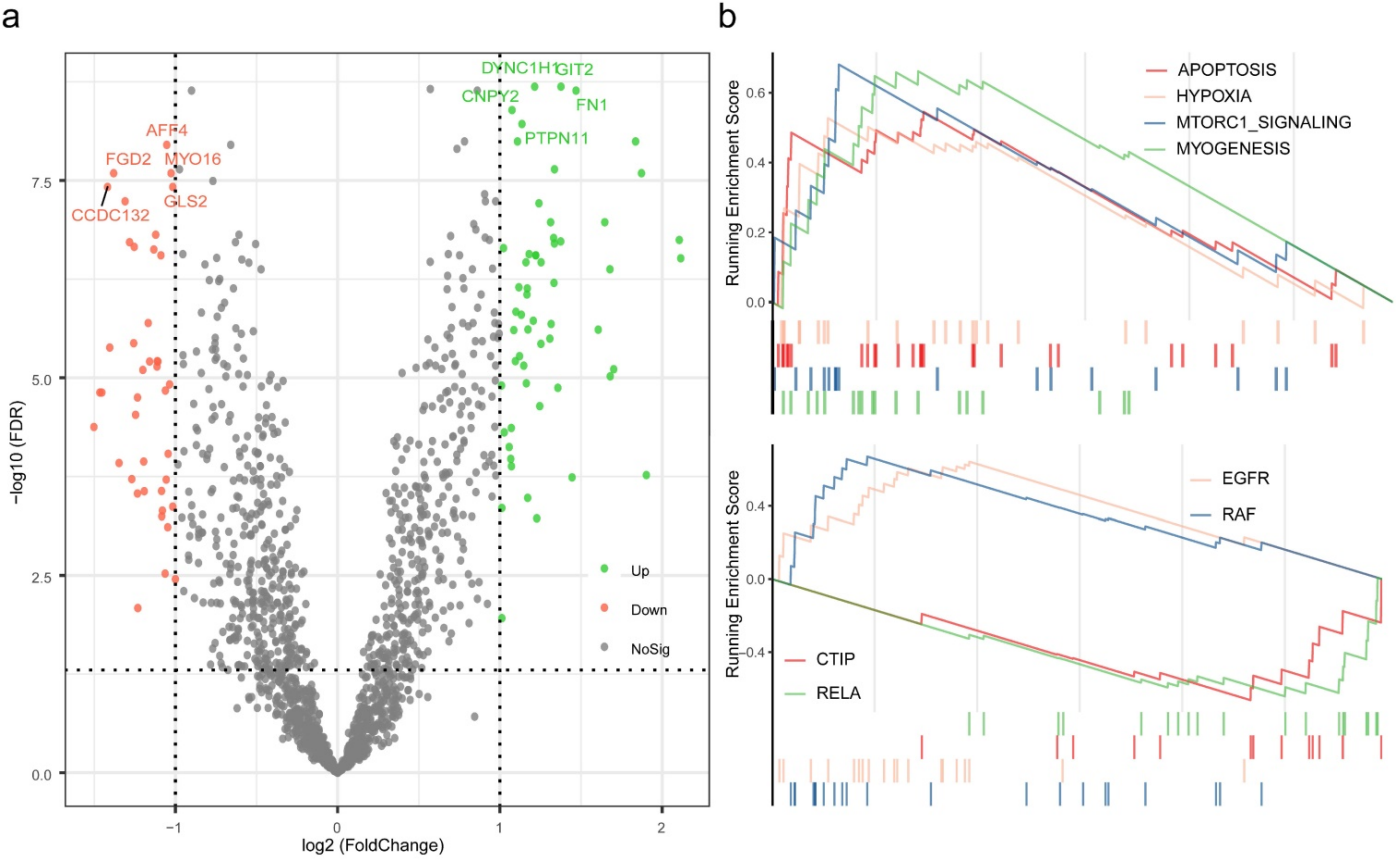
** Exploration of the mechanisms for distinct pCR rate.** (a) Differential analysis was performed between high pRS group and low pRS group. (b) Gene set Enrichment Analysis were conducted with the reference to gene lists of cancer hallmarks signatures and oncogenic signatures.

## Abbreviations

TNBC: triple-negative breast cancer
ER: estrogen receptor
PR: progesterone receptor
HER2: human epidermal growth factor receptor 2
NCT: neoadjuvant chemotherapy
pCR: pathological complete response
GEO: Gene Expression Omnibus
DEGs: differential expressed genes
FC: fold change
GO: Gene Ontology
KEGG: Kyoto Encyclopedia of Genes and Genomes
PPI: protein-protein interaction
LASSO: least absolute shrinkage and selection operator
pRS: the predictive response score
OR: odds ratio
ROC: receiver operating characteristic
AUC: area under curve
GSEA: Gene Set Enrichment Analysis
