# Supplementary material for: Novel biomarkers and prediction model for the pathological complete response to neoadjuvant treatment of triple-negative breast cancer
Source: J Cancer. 2021 Jan 1;12(3):936–45. doi: 10.7150/jca.52439 (PMC7778555; doi:10.7150/jca.52439)
Supplement: Supplementary file 1 — Supplementary figures and tables. [file jcav12p0936s1.pdf]

Supplementary materials

Figure S1. Flow diagram of selection and identification of cohort datasets.

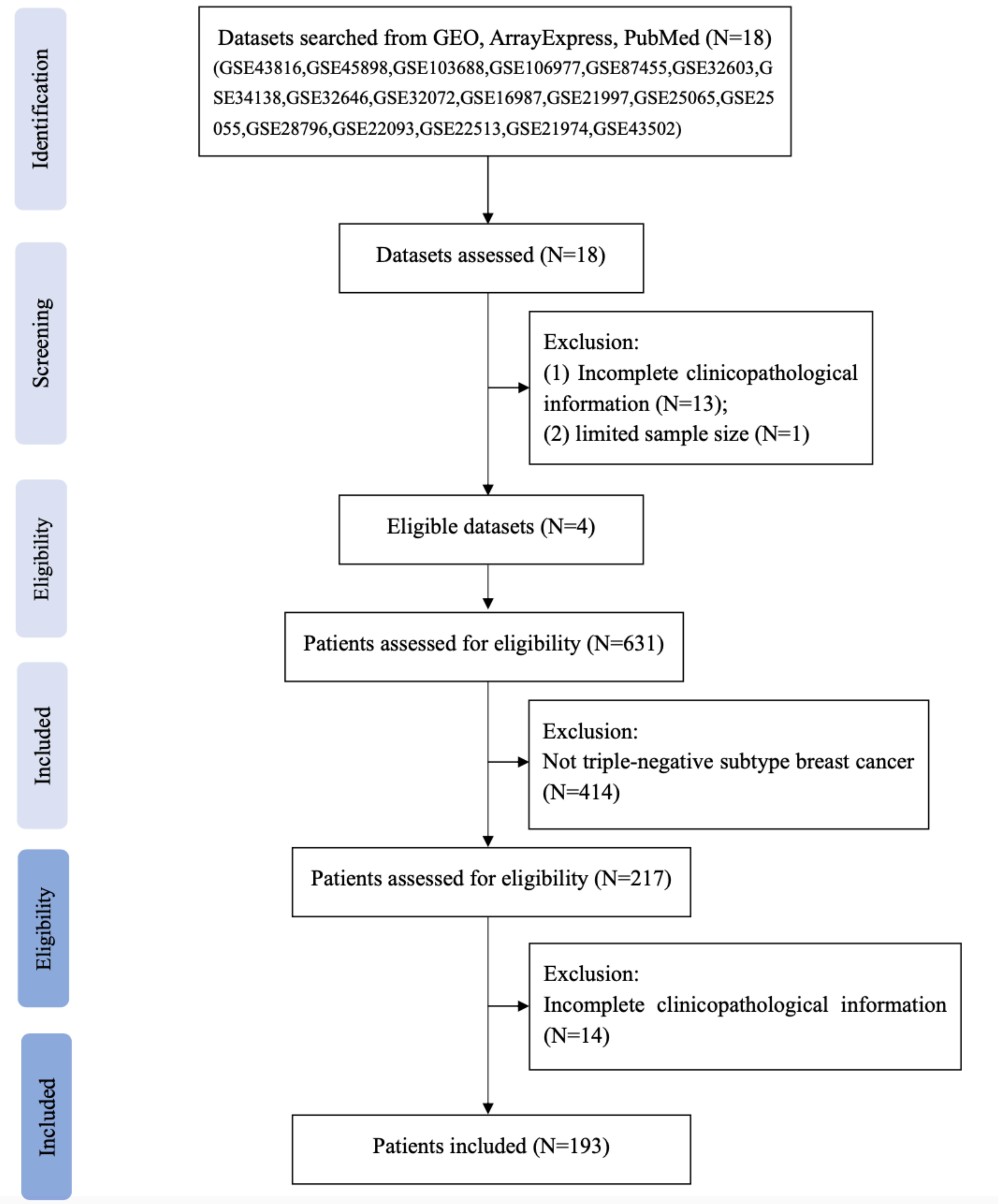

Table S1. Detailed information of eligible cohort datasets

Datasets	Platform	No. of BC	No. of TNBC
GSE32646	Affymetrix Human Genome U133 Plus 2.0 Array	115	26
GSE25065	Affymetrix Human Genome U133A Array	198	67
GSE25055	Affymetrix Human Genome U133A Array	310	120
GSE21974	Agilent-014850 Whole Human Genome Microarray 4x44K G4112F	32	8

Table S2. Summarized clinicopathological features of included patients

Age (median, range)	49.0 (24.0-75.0)
Clinical stage	
I	4 (2.1)
IIA	30 (15.5)
IIB	69 (35.8)
IIIA	52 (26.9)
IIIB	29 (15.0)
IIIC	9 (4.7)
T stage	
T1	10 (5.2)
T2	98 (50.8)
T3	60 (31.1)
T4	24 (12.4)
Nodal status	
Negative	46 (23.8)
Positive	147 (76.2)
Grade	
Grade1	1 (0.5)
Grade2	34 (7.6)
Grade3	158 (81.9)
Pathological response	
pCR	71 (36.8)
non-pCR	122 (63.2)

Table S3. Detailed information of eligible individuals from adopted cohorts.

Sample	Age	Stage	Grade	T stage	N stage	Response
GSM615098	41	IIB	3	3	0	non-pCR
GSM615101	52	IIIB	3	4	0	non-pCR
GSM615110	50	IIIA	3	3	1	pCR
GSM615116	54	IIIA	3	3	1	non-pCR
GSM615117	35	IIIA	3	3	1	non-pCR
GSM615124	61	IIIB	2	4	1	non-pCR
GSM615136	60	IIIA	3	3	1	non-pCR
GSM615140	39	IIIA	2	3	1	pCR
GSM615143	43	IIA	3	2	0	pCR
GSM615145	56	IIIA	3	3	1	non-pCR
GSM615146	39	IIA	3	2	0	pCR
GSM615148	40	IIIA	3	3	1	non-pCR
GSM615149	44	IIIA	3	3	1	non-pCR
GSM615151	45	IIA	2	2	0	non-pCR
GSM615152	44	IIIA	3	2	1	non-pCR
GSM615154	41	IIB	3	2	1	pCR
GSM615155	32	IIIB	3	4	1	non-pCR
GSM615156	38	IIB	3	2	1	non-pCR
GSM615160	38	IIIA	3	3	1	non-pCR
GSM615164	42	IIB	3	3	0	pCR
GSM615169	48	IIIA	3	3	1	non-pCR
GSM615175	43	IIB	3	3	0	pCR
GSM615178	39	IIB	3	2	1	non-pCR
GSM615181	49	IIIA	3	3	1	pCR
GSM615184	51	IIA	3	2	0	non-pCR
GSM615185	65	I	3	1	0	non-pCR
GSM615190	37	IIA	3	2	0	non-pCR
GSM615191	37	I	3	1	0	pCR
GSM615194	61	IIA	3	1	1	pCR
GSM615195	29	IIIB	3	4	1	non-pCR
GSM615202	42	IIB	3	2	1	pCR
GSM615209	56	IIIC	3	2	1	pCR
GSM615211	35	IIB	3	2	1	pCR
GSM615212	48	IIA	3	2	0	pCR
GSM615213	53	IIIA	3	3	1	pCR
GSM615218	56	IIIB	3	4	1	non-pCR
GSM615219	38	IIIB	3	4	1	non-pCR
GSM615223	57	IIIA	3	3	1	non-pCR
GSM615225	60	IIIB	3	4	1	non-pCR
GSM615240	48	IIB	3	2	1	pCR
GSM615242	46	IIIA	3	3	1	pCR
GSM615244	51	IIA	3	1	1	non-pCR
GSM615248	42	IIIA	3	3	1	non-pCR

GSM615251	44	IIB	3	2	1	non-pCR
GSM615255	39	IIIB	3	4	1	non-pCR
GSM615260	56	IIIB	3	0	1	non-pCR
GSM615264	45	IIA	3	2	0	pCR
GSM615270	38	IIIB	3	4	1	pCR
GSM615275	60	IIIB	2	4	1	non-pCR
GSM615276	52	IIIA	3	3	1	non-pCR
GSM615284	50	IIIB	3	4	1	pCR
GSM615288	38	I	3	1	0	non-pCR
GSM615290	66	IIA	2	2	0	non-pCR
GSM615291	59	IIB	3	2	1	pCR
GSM615293	31	IIA	3	2	1	pCR
GSM615297	58	IIB	3	2	1	non-pCR
GSM615300	57	IIIB	3	2	1	pCR
GSM615301	62	IIIB	3	4	1	non-pCR
GSM615303	68	IIIB	3	4	1	non-pCR
GSM615307	32	IIB	3	2	1	pCR
GSM615310	64	IIIA	3	2	1	pCR
GSM615312	51	IIIB	2	2	1	non-pCR
GSM615315	32	IIIC	3	4	1	non-pCR
GSM615317	47	IIIC	3	2	1	non-pCR
GSM615319	63	IIIC	3	2	1	non-pCR
GSM615320	40	IIIA	3	2	1	non-pCR
GSM615321	50	IIB	3	2	1	non-pCR
GSM615322	52	IIIB	3	4	1	non-pCR
GSM615324	49	IIA	3	1	1	pCR
GSM615325	43	IIIC	3	1	1	pCR
GSM615326	57	IIA	3	2	0	non-pCR
GSM615327	65	IIB	3	3	0	non-pCR
GSM615330	65	IIIA	3	3	1	non-pCR
GSM615331	61	IIB	3	2	1	pCR
GSM615333	48	I	2	1	0	non-pCR
GSM615334	61	IIIB	3	3	1	pCR
GSM615335	51	IIB	3	2	1	non-pCR
GSM615336	49	IIB	3	2	1	pCR
GSM615338	57	IIB	3	2	1	pCR
GSM615346	42	IIB	3	2	1	non-pCR
GSM615347	72	IIA	3	2	0	non-pCR
GSM615348	33	IIB	3	3	0	non-pCR
GSM615354	46	IIIB	2	2	1	pCR
GSM615357	53	IIB	3	2	1	pCR
GSM615359	51	IIIB	3	4	1	pCR
GSM615363	36	IIA	3	2	0	non-pCR
GSM615364	34	IIIA	3	2	1	non-pCR
GSM615368	28	IIB	3	2	1	non-pCR

GSM615369	50	IIIA	3	2	1	non-pCR
GSM615371	67	IIB	3	2	1	pCR
GSM615372	39	IIB	3	2	1	non-pCR
GSM615375	75	IIIA	3	2	1	pCR
GSM615376	51	IIB	2	2	1	non-pCR
GSM615378	41	IIIB	3	2	1	non-pCR
GSM615379	62	IIIB	3	4	1	pCR
GSM615380	40	IIIA	3	3	1	non-pCR
GSM615382	59	IIA	3	2	0	non-pCR
GSM615384	57	IIIA	3	3	1	non-pCR
GSM615385	37	IIIA	2	3	1	non-pCR
GSM615387	38	IIB	3	2	1	non-pCR
GSM615390	46	IIIA	3	2	1	non-pCR
GSM615392	65	IIIB	3	4	1	non-pCR
GSM615393	43	IIIA	3	3	1	pCR
GSM615394	62	IIA	3	2	0	pCR
GSM615396	32	IIIA	3	3	1	pCR
GSM615397	57	IIB	2	2	1	pCR
GSM615403	50	IIIB	2	4	1	non-pCR
GSM615632	42	IIB	3	2	1	pCR
GSM615637	34	IIB	3	3	0	pCR
GSM615638	58	IIB	3	2	1	non-pCR
GSM615639	43	IIIA	3	3	1	pCR
GSM615640	49	IIB	3	3	0	non-pCR
GSM615641	40	IIB	3	2	1	non-pCR
GSM615643	65	IIIA	2	3	1	non-pCR
GSM615644	50	IIIA	3	3	1	pCR
GSM615648	59	IIB	3	3	0	non-pCR
GSM615649	65	IIIA	3	3	1	non-pCR
GSM615650	60	IIB	3	3	0	non-pCR
GSM615651	61	IIB	2	2	1	non-pCR
GSM615657	44	IIIA	3	3	1	non-pCR
GSM615658	50	IIB	3	2	1	pCR
GSM615660	41	IIIC	3	2	1	pCR
GSM615661	62	IIB	3	2	1	non-pCR
GSM615666	67	IIIA	3	3	1	non-pCR
GSM615667	56	IIIA	3	3	1	non-pCR
GSM615668	40	IIIA	3	2	1	pCR
GSM615671	39	IIB	2	3	0	non-pCR
GSM615674	33	IIA	3	1	1	non-pCR
GSM615676	47	IIB	3	2	1	pCR
GSM615677	46	IIIC	3	3	1	pCR
GSM615680	48	IIB	3	3	0	pCR
GSM615687	39	IIB	3	3	0	non-pCR
GSM615689	40	IIB	3	3	0	non-pCR

GSM615691	34	IIB	3	2	1	non-pCR
GSM615694	40	IIB	3	2	1	pCR
GSM615695	72	IIIA	3	3	1	pCR
GSM615696	65	IIB	3	2	1	non-pCR
GSM615699	47	IIIA	3	3	1	non-pCR
GSM615701	53	IIB	3	2	1	non-pCR
GSM615706	43	IIIA	2	3	1	pCR
GSM615707	63	IIA	3	2	0	non-pCR
GSM615712	57	IIB	3	2	1	non-pCR
GSM615714	54	IIB	3	2	1	non-pCR
GSM615715	50	IIA	3	2	0	non-pCR
GSM615716	33	IIIA	3	3	1	non-pCR
GSM615727	24	IIIA	3	2	1	pCR
GSM615728	45	IIB	3	2	1	non-pCR
GSM615730	47	IIIB	3	4	0	non-pCR
GSM615732	24	IIIA	3	3	1	non-pCR
GSM615733	64	IIA	3	2	0	pCR
GSM615737	50	IIA	3	2	0	pCR
GSM615739	53	IIB	3	3	0	non-pCR
GSM615741	44	IIA	3	2	0	non-pCR
GSM615742	38	IIIC	3	2	1	pCR
GSM615748	60	IIA	3	2	0	pCR
GSM615755	31	IIIA	3	3	1	non-pCR
GSM615773	58	IIB	2	2	1	pCR
GSM615776	45	IIIA	3	3	1	non-pCR
GSM615786	55	IIIB	3	4	1	non-pCR
GSM615787	46	IIIA	3	3	1	non-pCR
GSM615794	66	IIIB	2	2	1	non-pCR
GSM615798	40	IIIA	3	3	1	pCR
GSM615800	40	IIIB	3	4	0	non-pCR
GSM615801	59	IIIC	2	4	1	non-pCR
GSM615804	35	IIIA	3	3	1	non-pCR
GSM615813	68	IIB	3	3	0	non-pCR
GSM615815	37	IIA	2	2	0	non-pCR
GSM809185	56	IIIA	3	3	1	pCR
GSM809186	67	IIIA	2	3	1	non-pCR
GSM809204	30	IIA	2	2	0	non-pCR
GSM809208	50	IIB	2	2	1	non-pCR
GSM809209	43	IIIA	3	3	1	non-pCR
GSM809214	42	IIB	2	2	1	non-pCR
GSM809220	58	IIB	1	2	1	non-pCR
GSM809224	63	IIA	2	2	0	non-pCR
GSM809225	61	IIB	3	2	1	pCR
GSM809227	49	IIB	2	2	1	non-pCR
GSM809230	42	IIA	2	2	0	non-pCR

GSM809231	28	IIB	2	2	1	pCR
GSM809232	68	IIB	2	2	1	pCR
GSM809237	63	IIB	3	2	1	pCR
GSM809239	68	IIB	2	2	1	non-pCR
GSM809247	54	IIIB	3	4	1	non-pCR
GSM809248	36	IIB	2	2	1	pCR
GSM809251	61	IIB	2	2	1	non-pCR
GSM809255	62	IIB	3	2	1	non-pCR
GSM809266	70	IIB	2	2	1	pCR
GSM809273	63	IIIA	3	3	1	non-pCR
GSM809274	53	IIA	3	1	1	pCR
GSM809277	36	IIA	3	2	0	non-pCR
GSM809278	63	IIB	2	2	1	non-pCR
GSM809280	60	IIB	3	2	1	pCR
GSM809295	68	IIB	3	2	1	pCR

Table S4. Differentially expressed genes of molecular phenotypes of TNBC

Gene	logFC	AveExpr	t	P. Value	adj.P. Val	B	threshold
SMARCC2	5.011	8.467	16.496	2.15E-40	3.20E-37	80.938	Up
VHL	10.421	6.175	15.842	2.78E-38	2.06E-35	76.167	Up
KANSL3	6.896	5.562	14.856	4.36E-35	1.62E-32	68.940	Up
SOX3	7.548	5.881	12.870	1.16E-28	2.15E-26	54.403	Up
NPAS3	5.813	6.727	11.378	6.49E-24	4.20E-22	43.653	Up
SYN1	5.888	6.173	11.336	8.79E-24	5.44E-22	43.355	Up
RBM8A	3.243	10.104	11.173	2.85E-23	1.69E-21	42.199	Up
OTOF	5.198	4.029	10.772	5.03E-22	2.37E-20	39.377	Up
DCAF13	4.574	6.580	10.595	1.77E-21	7.97E-20	38.141	Up
SYCE1L	7.363	6.098	10.482	3.94E-21	1.54E-19	37.355	Up
TK1	3.365	9.776	10.341	1.07E-20	3.78E-19	36.376	Up
HP1BP3	5.281	6.273	10.326	1.18E-20	4.02E-19	36.277	Up
AKT2	4.513	7.126	10.292	1.50E-20	4.96E-19	36.042	Up
SCARB1	3.178	9.457	9.874	2.76E-19	8.22E-18	33.180	Up
MFI2	5.095	6.007	9.776	5.44E-19	1.50E-17	32.516	Up
ZKSCAN1	3.614	9.326	9.622	1.57E-18	4.18E-17	31.473	Up
TONSL	5.050	3.563	9.526	3.02E-18	7.88E-17	30.832	Up
FRMD8	4.370	6.186	9.512	3.33E-18	8.54E-17	30.736	Up
FUT6	4.783	9.630	9.403	6.99E-18	1.70E-16	30.009	Up
HLA.F.AS1	3.198	8.266	9.337	1.09E-17	2.62E-16	29.571	Up
RARS2	6.826	4.803	9.144	4.02E-17	9.06E-16	28.293	Up
MYL12A	2.255	11.189	9.063	6.93E-17	1.49E-15	27.760	Up
ASMTL.AS1	4.457	3.415	8.928	1.71E-16	3.63E-15	26.875	Up
CYP2U1	4.140	4.088	8.918	1.82E-16	3.81E-15	26.812	Up
EML4	2.317	8.696	8.906	1.97E-16	4.07E-15	26.733	Up
JPH2	3.760	3.717	8.843	2.99E-16	5.92E-15	26.327	Up
SREBF1	3.280	9.137	8.778	4.59E-16	8.76E-15	25.905	Up
FIP1L1	3.193	8.111	8.544	2.13E-15	3.76E-14	24.404	Up
SLC26A1	3.578	7.031	8.448	3.97E-15	6.94E-14	23.792	Up
BAGE	4.639	5.857	8.359	7.03E-15	1.20E-13	23.232	Up
BSDC1	1.634	9.389	8.277	1.19E-14	1.99E-13	22.714	Up
KCNK15	4.907	4.270	7.834	1.96E-13	2.78E-12	19.976	Up
CNP	1.904	10.320	7.828	2.04E-13	2.86E-12	19.938	Up
ETV3	5.129	4.816	7.761	3.08E-13	4.13E-12	19.534	Up
CAMTA1	6.174	5.674	7.621	7.33E-13	9.15E-12	18.687	Up
CYB5R3	2.243	11.545	7.530	1.28E-12	1.55E-11	18.142	Up
SEC23IP	1.707	8.671	7.520	1.36E-12	1.61E-11	18.085	Up
HEATR3	2.672	7.917	7.504	1.50E-12	1.75E-11	17.989	Up
NOL3	2.906	8.350	7.455	2.02E-12	2.34E-11	17.698	Up
EXOSC1	5.195	5.335	7.381	3.15E-12	3.60E-11	17.263	Up
TMEM80	2.400	7.546	7.268	6.19E-12	6.72E-11	16.603	Up
RP2	2.714	6.416	7.249	6.95E-12	7.43E-11	16.490	Up

ELL	2.699	7.411	7.217	8.39E-12	8.92E-11	16.306	Up
STMN1	3.740	9.089	7.214	8.56E-12	9.02E-11	16.287	Up
ECE2	2.978	7.481	7.116	1.53E-11	1.59E-10	15.718	Up
KIF26B	4.437	4.584	7.032	2.51E-11	2.54E-10	15.237	Up
GOLGA3	2.070	9.004	7.022	2.66E-11	2.67E-10	15.182	Up
TMLHE	3.315	5.517	6.991	3.19E-11	3.10E-10	15.004	Up
MAP3K6	3.884	7.506	6.971	3.59E-11	3.44E-10	14.890	Up
KIAA1731	4.949	4.300	6.892	5.68E-11	5.34E-10	14.443	Up
FBXO22	3.733	5.191	6.834	7.90E-11	7.29E-10	14.121	Up
ICK	3.203	6.172	6.823	8.42E-11	7.72E-10	14.059	Up
ANKRD36BP2	6.512	3.634	6.823	8.46E-11	7.72E-10	14.054	Up
RAI1	4.199	5.332	6.752	1.26E-10	1.13E-09	13.663	Up
SLC25A28	2.581	8.555	6.740	1.36E-10	1.20E-09	13.592	Up
NBR1	1.429	9.502	6.733	1.41E-10	1.24E-09	13.554	Up
ZDHHC14	3.247	6.243	6.726	1.47E-10	1.29E-09	13.515	Up
GIT2	3.789	5.944	6.725	1.48E-10	1.29E-09	13.509	Up
DDX51	4.029	4.967	6.675	1.96E-10	1.66E-09	13.235	Up
ZNF24	1.318	10.512	6.670	2.03E-10	1.69E-09	13.204	Up
NT5C	2.688	7.395	6.661	2.13E-10	1.77E-09	13.155	Up
THRA	1.793	8.114	6.654	2.21E-10	1.82E-09	13.120	Up
TNK2	2.682	8.174	6.521	4.67E-10	3.67E-09	12.393	Up
ZNF254	3.980	6.093	6.510	4.96E-10	3.86E-09	12.333	Up
MICALL2	3.455	8.062	6.407	8.79E-10	6.51E-09	11.777	Up
SLC2A4RG	2.895	9.360	6.394	9.46E-10	6.96E-09	11.707	Up
RANBP10	1.365	8.459	6.343	1.25E-09	9.10E-09	11.432	Up
CARS2	2.964	9.435	6.333	1.33E-09	9.57E-09	11.378	Up
CCDC57	3.109	5.618	6.275	1.82E-09	1.30E-08	11.070	Up
NOS1AP	1.876	4.902	6.168	3.26E-09	2.24E-08	10.505	Up
MTSS1L	4.503	4.458	6.101	4.67E-09	3.14E-08	10.157	Up
TNXB	2.352	7.049	6.074	5.40E-09	3.61E-08	10.017	Up
DTYMK	2.322	8.817	6.021	7.16E-09	4.69E-08	9.743	Up
CXorf36	2.408	5.391	6.013	7.47E-09	4.87E-08	9.701	Up
BCL2L11	2.653	7.235	5.949	1.05E-08	6.77E-08	9.375	Up
SYMPK	3.377	6.952	5.945	1.07E-08	6.85E-08	9.354	Up
RRP8	1.745	8.432	5.892	1.41E-08	8.99E-08	9.088	Up
ATP6V0A2	2.056	7.219	5.851	1.74E-08	1.10E-07	8.881	Up
CCDC144A	4.105	4.544	5.841	1.84E-08	1.16E-07	8.828	Up
RARA	2.712	7.549	5.809	2.17E-08	1.36E-07	8.669	Up
SLC38A10	2.397	9.175	5.796	2.32E-08	1.44E-07	8.604	Up
ERICH1	4.082	5.000	5.792	2.37E-08	1.47E-07	8.582	Up
VASH1	1.933	8.035	5.791	2.38E-08	1.47E-07	8.581	Up
PTCD2	2.434	5.593	5.770	2.65E-08	1.63E-07	8.475	Up
FRMD4A	2.363	7.912	5.764	2.73E-08	1.67E-07	8.446	Up
C2	2.853	8.484	5.739	3.11E-08	1.87E-07	8.321	Up
ZNF215	2.084	4.860	5.705	3.72E-08	2.20E-07	8.149	Up

ASTN2	2.711	6.307	5.699	3.82E-08	2.25E-07	8.123	Up
RASAL2	3.277	6.441	5.694	3.93E-08	2.31E-07	8.094	Up
ZBTB44	3.729	5.710	5.679	4.23E-08	2.47E-07	8.024	Up
ZNF787	3.232	5.780	5.669	4.45E-08	2.58E-07	7.974	Up
DIAPH1	2.698	9.108	5.640	5.16E-08	2.96E-07	7.833	Up
CBS	2.770	8.587	5.596	6.46E-08	3.62E-07	7.615	Up
YEATS2	1.376	9.097	5.580	7.01E-08	3.90E-07	7.536	Up
EYA3	3.028	5.810	5.576	7.13E-08	3.96E-07	7.520	Up
C5orf42	3.883	5.455	5.562	7.66E-08	4.22E-07	7.450	Up
EXOSC10	1.735	8.443	5.526	9.16E-08	4.99E-07	7.278	Up
ATP10A	1.567	7.654	5.523	9.33E-08	5.06E-07	7.260	Up
AGBL3	4.299	3.486	5.500	1.05E-07	5.62E-07	7.149	Up
RAD54L2	2.898	6.080	5.497	1.06E-07	5.66E-07	7.139	Up
SMEK2	2.048	6.385	5.489	1.10E-07	5.87E-07	7.100	Up
ASPHD1	3.259	4.613	5.455	1.31E-07	6.89E-07	6.935	Up
MAPKBP1	2.790	6.331	5.423	1.53E-07	7.94E-07	6.782	Up
ESRRA	1.138	9.105	5.417	1.57E-07	8.12E-07	6.756	Up
BEST1	2.314	6.818	5.397	1.74E-07	8.91E-07	6.660	Up
PTRF	3.419	9.569	5.386	1.84E-07	9.34E-07	6.608	Up
ITGAX	3.267	5.969	5.369	2.00E-07	1.01E-06	6.526	Up
FOXC1	2.718	9.884	5.341	2.29E-07	1.15E-06	6.394	Up
STOML1	2.178	7.961	5.325	2.48E-07	1.24E-06	6.320	Up
RPAIN	2.889	7.100	5.324	2.49E-07	1.24E-06	6.316	Up
MUC4	2.977	5.493	5.296	2.86E-07	1.42E-06	6.182	Up
BCL2L2	1.184	9.018	5.271	3.21E-07	1.60E-06	6.069	Up
ZNF506	2.241	6.967	5.254	3.49E-07	1.73E-06	5.989	Up
PCNXL2	1.740	8.235	5.250	3.56E-07	1.76E-06	5.969	Up
TIE1	3.551	3.447	5.239	3.76E-07	1.85E-06	5.916	Up
LMNA	1.716	11.225	5.220	4.11E-07	2.01E-06	5.831	Up
RECQL4	3.718	6.729	5.220	4.12E-07	2.01E-06	5.830	Up
BLZF1	2.708	6.366	5.218	4.15E-07	2.02E-06	5.823	Up
UBE2O	2.988	6.645	5.202	4.48E-07	2.16E-06	5.749	Up
KLHL29	3.228	5.140	5.200	4.54E-07	2.18E-06	5.736	Up
SREK1IP1	2.987	7.170	5.194	4.66E-07	2.22E-06	5.710	Up
SUV39H2	3.247	6.318	5.193	4.68E-07	2.22E-06	5.707	Up
OR2H1	2.404	6.587	5.157	5.55E-07	2.62E-06	5.542	Up
APBB1IP	2.466	7.265	5.150	5.75E-07	2.71E-06	5.509	Up
MAP3K13	3.120	6.135	5.144	5.93E-07	2.78E-06	5.479	Up
ARHGAP22	2.315	7.192	5.138	6.08E-07	2.84E-06	5.456	Up
ARL3	1.869	8.931	5.107	7.07E-07	3.29E-06	5.311	Up
MGA	2.837	6.658	5.060	8.79E-07	4.04E-06	5.101	Up
PEX14	2.263	8.174	5.059	8.85E-07	4.05E-06	5.095	Up
UGGT2	2.015	8.079	5.008	1.12E-06	5.03E-06	4.867	Up
NEMF	1.590	9.410	4.983	1.26E-06	5.60E-06	4.755	Up
OTUD7B	3.056	4.454	4.965	1.37E-06	6.05E-06	4.675	Up

UBE2W	1.097	8.237	4.955	1.44E-06	6.33E-06	4.630	Up
BTBD7	3.401	5.881	4.952	1.45E-06	6.38E-06	4.619	Up
EFNA2	2.239	5.183	4.927	1.64E-06	7.13E-06	4.506	Up
ZNF493	3.204	5.939	4.924	1.66E-06	7.21E-06	4.492	Up
ARHGEF10	2.999	8.018	4.907	1.80E-06	7.76E-06	4.416	Up
ITGA1	3.907	4.572	4.901	1.84E-06	7.93E-06	4.394	Up
GON4L	1.137	9.800	4.886	1.97E-06	8.43E-06	4.326	Up
PHF20L1	1.273	8.445	4.856	2.27E-06	9.63E-06	4.194	Up
GPR125	1.659	8.793	4.795	2.99E-06	1.26E-05	3.928	Up
CCDC82	2.076	6.679	4.788	3.08E-06	1.29E-05	3.901	Up
SMPDL3B	3.616	4.873	4.785	3.13E-06	1.31E-05	3.885	Up
MEG3	2.567	6.761	4.781	3.18E-06	1.32E-05	3.871	Up
PGM5	2.300	4.632	4.750	3.66E-06	1.51E-05	3.735	Up
CNPY2	1.788	10.798	4.728	4.04E-06	1.66E-05	3.641	Up
PDE6B	2.371	4.798	4.721	4.16E-06	1.70E-05	3.613	Up
NOC2L	1.677	8.692	4.719	4.21E-06	1.71E-05	3.602	Up
COTL1	2.023	10.500	4.718	4.23E-06	1.71E-05	3.598	Up
ZNF446	2.429	6.671	4.710	4.37E-06	1.76E-05	3.567	Up
SETMAR	1.854	7.765	4.676	5.08E-06	2.03E-05	3.422	Up
PLXNA1	1.816	8.948	4.665	5.35E-06	2.13E-05	3.372	Up
PPP1R12B	1.163	8.592	4.647	5.80E-06	2.29E-05	3.297	Up
IL11RA	1.629	7.621	4.642	5.92E-06	2.34E-05	3.276	Up
CDC42BPB	3.041	7.264	4.626	6.34E-06	2.47E-05	3.211	Up
HGSNAT	1.717	9.210	4.611	6.77E-06	2.63E-05	3.148	Up
LMO7	2.721	5.799	4.594	7.30E-06	2.82E-05	3.077	Up
HPS4	2.115	7.574	4.582	7.68E-06	2.95E-05	3.027	Up
MFN2	1.617	9.555	4.571	8.08E-06	3.09E-05	2.980	Up
LMF1	2.266	6.745	4.554	8.68E-06	3.29E-05	2.911	Up
CHML	2.502	6.741	4.521	1.00E-05	3.79E-05	2.773	Up
RNFT2	2.727	6.241	4.516	1.02E-05	3.85E-05	2.754	Up
MCPH1	2.335	4.018	4.508	1.06E-05	3.98E-05	2.721	Up
ZAK	1.520	7.444	4.493	1.13E-05	4.23E-05	2.660	Up
BPNT1	2.865	6.208	4.483	1.18E-05	4.41E-05	2.617	Up
LTB	2.665	8.945	4.481	1.19E-05	4.43E-05	2.609	Up
RHOBTB2	1.964	5.955	4.462	1.29E-05	4.75E-05	2.533	Up
HCG18	1.955	7.102	4.450	1.36E-05	5.01E-05	2.481	Up
RNH1	2.367	9.947	4.416	1.57E-05	5.71E-05	2.347	Up
CD79A	2.091	8.347	4.397	1.70E-05	6.13E-05	2.270	Up
GPD1	2.178	5.628	4.385	1.80E-05	6.43E-05	2.219	Up
C10orf76	1.722	7.346	4.358	2.01E-05	7.15E-05	2.112	Up
SVEP1	1.879	6.115	4.350	2.08E-05	7.38E-05	2.081	Up
LOC389906	3.534	5.978	4.334	2.23E-05	7.88E-05	2.015	Up
BBS9	1.339	7.062	4.316	2.40E-05	8.44E-05	1.944	Up
DHX35	2.202	6.646	4.304	2.52E-05	8.80E-05	1.900	Up
KIAA0513	1.623	7.688	4.261	3.01E-05	0.000104909	1.729	Up

POFUT2	1.395	8.334	4.260	3.03E-05	0.000105277	1.723	Up
CDON	2.390	4.368	4.252	3.13E-05	0.000108369	1.694	Up
CDKL1	2.230	4.133	4.250	3.15E-05	0.00010907	1.685	Up
RBM14	1.939	8.651	4.240	3.28E-05	0.000112999	1.648	Up
ZNF573	1.716	7.373	4.229	3.44E-05	0.000117971	1.605	Up
PTBP2	1.418	8.241	4.227	3.47E-05	0.000118819	1.596	Up
IFIH1	2.592	8.709	4.222	3.54E-05	0.000121158	1.575	Up
ZAP70	2.413	6.661	4.221	3.56E-05	0.000121322	1.571	Up
MYH11	2.423	6.217	4.147	4.81E-05	0.0001629	1.286	Up
FOXN1	1.191	3.992	4.143	4.88E-05	0.000164935	1.272	Up
ZBTB3	2.124	6.310	4.122	5.31E-05	0.000178612	1.193	Up
TTC12	1.505	7.076	4.111	5.56E-05	0.000186655	1.149	Up
PTGIR	1.903	5.987	4.095	5.93E-05	0.000197999	1.089	Up
POLH	2.523	6.460	4.093	5.98E-05	0.000199402	1.080	Up
N4BP2L2	1.531	9.731	4.054	6.98E-05	0.000230138	0.934	Up
PAK1	2.391	6.208	4.039	7.39E-05	0.000242601	0.881	Up
XCR1	1.796	5.212	4.038	7.44E-05	0.000243717	0.874	Up
TPH1	2.596	3.803	4.027	7.76E-05	0.000253691	0.834	Up
TERT	1.848	5.569	3.996	8.77E-05	0.00028473	0.719	Up
C19orf66	1.500	9.439	3.987	9.08E-05	0.000294084	0.687	Up
PAPOLG	1.560	7.120	3.980	9.34E-05	0.000301111	0.660	Up
PRKCA	1.615	7.911	3.972	9.66E-05	0.000310141	0.629	Up
GAMT	2.767	5.642	3.959	0.000101426	0.000323697	0.582	Up
KPNA4	2.023	7.931	3.959	0.000101441	0.000323697	0.582	Up
EDN1	2.748	7.281	3.946	0.000106907	0.000340408	0.533	Up
TRAPPC10	2.139	7.120	3.937	0.000110375	0.000348466	0.503	Up
QTRTD1	1.775	7.253	3.894	0.000130787	0.000410294	0.343	Up
METTL8	2.980	6.574	3.881	0.000137204	0.00042714	0.298	Up
WDR60	2.353	5.861	3.881	0.000137305	0.00042714	0.298	Up
CDH7	1.552	3.671	3.859	0.000149319	0.000462578	0.219	Up
DYNC1H1	1.559	10.082	3.842	0.00015932	0.000491511	0.158	Up
KLF6	2.082	9.723	3.825	0.000170352	0.000521222	0.095	Up
LINS	2.630	5.614	3.806	0.000183188	0.000557058	0.027	Up
CASP8	1.439	8.653	3.752	0.000224541	0.000671817	-0.164	Up
RGS11	1.842	5.889	3.745	0.000229697	0.000684489	-0.185	Up
CLDND1	1.477	9.239	3.742	0.000232558	0.000691626	-0.197	Up
SCPEP1	1.206	10.111	3.729	0.000244099	0.000721621	-0.242	Up
BRF1	1.772	6.602	3.699	0.0002728	0.000801688	-0.346	Up
TEAD1	1.348	7.898	3.697	0.000275203	0.000807155	-0.354	Up
PEX13	1.999	7.775	3.664	0.00031106	0.000908696	-0.468	Up
KIAA0430	1.150	9.448	3.651	0.000326197	0.000943687	-0.513	Up
MAP4K4	1.101	8.724	3.640	0.000339768	0.000973046	-0.551	Up
NLRX1	1.654	8.017	3.639	0.000340272	0.000973046	-0.552	Up
MANEA	2.740	5.696	3.631	0.000350978	0.000999816	-0.581	Up
DNASE1	2.181	4.078	3.626	0.000356991	0.001013065	-0.597	Up

FABP6	2.237	3.507	3.614	0.000373535	0.001053978	-0.639	Up
NDUFA7	2.341	8.522	3.609	0.000380702	0.001072165	-0.657	Up
ACTR3B	1.630	8.749	3.599	0.000394924	0.001110116	-0.691	Up
SLC5A12	2.559	3.503	3.597	0.000397127	0.001113529	-0.696	Up
SSTR3	1.327	4.872	3.571	0.00043608	0.001216606	-0.783	Up
SSPN	2.056	7.376	3.523	0.000518642	0.001433494	-0.945	Up
MYO1D	2.604	6.177	3.478	0.000607479	0.001666645	-1.092	Up
ZNF428	2.042	5.515	3.474	0.00061698	0.001686487	-1.106	Up
SEPW1	1.050	11.545	3.457	0.000654872	0.001776998	-1.161	Up
RECQL5	1.300	7.111	3.449	0.000672911	0.001812715	-1.186	Up
SERPINB8	1.214	7.422	3.441	0.000691605	0.001849669	-1.212	Up
DNM2	1.155	8.242	3.440	0.000696283	0.001855508	-1.218	Up
CCDC88A	1.738	7.015	3.436	0.000705379	0.001876385	-1.230	Up
PAQR3	1.525	7.275	3.435	0.000707646	0.001878478	-1.233	Up
FGF7	1.472	6.921	3.424	0.000734802	0.001944218	-1.268	Up
ZCCHC10	1.300	7.427	3.413	0.000762858	0.002014867	-1.303	Up
CASC5	2.678	4.939	3.412	0.000766332	0.002020453	-1.307	Up
CARHSP1	1.689	8.453	3.405	0.000785782	0.002060771	-1.330	Up
MEF2BNB	1.632	7.261	3.403	0.000792093	0.002073666	-1.337	Up
DGKH	1.303	7.790	3.385	0.000842323	0.00220129	-1.394	Up
KCNE4	2.151	5.649	3.376	0.000868611	0.002258084	-1.423	Up
ESRRB	1.569	3.916	3.336	0.000996729	0.002557615	-1.550	Up
DTWD1	1.901	6.694	3.336	0.00099759	0.002557615	-1.551	Up
MCM3AP.AS1	1.093	7.396	3.330	0.001016374	0.002601287	-1.568	Up
NADSYN1	1.919	7.150	3.311	0.001086729	0.002776575	-1.630	Up
HIP1	1.018	7.696	3.300	0.001125154	0.002864903	-1.662	Up
PLCE1	1.696	6.414	3.296	0.001144131	0.002903282	-1.677	Up
ATP8B3	1.899	4.788	3.292	0.001156207	0.002918982	-1.687	Up
IQSEC3	1.546	5.541	3.270	0.001247972	0.003113649	-1.757	Up
GSN	1.043	10.685	3.222	0.001466496	0.003616385	-1.906	Up
HLA.DOB	2.274	6.962	3.201	0.0015722	0.003851501	-1.970	Up
SF3B2	1.647	9.533	3.194	0.001605669	0.003927022	-1.989	Up
VPS13B	1.020	8.755	3.181	0.001679655	0.004101226	-2.030	Up
MOXD1	1.151	8.318	3.155	0.001829094	0.00444422	-2.108	Up
CNTLN	1.699	6.353	3.150	0.001858518	0.004508346	-2.123	Up
ANKH	2.027	5.575	3.141	0.001911938	0.004630377	-2.149	Up
STXBP3	1.152	8.417	3.128	0.001996483	0.004819433	-2.189	Up
ZNF135	2.387	5.936	3.113	0.002095308	0.005049795	-2.233	Up
ATF2	1.113	7.708	3.103	0.00216252	0.005186559	-2.262	Up
KAZALD1	1.783	6.377	3.098	0.00220335	0.005267495	-2.279	Up
CDHR1	1.378	3.389	3.088	0.002276472	0.005424862	-2.309	Up
PRSS23	2.173	8.319	3.064	0.002459133	0.005822819	-2.379	Up
A2M	2.050	11.007	3.063	0.002464902	0.005827201	-2.381	Up
WNT4	2.017	5.260	3.055	0.002523984	0.005947962	-2.403	Up
PWP2	1.616	7.919	3.053	0.002543337	0.005984085	-2.410	Up

DSE	1.457	8.696	3.038	0.002670928	0.006244765	-2.455	Up
DYNC2LI1	1.085	7.287	3.034	0.002704184	0.006309053	-2.466	Up
ACVR2B	1.051	8.719	3.033	0.002706911	0.006309053	-2.467	Up
PELO	1.000	8.777	3.022	0.002807561	0.00650287	-2.500	Up
TRIB3	1.236	9.403	3.021	0.00281642	0.006513244	-2.503	Up
VCPIP1	1.733	6.326	2.974	0.003268951	0.007466866	-2.638	Up
PMP22	1.751	9.962	2.954	0.003471992	0.007894269	-2.693	Up
TIPRL	1.200	9.411	2.947	0.003556409	0.008037046	-2.715	Up
DNAJC3	1.347	7.804	2.946	0.00356084	0.008037046	-2.716	Up
WAPAL	1.030	9.068	2.938	0.003647913	0.008169348	-2.738	Up
S100B	2.667	5.565	2.923	0.003829059	0.008498224	-2.782	Up
SLC9A7	2.012	5.551	2.907	0.004014927	0.008884222	-2.825	Up
CYTH4	1.061	8.429	2.894	0.004189587	0.009256934	-2.863	Up
TP73	1.348	4.189	2.858	0.004672775	0.010203254	-2.962	Up
CHD2	1.546	7.892	2.849	0.004796593	0.010442949	-2.986	Up
FCHSD2	1.536	8.012	2.840	0.004938621	0.010705144	-3.012	Up
USP36	1.382	6.898	2.834	0.005025748	0.010878148	-3.028	Up
GEMIN7	1.175	7.288	2.833	0.005040951	0.010879382	-3.031	Up
ADAMTS5	1.099	7.861	2.822	0.005213886	0.011221787	-3.061	Up
CSRNP3	1.517	4.670	2.822	0.005214697	0.011221787	-3.061	Up
CLN6	2.444	6.326	2.819	0.005257807	0.011281904	-3.068	Up
MAOB	2.486	7.612	2.785	0.005818428	0.012360003	-3.160	Up
RNF170	1.272	7.026	2.777	0.005948827	0.012601006	-3.180	Up
NT5E	1.842	6.726	2.735	0.006738714	0.014123822	-3.292	Up
PSIP1	1.072	9.581	2.717	0.007108234	0.014786712	-3.339	Up
MS4A4A	1.658	8.141	2.717	0.007109952	0.014786712	-3.340	Up
ERAP2	2.127	7.049	2.703	0.007397102	0.015298318	-3.375	Up
ITGB8	1.163	7.222	2.700	0.007469078	0.01542572	-3.384	Up
PTPN21	1.392	6.435	2.675	0.008042947	0.016473639	-3.450	Up
TRPV6	1.818	5.798	2.647	0.008717397	0.017732928	-3.522	Up
MAGIX	1.686	5.184	2.632	0.009094087	0.01842358	-3.559	Up
PTPRM	1.251	8.398	2.594	0.010120787	0.020364831	-3.655	Up
PHKG2	1.000	8.320	2.587	0.010309814	0.020717154	-3.671	Up
SLC8A1	1.564	6.958	2.574	0.010694094	0.021431425	-3.703	Up
ACSL4	1.459	6.929	2.561	0.011115622	0.022220962	-3.738	Up
ARHGAP19	1.399	6.238	2.478	0.013958082	0.027490951	-3.939	Up
C1S	2.119	10.774	2.473	0.014142498	0.027780575	-3.950	Up
ANO1	1.701	7.148	2.471	0.014217863	0.02789177	-3.955	Up
BRWD1	1.298	7.141	2.451	0.015018992	0.029308716	-4.003	Up
EMP1	1.733	9.480	2.441	0.015447197	0.030065422	-4.028	Up
FUT8	1.760	7.342	2.438	0.015538997	0.030204561	-4.033	Up
VASH2	1.626	7.224	2.431	0.01583754	0.030695017	-4.050	Up
PDK4	1.519	6.068	2.431	0.015853243	0.030695017	-4.051	Up
CALML4	1.263	7.579	2.428	0.015968387	0.030814096	-4.057	Up
SCEL	1.925	3.691	2.427	0.016039065	0.030893898	-4.061	Up

LAMA3	1.591	6.285	2.422	0.01624159	0.031243524	-4.072	Up
SOX4	1.107	11.581	2.413	0.016622984	0.031935888	-4.092	Up
PTGFR	1.859	4.512	2.404	0.017037782	0.032606411	-4.114	Up
FGD6	1.291	6.992	2.363	0.018974797	0.036035151	-4.208	Up
PRR16	1.952	4.462	2.333	0.020529448	0.03878944	-4.277	Up
MBNL2	1.328	9.205	2.323	0.021099694	0.039816302	-4.300	Up
BAHCC1	1.295	6.838	2.318	0.02135965	0.040153982	-4.311	Up
SAMD14	1.008	6.050	2.315	0.021517621	0.040399109	-4.317	Up
CDC25A	1.175	6.410	2.314	0.021602361	0.040456815	-4.321	Up
CLIP1	1.042	8.877	2.311	0.021736644	0.040657093	-4.326	Up
SYF2	1.045	8.969	2.302	0.022288784	0.041530761	-4.348	Up
MPHOSPH9	1.823	5.364	2.300	0.022363984	0.041569056	-4.351	Up
PRMT5	1.147	9.185	2.292	0.022847322	0.042308802	-4.369	Up
PRKAA1	1.316	6.444	2.264	0.024550838	0.045126201	-4.432	Up
PIP4K2B	0.899	9.003	4.486	1.17E-05	4.36E-05	2.630	NoSig
C11orf57	-0.929	8.951	-4.325	2.31E-05	8.15E-05	1.980	NoSig
ADAMTS9	-0.933	8.585	-4.077	6.37E-05	0.00021148	1.021	NoSig
SSH1	-0.773	9.183	-4.048	7.13E-05	0.000234583	0.914	NoSig
MAN1A2	-0.846	10.158	-3.982	9.29E-05	0.000300229	0.665	NoSig
FUS	-0.782	10.732	-3.939	0.000109716	0.000347863	0.508	NoSig
KLHL18	-0.850	9.015	-3.830	0.000167195	0.000513676	0.113	NoSig
ZNF75D	0.925	8.046	3.826	0.000169651	0.000520148	0.099	NoSig
NFX1	-0.956	9.280	-3.784	0.000198807	0.000602089	-0.050	NoSig
TMEM134	0.839	9.496	3.782	0.000200231	0.00060517	-0.056	NoSig
TPCN1	0.898	9.352	3.765	0.000213298	0.000640755	-0.116	NoSig
TBC1D1	-0.914	9.387	-3.762	0.000215919	0.000647321	-0.127	NoSig
VPS13D	-0.745	8.738	-3.726	0.000246788	0.000728122	-0.252	NoSig
TATDN2	-0.880	9.837	-3.663	0.000311658	0.000908696	-0.470	NoSig
WFS1	-0.812	9.213	-3.654	0.000322746	0.000935523	-0.503	NoSig
ATP5SL	0.816	9.434	3.650	0.000327214	0.000944792	-0.516	NoSig
FBXO28	-0.912	9.686	-3.624	0.000359956	0.001019533	-0.605	NoSig
TRIOBP	-0.973	10.058	-3.487	0.000589048	0.001622064	-1.063	NoSig
WBSCR16	-0.832	8.978	-3.454	0.000661212	0.001790933	-1.170	NoSig
TPR	-0.884	11.386	-3.442	0.000689838	0.001849669	-1.209	NoSig
DDX3X	-0.958	11.351	-3.442	0.000690568	0.001849669	-1.210	NoSig
MINA	-0.890	9.224	-3.378	0.00086133	0.002243077	-1.415	NoSig
RUNX1T1	-0.987	8.685	-3.363	0.000909854	0.00235706	-1.466	NoSig
SREK1	-0.737	9.347	-3.354	0.000937265	0.002423849	-1.493	NoSig
FRYL	-0.978	9.625	-3.350	0.00094885	0.002445304	-1.504	NoSig
PVR	-0.721	8.613	-3.297	0.001138232	0.002893249	-1.672	NoSig
RANGAP1	-0.902	8.821	-3.285	0.001186002	0.002989126	-1.710	NoSig
PARVA	0.747	8.450	3.275	0.001227316	0.003077603	-1.742	NoSig
FGF1	-0.915	7.729	-3.270	0.001247301	0.003113649	-1.757	NoSig
SF1	0.701	10.409	3.263	0.001275351	0.003171315	-1.777	NoSig
PIGQ	0.951	7.534	3.262	0.001282507	0.003183787	-1.782	NoSig

RHEB	-0.944	12.090	-3.216	0.001496567	0.00368443	-1.924	NoSig
PGPEP1	-0.655	8.525	-3.135	0.001954438	0.004725609	-2.169	NoSig
ARHGEF7	-0.711	9.818	-3.099	0.002196488	0.005259545	-2.276	NoSig
SCRN3	0.954	6.993	3.093	0.002239968	0.005346439	-2.294	NoSig
TROAP	0.775	9.177	3.077	0.002355215	0.005594577	-2.340	NoSig
SAE1	0.655	10.385	3.071	0.00240237	0.005697487	-2.358	NoSig
MTMR11	0.974	8.846	3.060	0.002486177	0.005868167	-2.389	NoSig
TM2D1	0.883	9.068	3.048	0.002583355	0.00606864	-2.424	NoSig
YIF1B	0.829	7.803	3.042	0.002633512	0.006176707	-2.442	NoSig
GDAP2	-0.633	8.085	-3.025	0.002780631	0.006460622	-2.491	NoSig
BBX	0.725	9.457	2.990	0.003108249	0.007143687	-2.593	NoSig
SYNCRIP	-0.810	11.540	-2.983	0.00317033	0.007275124	-2.611	NoSig
PMS1	-0.782	9.328	-2.968	0.003322309	0.007577107	-2.653	NoSig
PRKAB2	0.653	8.663	2.947	0.00354937	0.008037046	-2.713	NoSig
PBLD	-0.960	7.176	-2.944	0.003582238	0.008058681	-2.722	NoSig
TRIM44	-0.954	9.773	-2.936	0.003670514	0.0082076	-2.744	NoSig
ORAI2	0.819	8.145	2.892	0.004206513	0.00928054	-2.867	NoSig
ACP1	-0.830	10.630	-2.888	0.004257639	0.009379421	-2.878	NoSig
ABI1	0.856	9.931	2.887	0.004269813	0.009392325	-2.881	NoSig
FAM178A	-0.745	7.894	-2.885	0.004294946	0.009433656	-2.886	NoSig
CHD1L	-0.871	10.597	-2.877	0.004411066	0.00966017	-2.910	NoSig
FBXO7	-0.903	11.382	-2.867	0.004539322	0.00992643	-2.936	NoSig
TTC23	-0.755	8.261	-2.833	0.005036743	0.010879382	-3.030	NoSig
PSMD10	-0.837	10.389	-2.820	0.00524306	0.011266517	-3.066	NoSig
ARID5B	-0.847	10.360	-2.805	0.00547268	0.011699815	-3.105	NoSig
DLGAP4	0.626	8.021	2.782	0.005866074	0.01244344	-3.167	NoSig
RMND1	-0.871	7.590	-2.765	0.006169163	0.013030604	-3.212	NoSig
CLASP2	0.831	8.382	2.763	0.006217009	0.013113038	-3.219	NoSig
HIPK1	0.728	9.111	2.754	0.006385868	0.013450121	-3.243	NoSig
DCAF8	-0.582	9.171	-2.747	0.006508596	0.013689225	-3.260	NoSig
KIAA0226	0.634	8.923	2.741	0.006623674	0.013911587	-3.276	NoSig
OGDH	-0.715	8.630	-2.730	0.006847234	0.014320446	-3.306	NoSig
SNAPC5	-0.844	9.494	-2.721	0.007032032	0.014679999	-3.330	NoSig
FBXO42	0.869	8.123	2.720	0.007038897	0.014679999	-3.331	NoSig
TM2D3	0.729	10.739	2.714	0.007161619	0.014873362	-3.346	NoSig
PIGK	0.837	8.236	2.705	0.007368038	0.015280716	-3.372	NoSig
USP25	-0.557	10.027	-2.704	0.007379031	0.015282199	-3.373	NoSig
PPARA	0.947	6.974	2.698	0.007510135	0.015489003	-3.389	NoSig
TSC22D4	0.720	7.848	2.695	0.007575847	0.015602887	-3.396	NoSig
SMC3	0.829	9.067	2.694	0.007600433	0.015631873	-3.399	NoSig
NAP1L1	-0.832	11.783	-2.667	0.008211953	0.016796662	-3.468	NoSig
DDX6	0.869	7.911	2.649	0.008647863	0.017625591	-3.515	NoSig
PDE8B	-0.890	8.038	-2.645	0.008745148	0.017765075	-3.525	NoSig
RCBTB1	0.796	7.511	2.633	0.009057513	0.018374518	-3.556	NoSig
RNF130	0.745	10.293	2.620	0.009404626	0.019026773	-3.589	NoSig

WDR78	-0.719	7.298	-2.550	0.011463178	0.022880195	-3.765	NoSig
CRYZL1	-0.869	8.177	-2.545	0.011602411	0.023096098	-3.776	NoSig
ITGB1	0.811	12.693	2.540	0.011760309	0.023379116	-3.788	NoSig
PGLS	0.760	9.649	2.523	0.012339077	0.02449694	-3.830	NoSig
MAF	0.996	8.136	2.505	0.012964725	0.025704728	-3.874	NoSig
SLC30A5	0.817	8.468	2.484	0.013749089	0.027151255	-3.926	NoSig
ZNF7	0.894	8.448	2.480	0.013898362	0.027409635	-3.935	NoSig
MKL1	-0.591	8.552	-2.474	0.01410568	0.027744903	-3.948	NoSig
SKIV2L2	0.620	9.190	2.456	0.01483936	0.029034379	-3.993	NoSig
RBBP6	0.589	9.693	2.453	0.014953494	0.029219244	-3.999	NoSig
MLLT10	0.594	8.761	2.442	0.015388657	0.029990737	-4.025	NoSig
SYNE2	-0.816	9.383	-2.434	0.015731049	0.03053795	-4.044	NoSig
PDXK	0.868	10.585	2.429	0.015926463	0.030796685	-4.055	NoSig
KAT6A	-0.759	10.214	-2.409	0.01681132	0.032256042	-4.102	NoSig
MAP3K2	0.572	8.003	2.409	0.016836437	0.032262606	-4.104	NoSig
AVL9	-0.942	8.732	-2.373	0.018515936	0.035298969	-4.187	NoSig
BTN2A2	0.558	9.126	2.365	0.018885388	0.035911217	-4.204	NoSig
MITF	0.690	7.837	2.341	0.020101599	0.038077806	-4.258	NoSig
RIN3	-0.875	8.404	-2.333	0.020521492	0.03878944	-4.276	NoSig
C14orf159	0.932	8.034	2.319	0.021309009	0.04010949	-4.309	NoSig
CSAD	-0.863	7.966	-2.308	0.0219325	0.040971894	-4.334	NoSig
PRR14	-0.506	10.162	-2.304	0.022122753	0.041275451	-4.342	NoSig
ATG2B	0.927	7.386	2.301	0.022315453	0.041530761	-4.349	NoSig
OSMR	-0.839	7.729	-2.297	0.022526365	0.041818608	-4.357	NoSig
RNF103	-0.746	9.720	-2.294	0.022758379	0.042196645	-4.366	NoSig
WWP2	-0.804	9.295	-2.291	0.022928623	0.042406545	-4.373	NoSig
ABHD11	0.810	9.800	2.282	0.023427357	0.04327513	-4.391	NoSig
ENTPD4	-0.794	9.088	-2.271	0.024110094	0.04448103	-4.416	NoSig
MYO16	-0.914	4.434	-2.266	0.024420685	0.044958933	-4.427	NoSig
DDX10	0.693	9.262	2.259	0.024828046	0.045523187	-4.441	NoSig
ERC1	-0.527	9.071	-2.246	0.025671807	0.047012286	-4.470	NoSig
UBTF	-0.828	8.257	-2.237	0.026294119	0.048061022	-4.491	NoSig
MKNK1	-0.571	9.505	-2.227	0.026932176	0.049138829	-4.512	NoSig
RABGAP1L	0.898	9.235	2.219	0.027477671	0.050011379	-4.529	NoSig
RPS6KA5	1.011	7.960	2.218	0.027540123	0.050063768	-4.531	NoSig
PCSK6	-0.917	7.651	-2.214	0.027848379	0.050562319	-4.540	NoSig
UBE3C	-0.604	9.545	-2.213	0.027903802	0.050601162	-4.542	NoSig
GRK4	-0.992	6.461	-2.212	0.028003427	0.050719971	-4.545	NoSig
GPR173	-1.147	6.055	-2.210	0.028161891	0.050944929	-4.550	NoSig
SLC9A5	1.090	6.446	2.203	0.028602905	0.051679855	-4.563	NoSig
MAPK12	-0.970	7.214	-2.203	0.028660359	0.051720817	-4.565	NoSig
FANCC	-0.592	9.074	-2.201	0.028804559	0.051918035	-4.569	NoSig
CNTN1	-0.986	7.720	-2.196	0.029130286	0.052441568	-4.579	NoSig
C4orf27	0.764	9.349	2.193	0.029357205	0.052786172	-4.586	NoSig
DDX31	1.040	6.128	2.187	0.02981763	0.053549295	-4.599	NoSig

KLK8	1.254	8.550	2.186	0.029883029	0.053602007	-4.601	NoSig
ETS1	1.167	7.394	2.180	0.030293412	0.054253777	-4.613	NoSig
UCHL1	1.531	9.462	2.180	0.03031936	0.054253777	-4.613	NoSig
LAPTM4B	-1.012	12.664	-2.178	0.030496389	0.054504964	-4.618	NoSig
BAP1	-0.696	8.868	-2.172	0.030952424	0.055253607	-4.631	NoSig
CNR1	-1.107	6.353	-2.169	0.031148111	0.055511777	-4.636	NoSig
SHB	-0.710	8.800	-2.169	0.031171711	0.055511777	-4.637	NoSig
SPTLC1	-0.783	10.238	-2.168	0.031262931	0.05560763	-4.640	NoSig
ABCB9	-1.206	6.370	-2.167	0.031329962	0.055660279	-4.641	NoSig
IKZF2	1.287	6.959	2.165	0.031424242	0.055761155	-4.644	NoSig
PTK6	1.715	5.192	2.162	0.031708081	0.056197755	-4.652	NoSig
UNC5C	-1.219	5.639	-2.151	0.032535612	0.057518023	-4.674	NoSig
CD209	-0.694	7.874	-2.151	0.032554241	0.057518023	-4.674	NoSig
MEX3C	1.322	8.127	2.151	0.032569049	0.057518023	-4.675	NoSig
SCARF1	1.068	6.285	2.150	0.032666113	0.057549205	-4.677	NoSig
TRIM36	1.207	5.600	2.150	0.032667891	0.057549205	-4.677	NoSig
SNX19	1.025	7.954	2.149	0.03273765	0.057549205	-4.679	NoSig
FCER1G	-0.882	10.648	-2.149	0.032741511	0.057549205	-4.679	NoSig
ZBTB40	0.736	8.752	2.144	0.033163722	0.058222496	-4.690	NoSig
COL9A1	1.252	4.733	2.132	0.034111549	0.059815888	-4.714	NoSig
NBEAL2	0.795	8.018	2.118	0.035307905	0.061840818	-4.744	NoSig
C2CD3	0.522	8.486	2.114	0.035621914	0.062317395	-4.751	NoSig
HNRNPM	0.711	11.876	2.110	0.036024194	0.062947093	-4.761	NoSig
STAB1	-0.682	9.531	-2.107	0.036228904	0.063230492	-4.765	NoSig
PHIP	1.097	9.514	2.105	0.036410128	0.063472286	-4.770	NoSig
DYRK3	0.915	7.601	2.105	0.036460734	0.063486078	-4.771	NoSig
ST18	1.434	3.639	2.097	0.037166988	0.06464013	-4.787	NoSig
SLC33A1	-0.594	9.108	-2.095	0.037304164	0.064802911	-4.790	NoSig
LIG3	-0.746	8.434	-2.088	0.037954864	0.06585634	-4.805	NoSig
HSPA4L	-0.915	7.320	-2.082	0.038528865	0.066774385	-4.818	NoSig
PLA2G4C	1.053	7.742	2.078	0.038828055	0.067214572	-4.824	NoSig
ZMYM6	-0.829	8.281	-2.062	0.040408005	0.06986826	-4.858	NoSig
SLC39A14	0.958	9.484	2.060	0.040572543	0.07007128	-4.861	NoSig
CARD8	-0.622	8.544	-2.054	0.041116048	0.070927568	-4.873	NoSig
NDUFS4	0.660	9.565	2.052	0.041323309	0.071202503	-4.877	NoSig
MYLK	1.580	8.878	2.052	0.041385925	0.07122786	-4.878	NoSig
DNAJC2	0.878	9.783	2.045	0.042024551	0.07224336	-4.891	NoSig
CLK4	-0.548	9.234	-2.041	0.042455626	0.072900134	-4.900	NoSig
CSF3R	-1.329	7.553	-2.039	0.042652559	0.073153813	-4.904	NoSig
SRC	0.848	6.086	2.038	0.042757747	0.073249734	-4.906	NoSig
AREG	1.381	6.434	2.029	0.04362591	0.074651011	-4.923	NoSig
HEATR6	0.611	7.513	2.028	0.043716795	0.074720546	-4.924	NoSig
TAF2	-0.650	9.922	-2.028	0.043795359	0.074768885	-4.926	NoSig
SUZ12	0.581	10.035	2.025	0.044107777	0.0752159	-4.932	NoSig
ATP5O	0.863	11.788	2.023	0.044262577	0.075393416	-4.935	NoSig

CEP68	-0.450	8.855	-2.022	0.044386524	0.075518033	-4.937	NoSig
MARK1	0.653	8.034	2.014	0.045180005	0.076780191	-4.952	NoSig
LUZP1	-1.027	8.182	-2.013	0.04533243	0.076939478	-4.955	NoSig
RBM6	0.492	9.237	2.013	0.045377217	0.076939478	-4.956	NoSig
KCTD17	0.972	6.066	2.006	0.046041563	0.077976999	-4.968	NoSig
YTHDC2	-0.760	8.284	-2.002	0.046542783	0.078736199	-4.977	NoSig
CDK12	-0.651	8.033	-1.998	0.046924382	0.07929154	-4.984	NoSig
MFAP3	-0.681	8.431	-1.995	0.047264839	0.079776181	-4.990	NoSig
RGS13	-1.424	5.501	-1.994	0.047418241	0.079944358	-4.993	NoSig
CARD14	0.679	7.811	1.985	0.048374408	0.081464036	-5.010	NoSig
NRP2	-0.585	8.098	-1.983	0.048615524	0.081777471	-5.014	NoSig
CUX2	-1.111	5.424	-1.976	0.049435592	0.083062967	-5.028	NoSig
UROS	-0.574	8.765	-1.975	0.049530433	0.08312839	-5.029	NoSig
SPAG9	-0.514	9.241	-1.964	0.050815544	0.085109262	-5.051	NoSig
ARMC8	-0.503	9.032	-1.964	0.050825168	0.085109262	-5.051	NoSig
HERC6	1.089	8.580	1.960	0.051219443	0.085641585	-5.057	NoSig
EAF2	0.978	7.436	1.960	0.051258245	0.085641585	-5.058	NoSig
PHKA2	0.777	8.498	1.959	0.051404068	0.085788832	-5.060	NoSig
APOL6	0.804	9.178	1.946	0.052908091	0.088199923	-5.084	NoSig
TFB2M	0.788	9.245	1.946	0.052978052	0.088217653	-5.086	NoSig
FYCO1	0.539	7.937	1.945	0.053094983	0.088313468	-5.087	NoSig
SMPDL3A	0.759	8.001	1.940	0.053663781	0.089159825	-5.096	NoSig
PPIL6	0.920	4.746	1.929	0.054997204	0.091273261	-5.117	NoSig
TBC1D8B	-0.598	8.083	-1.928	0.055198077	0.091504504	-5.120	NoSig
ULK2	0.512	7.807	1.914	0.056958451	0.094317613	-5.146	NoSig
PDGFRA	1.332	7.500	1.907	0.057765048	0.09554686	-5.157	NoSig
MTHFD2L	-0.997	6.234	-1.903	0.058395995	0.09648316	-5.166	NoSig
DDX50	0.644	9.745	1.897	0.059110057	0.097554556	-5.177	NoSig
TCTN1	0.606	8.520	1.894	0.059588462	0.09823508	-5.183	NoSig
RALGAPA1	0.890	8.103	1.892	0.05975125	0.098394362	-5.185	NoSig
SIM2	-1.005	7.406	-1.888	0.060278597	0.099152958	-5.193	NoSig
DZANK1	1.078	6.884	1.887	0.060484122	0.099381093	-5.196	NoSig
ATP9B	0.728	7.546	1.883	0.060988471	0.100099179	-5.202	NoSig
GFOD1	0.519	9.350	1.875	0.062105308	0.101819838	-5.217	NoSig
MPDU1	1.021	7.569	1.869	0.06299895	0.103171187	-5.229	NoSig
RPE	0.706	7.898	1.863	0.063733517	0.104220042	-5.239	NoSig
INPP4B	0.929	6.407	1.863	0.063779582	0.104220042	-5.239	NoSig
CALCOCO2	-0.523	9.676	-1.861	0.064087064	0.104607534	-5.243	NoSig
CPEB3	1.287	5.223	1.857	0.064657707	0.105423257	-5.251	NoSig
MKS1	-0.499	7.965	-1.853	0.065155733	0.106048211	-5.257	NoSig
PERP	0.964	10.542	1.853	0.065183635	0.106048211	-5.257	NoSig
BIN3	0.492	8.769	1.850	0.065596433	0.106603165	-5.262	NoSig
PACS2	-0.431	8.841	-1.849	0.065816278	0.106843674	-5.265	NoSig
SNX13	-0.429	8.906	-1.842	0.066795169	0.108314522	-5.277	NoSig
PARK2	-0.932	6.444	-1.840	0.067107459	0.108702388	-5.281	NoSig

ARPC5	-0.387	12.204	-1.831	0.068406131	0.110685437	-5.297	NoSig
OSGIN2	0.604	8.097	1.825	0.069288276	0.111990942	-5.307	NoSig
RNF216	-0.461	8.468	-1.824	0.069427344	0.112093877	-5.309	NoSig
RGS5	1.215	7.731	1.819	0.070262872	0.113319838	-5.319	NoSig
FKBP15	-0.399	9.794	-1.818	0.070480901	0.11354832	-5.321	NoSig
HINT1	0.517	12.865	1.816	0.070745423	0.113851129	-5.324	NoSig
GABRG2	-1.024	6.346	-1.807	0.072043405	0.115814641	-5.339	NoSig
EPB41	1.463	4.948	1.789	0.074925017	0.120316955	-5.371	NoSig
SETD6	0.961	8.160	1.786	0.075464137	0.12085378	-5.377	NoSig
ANKFY1	0.380	8.342	1.786	0.075547679	0.12085378	-5.378	NoSig
CTNND2	0.869	6.614	1.785	0.075575092	0.12085378	-5.378	NoSig
PAX7	0.775	4.530	1.785	0.075584908	0.12085378	-5.378	NoSig
BEND5	-0.570	7.142	-1.785	0.075665682	0.12085378	-5.379	NoSig
HDAC4	0.747	7.390	1.783	0.07589202	0.121085229	-5.382	NoSig
ARHGAP25	0.791	8.768	1.782	0.076156956	0.1213777	-5.385	NoSig
RABEPK	0.626	8.670	1.780	0.076408	0.121522712	-5.387	NoSig
COL1A1	1.630	11.520	1.780	0.076529543	0.121522712	-5.389	NoSig
STK24	-1.101	11.463	-1.779	0.076560631	0.121522712	-5.389	NoSig
SART3	-0.563	9.074	-1.779	0.076574836	0.121522712	-5.389	NoSig
C19orf26	1.076	2.981	1.773	0.077579734	0.122986209	-5.400	NoSig
NRP1	1.105	7.812	1.767	0.078646569	0.124544673	-5.411	NoSig
ST7L	0.949	6.080	1.764	0.079191986	0.125274982	-5.416	NoSig
DISC1	0.661	6.983	1.754	0.08086647	0.127787928	-5.433	NoSig
TXLNG	0.698	7.314	1.743	0.08269399	0.130537117	-5.451	NoSig
MLXIP	0.499	9.269	1.739	0.083445999	0.131584517	-5.459	NoSig
FLCN	0.929	7.364	1.737	0.083794048	0.131993379	-5.462	NoSig
PAK2	0.464	9.338	1.732	0.084626963	0.133164333	-5.470	NoSig
ITFG1	-0.502	9.535	-1.731	0.084923707	0.133490014	-5.473	NoSig
MMP2	-1.019	9.081	-1.729	0.0852632	0.133882131	-5.476	NoSig
SNRPB2	-0.798	10.385	-1.724	0.086067608	0.135002672	-5.484	NoSig
VSTM4	0.884	4.154	1.716	0.087614821	0.137284762	-5.498	NoSig
CRTAP	-0.548	8.993	-1.712	0.088261739	0.138152848	-5.504	NoSig
GPR37L1	0.895	4.797	1.711	0.088564131	0.138480402	-5.507	NoSig
ARSB	-0.580	7.236	-1.709	0.088913985	0.138881403	-5.510	NoSig
MUC6	1.278	6.272	1.706	0.089390884	0.139479795	-5.514	NoSig
IL12RB1	0.731	5.745	1.704	0.089728899	0.139860453	-5.517	NoSig
GINS4	-0.800	7.457	-1.700	0.090522225	0.140949266	-5.524	NoSig
MAGI1	-0.601	8.302	-1.696	0.091359878	0.142104747	-5.532	NoSig
SCN8A	0.727	3.811	1.694	0.091658935	0.142420936	-5.534	NoSig
TRAPPC2	-0.519	8.094	-1.689	0.092718043	0.143693454	-5.543	NoSig
LPP	-0.537	10.227	-1.688	0.092753317	0.143693454	-5.544	NoSig
PTPN7	-0.520	8.750	-1.688	0.092767798	0.143693454	-5.544	NoSig
SLC4A1AP	0.356	9.429	1.687	0.092992331	0.143891359	-5.546	NoSig
HPRT1	-0.841	10.226	-1.682	0.0939247	0.145182982	-5.554	NoSig
UBR4	0.785	9.429	1.680	0.094334867	0.145665574	-5.557	NoSig

ABCA8	-1.041	5.675	-1.678	0.094814017	0.146253571	-5.561	NoSig
ADAM15	1.161	7.429	1.674	0.095545187	0.147228698	-5.567	NoSig
COCH	-1.354	8.151	-1.673	0.095700425	0.14731525	-5.569	NoSig
GRIK2	-0.608	4.010	-1.669	0.096443713	0.148305896	-5.575	NoSig
ZNF292	-0.599	9.375	-1.665	0.09723978	0.149375571	-5.582	NoSig
LGALS3	-1.040	11.952	-1.663	0.097693148	0.149917143	-5.585	NoSig
PRKD3	-0.645	8.720	-1.662	0.098008063	0.15024535	-5.588	NoSig
ZNF286A	-0.647	7.875	-1.660	0.098382703	0.150664345	-5.591	NoSig
ATXN1	-0.716	8.442	-1.657	0.09888561	0.151278706	-5.595	NoSig
SLC2A11	0.985	6.013	1.654	0.099464641	0.152008141	-5.600	NoSig
P2RY10	-0.931	6.097	-1.648	0.100839447	0.153950983	-5.610	NoSig
RPS6KA2	-0.414	8.696	-1.647	0.101060993	0.154130971	-5.612	NoSig
BCL10	-0.675	8.917	-1.644	0.101593875	0.154784931	-5.616	NoSig
AMN	0.698	7.711	1.641	0.102127224	0.155438262	-5.621	NoSig
TBL3	-0.831	7.545	-1.637	0.102982601	0.156579885	-5.627	NoSig
RSRC1	-0.442	8.895	-1.632	0.104164605	0.158215289	-5.636	NoSig
VIM	0.936	13.257	1.630	0.104464373	0.158455717	-5.638	NoSig
ARMCX5	0.558	7.907	1.630	0.104536018	0.158455717	-5.639	NoSig
CLDN15	-0.811	7.198	-1.629	0.104756462	0.158628165	-5.641	NoSig
SIM1	-1.023	5.979	-1.628	0.10498968	0.158661625	-5.642	NoSig
EPHA7	-1.150	4.594	-1.628	0.104991957	0.158661625	-5.642	NoSig
SLC9A1	0.827	6.240	1.626	0.105465803	0.159215888	-5.646	NoSig
EIF2B5	-0.345	9.290	-1.620	0.106696323	0.160910175	-5.655	NoSig
MCFD2	-0.476	8.983	-1.618	0.107065835	0.161303846	-5.658	NoSig
AFF3	1.202	4.198	1.613	0.108194301	0.16268198	-5.666	NoSig
FRY	0.853	7.077	1.613	0.10819938	0.16268198	-5.666	NoSig
ASXL2	-0.293	9.737	-1.604	0.110144584	0.165439391	-5.680	NoSig
ANKMY1	0.881	5.442	1.603	0.110449244	0.165729593	-5.683	NoSig
PTCH1	-0.736	7.362	-1.601	0.110871337	0.16619524	-5.686	NoSig
MBNL1	0.504	10.930	1.594	0.112453159	0.168243087	-5.697	NoSig
HDGFRP3	0.601	8.339	1.594	0.112463772	0.168243087	-5.697	NoSig
PTPRB	0.883	5.826	1.581	0.115237751	0.172219634	-5.716	NoSig
MAK	0.936	5.240	1.579	0.115720002	0.17276671	-5.719	NoSig
IRAK3	1.068	5.594	1.578	0.115905895	0.172870678	-5.720	NoSig
FCRL2	-0.754	7.053	-1.578	0.116096319	0.172981189	-5.722	NoSig
GAB1	-0.691	7.138	-1.575	0.116769411	0.173809925	-5.726	NoSig
KDELR1	0.734	9.517	1.565	0.119001774	0.17687659	-5.741	NoSig
SP1	1.028	7.067	1.565	0.119067563	0.17687659	-5.742	NoSig
SAR1B	0.565	8.926	1.561	0.119836942	0.177714062	-5.747	NoSig
VPS37C	0.555	9.725	1.561	0.119870346	0.177714062	-5.747	NoSig
DGKE	0.781	6.214	1.560	0.120136835	0.177931747	-5.748	NoSig
BECN1	0.465	9.508	1.551	0.122321488	0.180987118	-5.763	NoSig
SIK3	0.296	8.733	1.545	0.12365987	0.182721978	-5.771	NoSig
HIVEP1	0.447	7.577	1.545	0.123739766	0.182721978	-5.772	NoSig
GSTO1	-0.341	11.578	-1.535	0.126214259	0.186191074	-5.787	NoSig

IST1	0.405	10.684	1.532	0.127013543	0.187184478	-5.792	NoSig
ITPKB	0.871	8.139	1.522	0.129517221	0.190685254	-5.807	NoSig
PER3	-0.650	7.877	-1.518	0.130379076	0.191710374	-5.812	NoSig
PDZRN3	1.003	7.026	1.518	0.13047135	0.191710374	-5.813	NoSig
RPS9	-0.982	12.465	-1.515	0.131194716	0.192582964	-5.817	NoSig
STX16	0.388	10.554	1.511	0.132162607	0.193812423	-5.823	NoSig
DUOX1	-0.836	6.652	-1.510	0.132585336	0.194240783	-5.825	NoSig
FAM189B	1.065	9.116	1.508	0.133025605	0.194693971	-5.828	NoSig
DDAH1	-0.701	10.190	-1.501	0.134696385	0.196945453	-5.837	NoSig
ZBTB24	-0.516	8.855	-1.500	0.135079984	0.197312315	-5.839	NoSig
TMED5	-0.722	9.879	-1.497	0.135864873	0.198264049	-5.844	NoSig
CDKN3	-0.410	9.511	-1.494	0.136717634	0.199242706	-5.849	NoSig
TCEA1	-0.622	11.442	-1.493	0.136803499	0.199242706	-5.849	NoSig
CA8	0.931	6.706	1.487	0.138440472	0.201429532	-5.858	NoSig
TUBGCP3	1.041	7.639	1.483	0.139387605	0.202609353	-5.864	NoSig
ZBTB38	-0.482	9.537	-1.477	0.141106204	0.20474735	-5.873	NoSig
NEK4	0.495	7.637	1.477	0.141133849	0.20474735	-5.873	NoSig
FGD2	-0.743	7.705	-1.471	0.142791016	0.206949552	-5.882	NoSig
TRDN	-1.003	3.184	-1.462	0.145031021	0.209991361	-5.894	NoSig
DHX30	0.271	9.453	1.461	0.145340852	0.210178555	-5.896	NoSig
SPP1	-0.927	12.502	-1.461	0.145442995	0.210178555	-5.896	NoSig
DPY19L4	0.431	8.733	1.456	0.146715399	0.211811455	-5.903	NoSig
MAST4	0.633	7.991	1.454	0.147277616	0.212416891	-5.906	NoSig
IGFBP5	-1.174	10.474	-1.449	0.148801467	0.214406766	-5.914	NoSig
ZSCAN5A	0.580	7.015	1.440	0.151284546	0.217773591	-5.926	NoSig
MBP	-0.555	9.241	-1.439	0.151551012	0.217946185	-5.928	NoSig
ETV4	0.770	5.746	1.430	0.154230318	0.221585008	-5.941	NoSig
CSPP1	-0.510	8.872	-1.427	0.15504694	0.222543242	-5.945	NoSig
TAF11	0.383	9.263	1.421	0.156797795	0.224839269	-5.953	NoSig
CACNG5	-0.784	5.517	-1.414	0.158642749	0.227265672	-5.962	NoSig
GPNMB	-0.749	11.785	-1.409	0.160307188	0.229429055	-5.970	NoSig
BANK1	1.071	6.118	1.403	0.162132096	0.231817719	-5.979	NoSig
STEAP3	0.387	9.892	1.394	0.164732948	0.235310176	-5.991	NoSig
CEP70	-0.442	8.617	-1.392	0.165225838	0.23544075	-5.993	NoSig
ZNF41	0.917	5.093	1.392	0.165282116	0.23544075	-5.993	NoSig
ABCC5	0.489	9.622	1.392	0.165299357	0.23544075	-5.993	NoSig
ACAT1	0.464	10.235	1.391	0.165648824	0.235712728	-5.995	NoSig
PPIP5K1	0.698	6.359	1.381	0.16874795	0.239893118	-6.009	NoSig
RAP1A	-0.694	9.689	-1.376	0.170357317	0.241949694	-6.016	NoSig
CAD	0.424	9.258	1.373	0.171008916	0.242643376	-6.019	NoSig
TBC1D16	0.505	9.071	1.369	0.172361653	0.244329626	-6.024	NoSig
CTBP1	0.343	10.800	1.365	0.173588267	0.24583405	-6.030	NoSig
HEATR1	-0.320	10.298	-1.360	0.175065544	0.24769026	-6.036	NoSig
ZNF107	0.606	7.911	1.358	0.175773813	0.248455951	-6.039	NoSig
GTPBP3	-0.328	9.653	-1.352	0.17765687	0.25087917	-6.047	NoSig

TAGLN	1.123	10.038	1.348	0.178924174	0.252429076	-6.052	NoSig
NLGN4Y	-0.839	4.778	-1.342	0.18105068	0.255187073	-6.061	NoSig
LSM14B	0.871	6.679	1.341	0.181343618	0.255357916	-6.062	NoSig
RNF141	0.508	7.946	1.340	0.181654611	0.255553837	-6.064	NoSig
SERPINC1	0.534	4.050	1.339	0.181908466	0.255669083	-6.065	NoSig
SORBS2	1.053	8.350	1.337	0.182586855	0.25638022	-6.067	NoSig
KRT7	-0.901	10.825	-1.329	0.185292773	0.259934296	-6.078	NoSig
IQCA1	0.532	7.525	1.327	0.185788546	0.260384136	-6.080	NoSig
SRCAP	0.445	7.925	1.323	0.187101617	0.261738069	-6.085	NoSig
SQLE	-0.525	10.396	-1.323	0.187106636	0.261738069	-6.085	NoSig
C2orf72	-0.610	7.060	-1.319	0.188463075	0.263387776	-6.091	NoSig
EHBP1L1	0.475	7.894	1.318	0.188843951	0.263672258	-6.092	NoSig
CKAP5	-0.379	10.041	-1.312	0.190713055	0.266032188	-6.099	NoSig
ZHX2	-0.578	8.796	-1.310	0.191682066	0.267133301	-6.103	NoSig
SDC3	-0.699	8.324	-1.309	0.191950322	0.267256675	-6.104	NoSig
CRABP1	1.246	8.815	1.306	0.193028656	0.268506653	-6.108	NoSig
ENAH	0.487	10.634	1.302	0.194333855	0.270069572	-6.113	NoSig
ATF1	-0.668	8.049	-1.290	0.198345895	0.275387811	-6.128	NoSig
PLXND1	0.468	9.906	1.289	0.198769499	0.275409742	-6.130	NoSig
SRGN	-0.764	11.284	-1.289	0.198870102	0.275409742	-6.130	NoSig
WDR37	0.308	8.989	1.289	0.198917326	0.275409742	-6.130	NoSig
NEUROD1	-0.728	4.745	-1.285	0.200282441	0.277041851	-6.135	NoSig
RAG1	0.744	5.029	1.274	0.204109394	0.282073112	-6.149	NoSig
ALB	0.826	5.800	1.273	0.204404077	0.28221807	-6.150	NoSig
LRRK1	0.490	7.372	1.262	0.208184482	0.287170988	-6.163	NoSig
MOB3B	0.368	8.711	1.261	0.208597328	0.287473797	-6.165	NoSig
SERPINB5	-1.092	8.211	-1.258	0.209860486	0.288946799	-6.169	NoSig
MAP3K11	-0.495	9.313	-1.257	0.210243215	0.289205977	-6.170	NoSig
SNED1	-0.491	7.534	-1.255	0.210981148	0.289952835	-6.173	NoSig
PHC3	0.481	7.215	1.253	0.211438331	0.290312833	-6.175	NoSig
FAM188A	0.552	8.024	1.250	0.212697069	0.291771717	-6.179	NoSig
NFS1	0.282	8.906	1.246	0.213901239	0.293153127	-6.183	NoSig
RUFY2	0.648	6.598	1.239	0.216589802	0.29656449	-6.192	NoSig
PHTF2	-0.370	8.759	-1.236	0.217671344	0.297771194	-6.195	NoSig
LTBR	0.344	9.218	1.231	0.219638858	0.300186564	-6.202	NoSig
NBR2	0.833	5.279	1.230	0.220063755	0.300491096	-6.203	NoSig
EIF4G3	0.379	8.789	1.227	0.221019983	0.301519922	-6.206	NoSig
TFE3	-0.519	8.516	-1.226	0.221389968	0.301747829	-6.208	NoSig
PRR5L	0.660	6.423	1.220	0.223756178	0.304325556	-6.215	NoSig
KIFC3	-0.431	7.749	-1.220	0.223779799	0.304325556	-6.215	NoSig
C3orf52	0.630	6.628	1.220	0.223895198	0.304325556	-6.216	NoSig
UBA6	-0.411	8.062	-1.219	0.224203324	0.304466066	-6.217	NoSig
NCOA6	0.283	9.806	1.217	0.225024634	0.305302583	-6.219	NoSig
MPP6	0.852	6.017	1.214	0.226041568	0.306402746	-6.222	NoSig
PRUNE2	-0.446	7.783	-1.208	0.228391226	0.309305786	-6.230	NoSig

RNASET2	-0.581	11.060	-1.196	0.232891237	0.315113074	-6.244	NoSig
TACC1	-0.421	10.513	-1.192	0.23450242	0.316964372	-6.248	NoSig
CMTM6	0.782	10.561	1.192	0.234685793	0.316964372	-6.249	NoSig
LPGAT1	-0.408	9.560	-1.191	0.235050105	0.317168336	-6.250	NoSig
TGIF1	-0.344	9.232	-1.189	0.235788751	0.317876584	-6.252	NoSig
SH2D3C	-0.354	7.402	-1.183	0.238094298	0.320463599	-6.259	NoSig
CNPY3	0.713	7.960	1.183	0.23813872	0.320463599	-6.259	NoSig
NAV3	0.535	5.719	1.180	0.239331885	0.32177804	-6.263	NoSig
EIF4G2	-0.621	12.143	-1.176	0.24091472	0.32361354	-6.267	NoSig
ZMAT3	-0.357	8.005	-1.175	0.241225982	0.323715251	-6.268	NoSig
ARMCX4	-0.525	4.664	-1.174	0.24183996	0.323715251	-6.270	NoSig
PTPN11	0.475	10.243	1.173	0.242001204	0.323715251	-6.271	NoSig
RBMS3	0.506	7.757	1.173	0.242002798	0.323715251	-6.271	NoSig
OSBPL1A	-0.344	9.489	-1.173	0.242078923	0.323715251	-6.271	NoSig
GINS2	0.850	8.198	1.172	0.242319057	0.323745227	-6.272	NoSig
PSMD6	-0.365	10.776	-1.172	0.24254852	0.323760906	-6.272	NoSig
NKTR	-0.353	10.307	-1.170	0.243298406	0.324470609	-6.274	NoSig
DENND4A	0.830	5.794	1.166	0.244812914	0.326197852	-6.279	NoSig
ABI3BP	0.730	5.652	1.165	0.245082374	0.32626454	-6.279	NoSig
NDST2	0.331	8.348	1.164	0.24585789	0.327004189	-6.282	NoSig
FBN2	-0.614	7.201	-1.160	0.247389517	0.328747285	-6.286	NoSig
TXNL4A	0.375	11.289	1.155	0.249262804	0.330940883	-6.291	NoSig
VNN1	0.835	5.882	1.154	0.24956447	0.331045823	-6.292	NoSig
THADA	0.280	9.102	1.151	0.25093841	0.332571672	-6.296	NoSig
PAAF1	0.617	7.858	1.147	0.252552621	0.334412954	-6.300	NoSig
TNRC6B	0.425	8.004	1.143	0.254159303	0.336241	-6.305	NoSig
GNG4	-0.542	8.324	-1.142	0.25475597	0.33673078	-6.306	NoSig
BTG3	0.536	10.019	1.138	0.25623332	0.338382723	-6.310	NoSig
CLU	1.063	10.016	1.132	0.258888608	0.341585945	-6.318	NoSig
TSPAN3	-0.390	10.262	-1.128	0.260622126	0.343568352	-6.322	NoSig
CCDC69	-0.816	6.459	-1.124	0.262285647	0.345455055	-6.327	NoSig
ITGA9	0.494	7.058	1.120	0.263886079	0.347255398	-6.331	NoSig
NSMAF	0.300	9.564	1.118	0.264578877	0.34773339	-6.333	NoSig
PDZK1IP1	0.637	9.814	1.118	0.264717013	0.34773339	-6.333	NoSig
TMBIM4	-0.327	9.832	-1.107	0.269707755	0.353798223	-6.346	NoSig
TXNDC9	-0.258	10.426	-1.106	0.269809808	0.353798223	-6.346	NoSig
KIAA1107	-0.358	6.869	-1.106	0.270049057	0.353799954	-6.347	NoSig
C11orf63	0.555	6.153	1.098	0.273368713	0.35783387	-6.355	NoSig
SRRM2	0.424	11.802	1.094	0.275231266	0.35995505	-6.360	NoSig
ACOT11	0.497	6.911	1.093	0.27560598	0.360128376	-6.361	NoSig
CLEC2B	-0.582	9.262	-1.091	0.276668056	0.361198771	-6.363	NoSig
GPRIN2	0.361	8.924	1.089	0.277413186	0.361853866	-6.365	NoSig
FCER1A	0.576	6.200	1.087	0.278063384	0.362384095	-6.367	NoSig
HSPA6	0.530	9.429	1.083	0.280046994	0.364649633	-6.371	NoSig
MACROD1	-0.512	8.328	-1.075	0.283417024	0.368714886	-6.379	NoSig

MCTS1	0.350	9.930	1.074	0.283929716	0.369058993	-6.381	NoSig
OTUB2	-0.500	5.232	-1.064	0.288354664	0.374483307	-6.391	NoSig
CLCC1	0.392	9.307	1.063	0.288813549	0.374599292	-6.392	NoSig
CCL5	-0.556	10.034	-1.063	0.288947806	0.374599292	-6.393	NoSig
ZNF12	0.409	8.696	1.061	0.289979688	0.375609578	-6.395	NoSig
TTLL4	-0.289	8.508	-1.059	0.29057354	0.376051221	-6.396	NoSig
LRP1	-0.341	7.700	-1.057	0.291664367	0.377134708	-6.399	NoSig
RBPMS	-0.578	8.908	-1.049	0.295398638	0.381631428	-6.407	NoSig
POF1B	-0.740	5.274	-1.043	0.298134194	0.384700115	-6.413	NoSig
TTC37	0.271	8.371	1.043	0.298291347	0.384700115	-6.414	NoSig
DGKB	-0.726	4.019	-1.041	0.299011117	0.385019044	-6.415	NoSig
WWC1	-0.298	9.228	-1.041	0.299242023	0.385019044	-6.416	NoSig
ATAD2B	0.362	8.364	1.040	0.29931541	0.385019044	-6.416	NoSig
METTL17	-0.490	9.303	-1.033	0.302552804	0.388847035	-6.423	NoSig
ZBED1	-0.325	9.030	-1.032	0.30306061	0.389163322	-6.424	NoSig
RRP12	0.433	7.271	1.030	0.304027709	0.390068338	-6.426	NoSig
NLRP1	0.480	6.800	1.026	0.306067224	0.39180306	-6.431	NoSig
FAM53B	0.245	9.720	1.026	0.306097846	0.39180306	-6.431	NoSig
ST8SIA4	-0.521	6.972	-1.026	0.306170246	0.39180306	-6.431	NoSig
BCL2L13	0.261	8.867	1.020	0.308669476	0.39466166	-6.437	NoSig
SLC12A1	-0.858	4.894	-1.015	0.311267359	0.39742835	-6.442	NoSig
ATP5H	0.373	12.044	1.014	0.311477416	0.39742835	-6.442	NoSig
ASCC1	-0.351	8.839	-1.014	0.311635142	0.39742835	-6.443	NoSig
SOBP	-0.509	7.800	-1.010	0.313464534	0.399418819	-6.447	NoSig
SLAMF1	0.360	7.805	1.006	0.315415463	0.401560611	-6.451	NoSig
TEX10	0.205	9.679	1.005	0.31594867	0.401895357	-6.452	NoSig
TNNT2	0.524	5.980	1.003	0.31719501	0.40313588	-6.454	NoSig
ACAP2	-0.369	9.093	-0.999	0.318751125	0.404767654	-6.458	NoSig
MCF2L	-0.308	9.374	-0.999	0.319103785	0.404869734	-6.458	NoSig
UHRF1BP1L	0.584	6.920	0.997	0.319787052	0.405206903	-6.460	NoSig
FILIP1L	0.437	8.369	0.996	0.320148788	0.405206903	-6.460	NoSig
IBA57	0.552	6.114	0.996	0.320422376	0.405206903	-6.461	NoSig
BTG1	-0.373	10.968	-0.996	0.320459528	0.405206903	-6.461	NoSig
NFYC	0.306	9.110	0.994	0.321413681	0.406068092	-6.463	NoSig
ATP5S	0.228	8.314	0.991	0.322565128	0.407176864	-6.465	NoSig
HS3ST3B1	0.479	6.818	0.983	0.326917875	0.412321358	-6.474	NoSig
CNOT7	-0.298	10.799	-0.974	0.331110348	0.417255159	-6.482	NoSig
RALGPS1	0.412	6.325	0.968	0.334020715	0.420555358	-6.488	NoSig
TPM1	0.458	11.448	0.968	0.334316538	0.420555358	-6.489	NoSig
MCOLN3	-0.615	5.738	-0.967	0.334577666	0.420555358	-6.489	NoSig
IREB2	0.360	7.079	0.966	0.334872401	0.420570321	-6.490	NoSig
SLC16A1	-0.381	9.232	-0.963	0.336514704	0.422276258	-6.493	NoSig
SPAG16	0.468	7.196	0.958	0.339024039	0.425066397	-6.497	NoSig
DDX42	0.277	10.405	0.954	0.341359873	0.427296319	-6.502	NoSig
BRD3	-0.328	9.762	-0.954	0.341377288	0.427296319	-6.502	NoSig

H6PD	-0.414	7.497	-0.947	0.344528072	0.430284427	-6.508	NoSig
ATF3	0.313	9.283	0.947	0.344623839	0.430284427	-6.508	NoSig
TIMM17B	-0.485	8.876	-0.947	0.344632651	0.430284427	-6.508	NoSig
SLC23A2	-0.193	7.715	-0.946	0.344938957	0.430305561	-6.509	NoSig
FKBP4	-0.369	10.697	-0.940	0.348388213	0.434244152	-6.515	NoSig
AGFG2	-0.342	7.627	-0.938	0.349114057	0.434784424	-6.516	NoSig
CFI	0.497	8.202	0.935	0.350628749	0.436305397	-6.519	NoSig
NAMPT	-0.425	10.415	-0.929	0.353760175	0.439769713	-6.525	NoSig
CHERP	0.345	9.729	0.929	0.354004268	0.439769713	-6.525	NoSig
NT5DC2	-0.494	9.004	-0.928	0.354571035	0.440106118	-6.526	NoSig
GGA1	0.392	11.256	0.927	0.355071688	0.440359966	-6.527	NoSig
UBE2I	-0.297	11.091	-0.922	0.357697176	0.442934401	-6.531	NoSig
TTC26	-0.473	6.468	-0.922	0.357743252	0.442934401	-6.532	NoSig
PTPRC	-0.522	10.049	-0.920	0.358677894	0.443722153	-6.533	NoSig
FAM184A	0.344	8.099	0.918	0.359374043	0.4442138	-6.534	NoSig
RPS24	-0.387	14.428	-0.907	0.36517343	0.451007384	-6.544	NoSig
UEVLD	-0.317	7.518	-0.905	0.366318833	0.451930215	-6.546	NoSig
INTS6	0.364	8.430	0.905	0.366528473	0.451930215	-6.547	NoSig
CRYBB2P1	0.490	6.756	0.901	0.368674045	0.454199093	-6.550	NoSig
C11orf58	-0.371	10.933	-0.900	0.369035365	0.454267871	-6.551	NoSig
PCDH9	-0.510	6.125	-0.893	0.372597533	0.458273393	-6.557	NoSig
WDR3	0.424	9.059	0.890	0.374184482	0.459844896	-6.560	NoSig
TTF2	0.381	6.987	0.884	0.377657905	0.463730227	-6.565	NoSig
KCNK1	0.397	9.414	0.877	0.381479508	0.468036328	-6.571	NoSig
G6PC	-0.602	3.434	-0.875	0.38244761	0.46883726	-6.573	NoSig
GGNBP2	-0.309	9.356	-0.872	0.384006227	0.469972416	-6.575	NoSig
TP63	-0.410	7.878	-0.872	0.384286193	0.469972416	-6.576	NoSig
DENND1B	0.272	8.361	0.872	0.384321761	0.469972416	-6.576	NoSig
C18orf25	0.264	7.893	0.869	0.385835744	0.471436114	-6.578	NoSig
WDR62	0.332	7.124	0.865	0.387753022	0.473389773	-6.581	NoSig
NFE2L2	-0.256	10.217	-0.864	0.388496878	0.473908825	-6.583	NoSig
CEP164	-0.318	8.782	-0.861	0.390204291	0.475601459	-6.585	NoSig
CYP39A1	0.643	5.844	0.856	0.392986728	0.478600544	-6.589	NoSig
ELAVL3	-0.324	4.808	-0.854	0.393911074	0.479333688	-6.591	NoSig
SYNRG	-0.349	8.411	-0.853	0.394416915	0.479556788	-6.592	NoSig
CAND1	-0.335	8.954	-0.847	0.397752875	0.48321775	-6.597	NoSig
C16orf45	0.371	7.277	0.844	0.399345728	0.484756814	-6.599	NoSig
TMEM135	-0.441	7.445	-0.841	0.401243759	0.486663515	-6.602	NoSig
PIKFYVE	0.374	7.932	0.839	0.402530438	0.487826211	-6.604	NoSig
PAK6	0.322	7.752	0.834	0.405338517	0.490605351	-6.608	NoSig
CDKL3	0.485	6.465	0.833	0.405483508	0.490605351	-6.608	NoSig
ADCY7	0.359	8.082	0.828	0.408555874	0.4939208	-6.613	NoSig
HOPX	0.458	7.998	0.827	0.409249306	0.494357204	-6.614	NoSig
RBM25	-0.223	9.991	-0.823	0.411366321	0.496511136	-6.617	NoSig
IPP	-0.278	7.920	-0.820	0.413324555	0.498470084	-6.620	NoSig

FGFR1OP	-0.283	8.930	-0.818	0.414281826	0.499219672	-6.621	NoSig
ITGBL1	0.353	7.733	0.816	0.415101986	0.49953993	-6.622	NoSig
GOLGA7	-0.204	10.999	-0.816	0.415219471	0.49953993	-6.622	NoSig
MTUS2	0.460	4.039	0.816	0.415624656	0.499623172	-6.623	NoSig
CEL	0.541	4.429	0.812	0.417410889	0.501365099	-6.625	NoSig
USPL1	0.185	8.529	0.805	0.42167721	0.50608072	-6.631	NoSig
COBL	-0.613	8.214	-0.798	0.425590134	0.510290604	-6.637	NoSig
IQCG	-0.321	9.138	-0.798	0.425871311	0.510290604	-6.637	NoSig
EPB41L4A	0.384	5.585	0.794	0.428147705	0.512385289	-6.640	NoSig
WDR19	-0.283	8.143	-0.793	0.428385353	0.512385289	-6.641	NoSig
PDS5B	0.243	8.846	0.793	0.428653194	0.512385289	-6.641	NoSig
TRMT61A	-0.285	8.272	-0.792	0.429399216	0.512502117	-6.642	NoSig
TMEM144	-0.408	5.487	-0.792	0.42944024	0.512502117	-6.642	NoSig
CBR4	0.259	8.246	0.787	0.432251665	0.515344074	-6.646	NoSig
DNAJB9	-0.179	10.294	-0.786	0.432554557	0.515344074	-6.646	NoSig
DET1	0.176	8.252	0.786	0.432969359	0.515344074	-6.647	NoSig
CD79B	0.460	6.527	0.785	0.433207863	0.515344074	-6.647	NoSig
NLGN4X	-0.600	5.254	-0.784	0.433997043	0.515870186	-6.648	NoSig
PTPRS	0.475	5.312	0.782	0.435032167	0.516687566	-6.650	NoSig
CHRNA10	-0.222	7.159	-0.780	0.436245853	0.517715549	-6.651	NoSig
NAA16	-0.213	8.284	-0.777	0.438059008	0.519452747	-6.654	NoSig
MKL2	0.168	9.387	0.776	0.438466453	0.519521606	-6.654	NoSig
SSBP2	0.232	7.956	0.774	0.439572181	0.520417065	-6.655	NoSig
SMTN	0.365	8.333	0.773	0.440073716	0.520596353	-6.656	NoSig
ALG5	0.410	9.823	0.761	0.44732173	0.52874993	-6.665	NoSig
MRPS27	-0.246	8.933	-0.751	0.453597842	0.535607937	-6.673	NoSig
SMOX	0.362	8.078	0.750	0.453843981	0.535607937	-6.674	NoSig
CMAHP	-0.306	7.783	-0.749	0.454388865	0.535680919	-6.674	NoSig
PTPRE	0.290	8.657	0.749	0.454626308	0.535680919	-6.675	NoSig
LPAR6	-0.316	8.748	-0.747	0.456153201	0.537054482	-6.676	NoSig
ELMO2	0.199	7.927	0.743	0.45823107	0.539074051	-6.679	NoSig
ALOX15B	-0.417	7.475	-0.740	0.459961385	0.540681881	-6.681	NoSig
PRMT1	-0.351	10.657	-0.731	0.465327814	0.546158083	-6.688	NoSig
ATP6V1H	-0.225	9.835	-0.731	0.465354601	0.546158083	-6.688	NoSig
CCDC132	0.418	6.484	0.730	0.466016089	0.546503095	-6.688	NoSig
TFAP2B	0.800	6.221	0.725	0.469450373	0.549783019	-6.692	NoSig
INPP5B	-0.375	6.879	-0.723	0.470330077	0.549783019	-6.694	NoSig
RGS12	0.185	8.074	0.723	0.470419312	0.549783019	-6.694	NoSig
PRPSAP1	0.181	10.140	0.723	0.470741995	0.549783019	-6.694	NoSig
PSEN1	-0.164	9.433	-0.722	0.471115594	0.549783019	-6.694	NoSig
TPM4	0.403	11.394	0.722	0.471119596	0.549783019	-6.694	NoSig
STRAP	0.223	11.745	0.721	0.471401042	0.549783019	-6.695	NoSig
RSU1	-0.318	10.193	-0.720	0.472434384	0.550215123	-6.696	NoSig
TUBA1B	0.565	13.659	0.720	0.472511575	0.550215123	-6.696	NoSig
RAF1	-0.144	10.276	-0.719	0.47298395	0.55033422	-6.697	NoSig

KLHL35	-0.303	7.351	-0.718	0.473518978	0.550525974	-6.697	NoSig
EPHB3	0.390	9.699	0.717	0.473997284	0.550651532	-6.698	NoSig
PIK3IP1	-0.301	8.660	-0.716	0.475005292	0.551391779	-6.699	NoSig
KCNMB1	-0.269	8.752	-0.714	0.476199829	0.552347227	-6.700	NoSig
RPAP3	-0.260	8.232	-0.701	0.484201641	0.561190834	-6.709	NoSig
ZNF230	0.375	6.000	0.698	0.485927289	0.562752242	-6.711	NoSig
TOR1A	-0.287	8.732	-0.691	0.49009	0.567131385	-6.716	NoSig
ZNF26	-0.187	8.625	-0.688	0.492004006	0.568903544	-6.718	NoSig
ERGIC3	-0.387	10.543	-0.684	0.494438838	0.571274711	-6.721	NoSig
PDE12	0.453	6.481	0.682	0.495704648	0.572292556	-6.722	NoSig
RAD51B	-0.242	6.620	-0.681	0.496571345	0.572848402	-6.723	NoSig
DNAJC7	0.201	9.482	0.677	0.499040371	0.575250412	-6.726	NoSig
ANXA2	0.327	13.283	0.676	0.499508602	0.575344145	-6.726	NoSig
CDH19	0.512	6.271	0.674	0.500923302	0.576527051	-6.728	NoSig
HSD17B4	0.244	9.490	0.670	0.50371401	0.57929059	-6.730	NoSig
PEX2	0.200	9.250	0.665	0.506570763	0.582125753	-6.733	NoSig
PTK7	-0.308	8.727	-0.658	0.510930746	0.58668264	-6.738	NoSig
FCGR2A	-0.305	9.174	-0.649	0.517023718	0.593220886	-6.744	NoSig
EMID1	-0.385	7.362	-0.645	0.519277288	0.595347206	-6.746	NoSig
SLC22A7	-0.322	6.208	-0.644	0.520010341	0.595728334	-6.747	NoSig
SLC29A2	-0.318	7.003	-0.644	0.52048467	0.595812705	-6.748	NoSig
CLDN11	0.258	7.484	0.638	0.524286326	0.599702898	-6.751	NoSig
GIGYF2	0.129	9.578	0.635	0.525790389	0.600961036	-6.753	NoSig
JRKL	-0.293	7.803	-0.629	0.529741642	0.605012152	-6.757	NoSig
ST6GALNAC5	0.272	7.777	0.627	0.531002538	0.60598678	-6.758	NoSig
CAPRIN2	0.196	8.764	0.626	0.532174945	0.606859006	-6.759	NoSig
HS1BP3	0.210	9.032	0.625	0.532891953	0.607210984	-6.760	NoSig
GCLC	-0.208	8.776	-0.621	0.534987199	0.609131673	-6.761	NoSig
HEXA	-0.229	9.617	-0.619	0.536438653	0.610316968	-6.763	NoSig
RPS15	-0.373	13.631	-0.595	0.552747586	0.628391178	-6.778	NoSig
TAPBP	0.217	11.091	0.593	0.553773396	0.629076425	-6.779	NoSig
AKAP12	-0.364	7.014	-0.589	0.55655753	0.631461315	-6.781	NoSig
LLGL2	-0.179	8.857	-0.589	0.556722114	0.631461315	-6.781	NoSig
PARP11	0.334	6.273	0.582	0.561267612	0.636131813	-6.785	NoSig
C5orf28	0.219	7.673	0.580	0.562814632	0.637399359	-6.786	NoSig
KIAA1109	0.203	8.391	0.578	0.563801847	0.63756229	-6.787	NoSig
FGFR4	-0.350	6.200	-0.578	0.563816013	0.63756229	-6.787	NoSig
GTF3C3	0.176	8.636	0.576	0.564891951	0.638293565	-6.788	NoSig
RBL1	0.217	6.789	0.574	0.566559504	0.639691711	-6.790	NoSig
SFXN3	0.166	8.190	0.556	0.5789261	0.653158657	-6.800	NoSig
PABPC1	-0.278	14.264	-0.551	0.582001337	0.656130393	-6.802	NoSig
KLHL3	0.241	7.204	0.549	0.58356304	0.657392607	-6.804	NoSig
MEIS1	0.192	7.573	0.544	0.587092965	0.660868463	-6.806	NoSig
NAALAD2	-0.377	3.919	-0.543	0.587677563	0.661026124	-6.807	NoSig
OSBP	-0.124	9.396	-0.541	0.588790012	0.661776831	-6.808	NoSig

PIGG	0.129	9.342	0.538	0.59129634	0.664091886	-6.810	NoSig
G3BP1	-0.167	10.222	-0.532	0.595573552	0.668390846	-6.813	NoSig
SEMA6A	-0.136	7.921	-0.526	0.599166757	0.671193486	-6.816	NoSig
MCTP2	0.167	7.744	0.526	0.599193766	0.671193486	-6.816	NoSig
ECM2	0.189	7.689	0.526	0.599424983	0.671193486	-6.816	NoSig
MYCBP2	0.186	9.790	0.524	0.600603643	0.672007237	-6.817	NoSig
PIEZO1	0.282	9.417	0.520	0.603528836	0.674772466	-6.819	NoSig
PALLD	0.229	10.842	0.513	0.608320636	0.679618922	-6.823	NoSig
KLK2	-0.243	5.925	-0.512	0.60890082	0.679756396	-6.823	NoSig
CENPI	-0.206	6.643	-0.510	0.610317758	0.680607063	-6.824	NoSig
CTNNB1	-0.170	11.149	-0.510	0.610578226	0.680607063	-6.824	NoSig
DNAJA4	0.147	9.593	0.508	0.611718683	0.681367552	-6.825	NoSig
PGD	0.289	9.173	0.506	0.613272986	0.682587522	-6.826	NoSig
TSGA10	0.250	6.474	0.501	0.616544939	0.685716025	-6.828	NoSig
HEMK1	-0.127	8.106	-0.500	0.617636859	0.686417047	-6.829	NoSig
SMCHD1	0.188	9.364	0.497	0.61952536	0.688001651	-6.831	NoSig
EEF1A1	0.364	14.942	0.493	0.622753099	0.691070044	-6.833	NoSig
PDE1A	-0.236	6.238	-0.491	0.624049054	0.691991755	-6.834	NoSig
STAU2	-0.129	8.266	-0.489	0.625222618	0.692776477	-6.834	NoSig
CBX3	0.115	11.968	0.486	0.627476658	0.69449506	-6.836	NoSig
DPYD	0.204	7.640	0.486	0.627707707	0.69449506	-6.836	NoSig
MORN1	0.256	4.596	0.483	0.629708393	0.696190618	-6.838	NoSig
GTF2H5	-0.154	9.767	-0.480	0.632041576	0.698250983	-6.839	NoSig
FN1	-0.313	12.424	-0.478	0.633210567	0.699023098	-6.840	NoSig
MTHFSD	-0.198	7.819	-0.474	0.635651953	0.700986518	-6.842	NoSig
DAAM1	0.210	8.664	0.474	0.635931952	0.700986518	-6.842	NoSig
FAR2	-0.366	7.174	-0.471	0.637963678	0.702705177	-6.843	NoSig
DIXDC1	0.260	6.543	0.469	0.639266473	0.703618982	-6.844	NoSig
DBF4B	0.210	6.663	0.467	0.641005136	0.705010826	-6.845	NoSig
DPY19L1	0.202	7.600	0.464	0.643073183	0.706454963	-6.846	NoSig
CYP4B1	-0.367	5.299	-0.464	0.643268339	0.706454963	-6.847	NoSig
GIN1	0.122	7.714	0.458	0.647541261	0.710622771	-6.849	NoSig
ACAN	-0.267	8.315	-0.454	0.649915302	0.712616275	-6.851	NoSig
SAMSN1	0.225	8.064	0.454	0.650316264	0.712616275	-6.851	NoSig
CLSPN	0.254	5.779	0.449	0.653850271	0.715961231	-6.853	NoSig
CD6	0.222	8.628	0.447	0.655452329	0.717187353	-6.854	NoSig
GPR126	-0.191	8.151	-0.443	0.65820677	0.719671667	-6.856	NoSig
DOPEY1	-0.106	8.341	-0.435	0.663749061	0.725198276	-6.859	NoSig
KDSR	0.104	9.494	0.431	0.666623384	0.727803945	-6.861	NoSig
RFX4	-0.238	3.620	-0.428	0.668906215	0.729760486	-6.862	NoSig
WFDC2	0.240	8.970	0.420	0.674709601	0.735552182	-6.866	NoSig
SLC4A8	0.269	4.666	0.418	0.676262669	0.736705193	-6.867	NoSig
SUPT6H	-0.090	9.463	-0.416	0.677764511	0.737800752	-6.867	NoSig
FRAS1	-0.178	7.326	-0.412	0.680688677	0.74044189	-6.869	NoSig
SERPINE1	0.233	5.943	0.408	0.683525369	0.742984082	-6.871	NoSig

CD44	-0.163	11.948	-0.401	0.688903561	0.748283122	-6.874	NoSig
MRPL23	0.167	9.087	0.395	0.693164336	0.752361582	-6.876	NoSig
DNASE1L3	-0.132	7.032	-0.388	0.698506427	0.757606898	-6.879	NoSig
CELF1	-0.092	11.038	-0.375	0.707955335	0.766830557	-6.884	NoSig
MYNN	0.087	8.079	0.375	0.708041933	0.766830557	-6.884	NoSig
MAD2L1	0.160	9.093	0.372	0.710108271	0.768034164	-6.885	NoSig
SPG11	0.134	8.942	0.372	0.710186264	0.768034164	-6.885	NoSig
P4HB	-0.115	12.989	-0.360	0.719425624	0.777460685	-6.889	NoSig
GATM	0.181	7.522	0.357	0.721436974	0.779068105	-6.890	NoSig
PTGS2	0.255	6.092	0.354	0.723843963	0.781100126	-6.891	NoSig
KDM4C	0.074	8.960	0.352	0.725178487	0.781972741	-6.892	NoSig
PPP2R5C	0.100	9.831	0.344	0.731477898	0.788193938	-6.895	NoSig
TRIM52	0.139	7.850	0.339	0.734585236	0.790969041	-6.896	NoSig
PHF21A	-0.151	8.159	-0.326	0.744960931	0.801347629	-6.901	NoSig
CYB5R1	0.120	9.843	0.325	0.745301796	0.801347629	-6.901	NoSig
TLR4	0.110	8.215	0.323	0.746929115	0.802517048	-6.902	NoSig
DNAJA3	0.060	10.468	0.317	0.751818795	0.806682842	-6.904	NoSig
MYO10	0.132	9.884	0.315	0.75274145	0.806682842	-6.904	NoSig
CD96	0.136	6.886	0.315	0.752814324	0.806682842	-6.904	NoSig
STIM1	-0.068	8.280	-0.315	0.752976318	0.806682842	-6.904	NoSig
UBE2G2	0.166	9.845	0.314	0.753648931	0.806822146	-6.904	NoSig
C7orf63	0.192	5.686	0.311	0.755944421	0.808697377	-6.905	NoSig
CEP41	0.129	7.355	0.307	0.75917237	0.811453427	-6.907	NoSig
TSPYL1	0.094	9.632	0.306	0.759612085	0.811453427	-6.907	NoSig
PDHA1	-0.107	11.248	-0.302	0.763090137	0.814583657	-6.908	NoSig
CKAP2	0.118	8.853	0.300	0.764574996	0.815536856	-6.909	NoSig
CAST	0.078	10.182	0.299	0.765079969	0.815536856	-6.909	NoSig
C16orf62	-0.099	7.944	-0.298	0.765652563	0.815562579	-6.909	NoSig
GRK5	0.129	8.382	0.295	0.768307744	0.817805021	-6.910	NoSig
FAM13C	-0.218	3.575	-0.290	0.771790232	0.820464807	-6.912	NoSig
KLF11	-0.082	8.666	-0.290	0.771910064	0.820464807	-6.912	NoSig
PGK1	0.110	12.543	0.287	0.774716646	0.822380419	-6.913	NoSig
ANKRD28	0.087	8.618	0.286	0.774818404	0.822380419	-6.913	NoSig
CYP11B1	0.121	7.259	0.278	0.781507733	0.82888873	-6.915	NoSig
FXYD2	0.134	6.557	0.276	0.78274848	0.829612965	-6.916	NoSig
CCDC90B	0.066	9.591	0.274	0.784688725	0.831077019	-6.916	NoSig
CLTA	0.098	11.698	0.272	0.785711697	0.831568181	-6.917	NoSig
CYTH2	-0.081	8.864	-0.264	0.792081542	0.837713551	-6.919	NoSig
ZNF277	-0.086	8.558	-0.262	0.793274842	0.83837931	-6.919	NoSig
HRH3	-0.096	5.419	-0.260	0.79490083	0.839501089	-6.920	NoSig
ALDH1A3	0.224	7.689	0.255	0.799221884	0.843465537	-6.921	NoSig
ITIH5	0.125	7.527	0.253	0.800498665	0.84421384	-6.922	NoSig
AFF4	-0.101	7.506	-0.249	0.803528182	0.846808226	-6.923	NoSig
HNMT	-0.111	8.630	-0.246	0.805954813	0.848764028	-6.923	NoSig
DNAJC13	0.087	8.800	0.244	0.807403373	0.849687768	-6.924	NoSig

ARHGDIB	-0.120	11.803	-0.243	0.808265574	0.84999357	-6.924	NoSig
ATP11B	0.099	8.786	0.236	0.813705267	0.855109352	-6.926	NoSig
CASS4	-0.131	4.556	-0.232	0.816388678	0.857323421	-6.927	NoSig
RPRD1A	0.081	8.787	0.225	0.822466442	0.862906286	-6.928	NoSig
THSD4	0.104	6.868	0.224	0.822865577	0.862906286	-6.929	NoSig
EFCAB6	0.099	4.314	0.222	0.824630477	0.864147653	-6.929	NoSig
ITGB2	-0.096	10.455	-0.220	0.825701265	0.864660409	-6.929	NoSig
LEPROT	0.131	9.973	0.215	0.82995839	0.868506774	-6.931	NoSig
ZFC3H1	-0.036	9.350	-0.212	0.832181562	0.869824553	-6.931	NoSig
CDCP1	-0.090	8.837	-0.212	0.832387585	0.869824553	-6.931	NoSig
BICD2	0.092	8.636	0.211	0.833184673	0.870038853	-6.931	NoSig
PLCG2	0.098	9.207	0.210	0.833762855	0.870038853	-6.932	NoSig
MYOZ3	-0.085	7.008	-0.208	0.83532635	0.871059104	-6.932	NoSig
PTPLA	0.135	7.541	0.197	0.843842008	0.879322401	-6.934	NoSig
PDSS2	-0.080	7.783	-0.191	0.848880538	0.883953333	-6.935	NoSig
PDE5A	-0.134	5.039	-0.183	0.854999159	0.889218466	-6.937	NoSig
DNAJC11	-0.035	9.400	-0.183	0.855132755	0.889218466	-6.937	NoSig
BTRC	-0.056	7.879	-0.180	0.857297108	0.890846121	-6.937	NoSig
DNM3	0.056	7.634	0.178	0.859135612	0.892133139	-6.938	NoSig
USP49	0.109	4.324	0.174	0.862287263	0.89471751	-6.939	NoSig
TIMM10	0.069	9.992	0.173	0.863073713	0.89471751	-6.939	NoSig
TTLL12	0.040	10.150	0.172	0.863429474	0.89471751	-6.939	NoSig
FBXO9	-0.039	11.103	-0.171	0.864619994	0.89532725	-6.939	NoSig
ZFYVE16	-0.085	7.803	-0.169	0.865593925	0.895712016	-6.939	NoSig
GOLM1	0.106	8.419	0.166	0.86850526	0.897372491	-6.940	NoSig
DYNC2H1	-0.049	7.607	-0.166	0.868593597	0.897372491	-6.940	NoSig
TGFB3	-0.068	8.420	-0.165	0.869009003	0.897372491	-6.940	NoSig
TGOLN2	0.040	10.626	0.160	0.872758321	0.900618754	-6.941	NoSig
PLD1	-0.038	8.456	-0.157	0.875119582	0.902429139	-6.941	NoSig
RUVBL2	0.082	9.996	0.154	0.877522479	0.904279921	-6.942	NoSig
CBFA2T2	-0.056	8.428	-0.150	0.880622311	0.906845829	-6.942	NoSig
TMCC2	-0.064	7.234	-0.148	0.882424726	0.908073057	-6.943	NoSig
WNK1	0.078	9.787	0.145	0.884888036	0.909978222	-6.943	NoSig
PECAM1	0.052	9.675	0.136	0.891559829	0.916205574	-6.944	NoSig
NELL1	0.052	5.010	0.129	0.897141988	0.921305342	-6.945	NoSig
CHRM3	0.084	6.649	0.126	0.900082565	0.923687214	-6.946	NoSig
LUC7L	-0.047	8.713	-0.121	0.903755236	0.926765398	-6.946	NoSig
LRRC15	0.053	8.590	0.120	0.904575964	0.926765398	-6.946	NoSig
CDK8	-0.040	7.903	-0.120	0.904951821	0.926765398	-6.946	NoSig
ITGAL	0.053	7.479	0.108	0.91431383	0.935708648	-6.948	NoSig
GYPE	-0.069	4.072	-0.106	0.915365729	0.93582114	-6.948	NoSig
VPS35	-0.041	11.249	-0.106	0.915849269	0.93582114	-6.948	NoSig
SDS	0.037	8.329	0.105	0.916311755	0.93582114	-6.948	NoSig
CCNL1	0.031	10.494	0.103	0.918283644	0.937191337	-6.948	NoSig
EHD4	0.023	9.134	0.101	0.919646617	0.937938628	-6.948	NoSig

TMEM53	0.030	8.178	0.100	0.92048521	0.93815045	-6.949	NoSig
IFT122	-0.026	8.999	-0.092	0.926464465	0.943597712	-6.949	NoSig
WARS2	-0.039	7.784	-0.092	0.927165561	0.943665427	-6.949	NoSig
CYP2E1	-0.047	6.311	-0.091	0.927915041	0.943782261	-6.949	NoSig
DOCK2	0.056	7.277	0.088	0.930055982	0.945103139	-6.950	NoSig
RCBTB2	0.051	7.514	0.087	0.930484865	0.945103139	-6.950	NoSig
MCTP1	-0.047	6.378	-0.079	0.937494369	0.951572783	-6.950	NoSig
NAV2	0.034	8.600	0.073	0.942031562	0.955525875	-6.951	NoSig
AHNAK2	0.049	8.413	0.072	0.94268719	0.955539095	-6.951	NoSig
VIL1	-0.039	5.337	-0.066	0.947391614	0.959068601	-6.951	NoSig
ATP6V1B1	-0.043	6.758	-0.066	0.947459163	0.959068601	-6.951	NoSig
CP	-0.047	8.926	-0.065	0.948127795	0.959092538	-6.951	NoSig
ATN1	-0.017	9.000	-0.063	0.950131448	0.960465984	-6.952	NoSig
INSL3	0.021	7.190	0.062	0.950850568	0.960539942	-6.952	NoSig
PCCB	0.023	9.341	0.059	0.95326455	0.96232477	-6.952	NoSig
NDUFB6	-0.024	10.442	-0.056	0.955190209	0.963330387	-6.952	NoSig
HSDL2	-0.026	7.891	-0.056	0.955556369	0.963330387	-6.952	NoSig
GALE	-0.016	7.990	-0.053	0.957475327	0.964610984	-6.952	NoSig
DOCK4	0.017	8.048	0.052	0.958675973	0.965166671	-6.952	NoSig
KRAS	0.020	10.224	0.047	0.962291569	0.968151261	-6.952	NoSig
HILPDA	-0.013	10.325	-0.042	0.966156574	0.971382574	-6.953	NoSig
PCNP	0.013	10.944	0.039	0.968953369	0.973536256	-6.953	NoSig
CCNG2	0.017	9.290	0.034	0.973101329	0.97704367	-6.953	NoSig
ADAMTSL3	0.010	6.979	0.030	0.976435992	0.97973031	-6.953	NoSig
JAK2	-0.007	8.085	-0.022	0.982525507	0.98517561	-6.953	NoSig
C1QTNF3	-0.007	7.563	-0.021	0.983219326	0.985206966	-6.953	NoSig
WTAP	-0.002	9.696	-0.005	0.995923305	0.997264616	-6.954	NoSig
RNF32	-0.001	5.178	-0.001	0.999339102	0.999598627	-6.954	NoSig
SIRPB1	0.000	6.012	-0.001	0.999598627	0.999598627	-6.954	NoSig
SYN2	-5.366	8.053	-15.049	1.03E-35	5.11E-33	70.356	Down
FGF4	-6.090	8.047	-13.572	6.32E-31	1.88E-28	59.525	Down
ADAM21	-4.302	7.885	-13.377	2.70E-30	6.68E-28	58.099	Down
KLF7	-3.452	9.514	-13.030	3.54E-29	7.53E-27	55.566	Down
PPP1R11	-3.032	11.130	-12.773	2.37E-28	3.92E-26	53.698	Down
PQLC2	-3.320	7.842	-12.560	1.15E-27	1.71E-25	52.146	Down
KDM6B	-4.637	9.638	-12.431	2.96E-27	4.00E-25	51.215	Down
CYP2A6	-5.718	8.444	-12.393	3.94E-27	4.88E-25	50.935	Down
CNOT1	-2.772	10.918	-12.373	4.56E-27	5.22E-25	50.791	Down
CNNM2	-3.245	8.512	-12.047	4.99E-26	5.30E-24	48.439	Down
GATA1	-4.824	6.633	-11.998	7.14E-26	7.08E-24	48.086	Down
LOC100506469	-3.446	9.041	-11.887	1.61E-25	1.49E-23	47.289	Down
TAF5L	-3.102	9.470	-11.847	2.15E-25	1.88E-23	47.002	Down
ZMYND11	-2.951	11.388	-11.660	8.38E-25	6.92E-23	45.665	Down
PDE1C	-4.164	7.895	-11.637	9.93E-25	7.77E-23	45.498	Down
CFL1	-4.433	14.723	-11.582	1.48E-24	1.10E-22	45.104	Down

NAGA	-2.265	9.301	-11.452	3.80E-24	2.69E-22	44.178	Down
HUNK	-3.930	7.055	-11.408	5.22E-24	3.53E-22	43.867	Down
HTR6	-5.141	7.299	-11.086	5.31E-23	3.04E-21	41.587	Down
WFDC8	-3.778	6.397	-11.059	6.45E-23	3.55E-21	41.395	Down
ZNF638	-2.651	10.599	-10.956	1.35E-22	7.17E-21	40.670	Down
PAX1	-4.924	7.180	-10.949	1.42E-22	7.28E-21	40.621	Down
EXD2	-2.359	9.021	-10.914	1.82E-22	9.02E-21	40.376	Down
NEK11	-2.221	8.792	-10.770	5.11E-22	2.37E-20	39.363	Down
COQ10B	-3.780	9.485	-10.573	2.07E-21	9.05E-20	37.988	Down
ASAH1	-3.318	12.058	-10.525	2.90E-21	1.23E-19	37.657	Down
ATM	-2.742	9.117	-10.516	3.10E-21	1.27E-19	37.590	Down
TERF2	-2.545	9.292	-10.514	3.15E-21	1.27E-19	37.575	Down
CD80	-3.057	8.572	-10.429	5.71E-21	2.18E-19	36.991	Down
TMEM151B	-3.123	7.446	-10.421	6.05E-21	2.25E-19	36.934	Down
GPR182	-3.665	7.741	-10.366	8.95E-21	3.25E-19	36.549	Down
FAM69A	-3.094	8.744	-10.325	1.19E-20	4.02E-19	36.269	Down
QKI	-3.450	10.864	-10.082	6.51E-20	2.10E-18	34.600	Down
ZNF207	-2.027	11.415	-10.041	8.71E-20	2.72E-18	34.314	Down
PIK3C2A	-4.025	9.118	-10.039	8.79E-20	2.72E-18	34.306	Down
SOS1	-3.045	8.568	-9.973	1.40E-19	4.24E-18	33.850	Down
NAP1L4	-1.957	10.005	-9.826	3.86E-19	1.13E-17	32.852	Down
FCN2	-4.031	7.756	-9.798	4.68E-19	1.34E-17	32.664	Down
SCAMP1	-2.238	10.016	-9.779	5.32E-19	1.49E-17	32.537	Down
HFE	-2.869	8.725	-9.773	5.55E-19	1.50E-17	32.496	Down
ACTR2	-2.699	11.988	-9.484	4.04E-18	1.02E-16	30.546	Down
FAM120A	-2.178	12.147	-9.424	6.06E-18	1.50E-16	30.150	Down
RQCD1	-1.648	9.983	-9.205	2.68E-17	6.32E-16	28.692	Down
SEC62	-2.524	10.438	-9.182	3.11E-17	7.22E-16	28.546	Down
ZNF146	-2.583	10.661	-9.170	3.39E-17	7.75E-16	28.461	Down
PDE8A	-2.162	8.943	-9.137	4.21E-17	9.35E-16	28.247	Down
CYP17A1	-3.821	7.477	-9.111	5.03E-17	1.10E-15	28.074	Down
STRADA	-2.295	10.005	-8.849	2.87E-16	5.80E-15	26.365	Down
CCDC40	-2.921	7.392	-8.848	2.89E-16	5.80E-15	26.360	Down
PAX8	-3.352	10.387	-8.831	3.24E-16	6.33E-15	26.248	Down
PPM1F	-2.172	8.946	-8.788	4.31E-16	8.32E-15	25.967	Down
RASGRF1	-2.870	7.574	-8.699	7.70E-16	1.45E-14	25.399	Down
BRS3	-3.980	6.605	-8.662	9.84E-16	1.83E-14	25.159	Down
APH1A	-2.533	10.656	-8.622	1.28E-15	2.34E-14	24.904	Down
SNRK	-2.208	9.420	-8.580	1.69E-15	3.06E-14	24.630	Down
PACSIN2	-2.396	10.795	-8.567	1.84E-15	3.29E-14	24.547	Down
ARIH1	-2.128	9.317	-8.396	5.54E-15	9.58E-14	23.466	Down
RAC1	-1.956	12.639	-8.319	9.11E-15	1.54E-13	22.978	Down
GUCA1A	-3.811	6.741	-8.261	1.32E-14	2.18E-13	22.615	Down
GRIK5	-3.854	8.226	-8.255	1.38E-14	2.25E-13	22.574	Down
CCRN4L	-1.969	8.601	-8.226	1.65E-14	2.67E-13	22.395	Down

SLC4A4	-4.262	8.677	-8.173	2.32E-14	3.71E-13	22.062	Down
SLC5A3	-2.897	9.625	-8.094	3.84E-14	6.07E-13	21.571	Down
TYR	-3.961	6.686	-8.068	4.50E-14	7.01E-13	21.414	Down
MTDH	-2.302	11.689	-8.068	4.53E-14	7.01E-13	21.409	Down
ZMYM3	-1.809	9.377	-8.029	5.78E-14	8.86E-13	21.170	Down
GALNT1	-2.049	11.187	-8.022	6.03E-14	9.16E-13	21.128	Down
SLC36A1	-3.397	7.891	-7.967	8.52E-14	1.28E-12	20.791	Down
LARP4	-1.920	9.665	-7.965	8.64E-14	1.29E-12	20.777	Down
SLC17A1	-3.939	7.000	-7.905	1.26E-13	1.85E-12	20.408	Down
CRTAC1	-3.667	7.285	-7.904	1.27E-13	1.85E-12	20.401	Down
RHOB	-3.214	9.587	-7.869	1.58E-13	2.27E-12	20.189	Down
RTN4	-2.266	12.325	-7.847	1.81E-13	2.58E-12	20.056	Down
DNAH3	-4.609	10.157	-7.810	2.28E-13	3.17E-12	19.827	Down
MYT1	-4.506	7.450	-7.805	2.35E-13	3.23E-12	19.800	Down
HSP90AB1	-2.488	13.983	-7.780	2.74E-13	3.74E-12	19.648	Down
PLXNA3	-2.236	8.269	-7.766	2.99E-13	4.04E-12	19.564	Down
DRP2	-4.130	7.456	-7.750	3.30E-13	4.39E-12	19.465	Down
CEPT1	-2.115	8.858	-7.723	3.90E-13	5.13E-12	19.304	Down
NOX5	-3.105	6.957	-7.682	5.02E-13	6.55E-12	19.057	Down
DNAH6	-2.100	6.005	-7.669	5.46E-13	7.06E-12	18.974	Down
PRO2949	-3.591	6.443	-7.663	5.66E-13	7.26E-12	18.939	Down
RAPGEF5	-2.039	8.462	-7.654	5.97E-13	7.59E-12	18.887	Down
ZNF160	-3.053	10.705	-7.649	6.17E-13	7.77E-12	18.855	Down
CACNA1D	-2.727	7.546	-7.600	8.34E-13	1.03E-11	18.561	Down
C5orf22	-2.005	9.424	-7.579	9.47E-13	1.16E-11	18.436	Down
EXD3	-2.545	7.857	-7.563	1.04E-12	1.27E-11	18.342	Down
HMGA2	-3.654	7.158	-7.528	1.29E-12	1.55E-11	18.132	Down
RPP30	-1.657	9.336	-7.514	1.41E-12	1.66E-11	18.050	Down
SV2C	-3.057	6.278	-7.441	2.19E-12	2.53E-11	17.616	Down
SNAP25	-4.429	7.813	-7.377	3.22E-12	3.65E-11	17.241	Down
RERE	-2.214	9.454	-7.330	4.27E-12	4.81E-11	16.965	Down
GNG12	-2.686	9.599	-7.306	4.95E-12	5.53E-11	16.822	Down
KLHDC4	-2.092	8.357	-7.284	5.63E-12	6.25E-11	16.696	Down
DSG2	-3.707	10.797	-7.277	5.90E-12	6.49E-11	16.651	Down
FARP2	-2.162	7.798	-7.273	6.01E-12	6.58E-11	16.632	Down
PGC	-3.302	7.362	-7.254	6.75E-12	7.27E-11	16.519	Down
TRAM2	-1.907	9.091	-7.211	8.72E-12	9.13E-11	16.269	Down
LRRC41	-2.489	9.072	-7.089	1.79E-11	1.85E-10	15.567	Down
ATP4B	-3.100	6.160	-7.074	1.96E-11	2.01E-10	15.479	Down
TRPM3	-2.886	7.827	-7.040	2.39E-11	2.44E-10	15.285	Down
LRRC48	-2.039	7.395	-7.015	2.77E-11	2.77E-10	15.141	Down
MCCC2	-2.383	9.851	-7.013	2.80E-11	2.77E-10	15.133	Down
TOR1AIP1	-2.352	9.710	-7.005	2.93E-11	2.88E-10	15.086	Down
SRGAP3	-2.264	8.202	-7.005	2.94E-11	2.88E-10	15.084	Down
PNN	-1.627	12.626	-6.974	3.52E-11	3.40E-10	14.909	Down

DCTN5	-1.784	9.042	-6.959	3.83E-11	3.66E-10	14.825	Down
NEUROD2	-3.140	7.596	-6.918	4.89E-11	4.63E-10	14.589	Down
GHITM	-1.716	11.738	-6.862	6.73E-11	6.29E-10	14.277	Down
MPHOSPH6	-2.066	10.142	-6.854	7.07E-11	6.57E-10	14.229	Down
DOK5	-2.163	8.999	-6.771	1.13E-10	1.03E-09	13.769	Down
FBXW2	-1.681	9.043	-6.759	1.22E-10	1.10E-09	13.699	Down
RGS7	-3.201	5.962	-6.750	1.28E-10	1.14E-09	13.652	Down
ENTPD5	-2.271	7.713	-6.715	1.57E-10	1.36E-09	13.453	Down
GABRB2	-3.340	6.939	-6.698	1.73E-10	1.49E-09	13.358	Down
NOL9	-1.305	9.661	-6.688	1.83E-10	1.56E-09	13.305	Down
PRR14L	-2.356	8.622	-6.679	1.93E-10	1.64E-09	13.254	Down
LMO3	-3.350	7.889	-6.670	2.02E-10	1.69E-09	13.205	Down
SERPINB6	-2.034	9.941	-6.650	2.26E-10	1.86E-09	13.098	Down
ZNF85	-1.647	9.392	-6.641	2.38E-10	1.94E-09	13.049	Down
SLC39A6	-2.674	11.131	-6.636	2.45E-10	1.99E-09	13.019	Down
TNR	-1.987	7.305	-6.635	2.46E-10	1.99E-09	13.015	Down
DIP2A	-2.643	9.013	-6.623	2.63E-10	2.11E-09	12.950	Down
GOSR1	-1.396	9.473	-6.613	2.79E-10	2.23E-09	12.894	Down
ZNF611	-2.206	9.513	-6.555	3.87E-10	3.08E-09	12.575	Down
CLEC7A	-3.602	9.423	-6.538	4.25E-10	3.36E-09	12.483	Down
MOSPD1	-1.515	8.952	-6.520	4.71E-10	3.69E-09	12.384	Down
CYP19A1	-3.813	6.204	-6.505	5.12E-10	3.96E-09	12.303	Down
ADAMTS20	-2.964	5.952	-6.446	7.10E-10	5.47E-09	11.985	Down
DPP6	-2.673	6.916	-6.442	7.26E-10	5.56E-09	11.964	Down
CCDC7	-2.224	7.809	-6.433	7.63E-10	5.81E-09	11.916	Down
GABRG3	-2.690	6.102	-6.427	7.88E-10	5.98E-09	11.883	Down
GAD2	-3.478	6.076	-6.426	7.96E-10	5.98E-09	11.874	Down
ZKSCAN3	-2.098	8.416	-6.425	7.96E-10	5.98E-09	11.874	Down
LRRC16A	-1.624	8.743	-6.423	8.06E-10	6.02E-09	11.862	Down
TTLL5	-1.368	8.637	-6.419	8.26E-10	6.14E-09	11.838	Down
KAT6B	-2.507	8.770	-6.375	1.05E-09	7.71E-09	11.603	Down
CANT1	-1.983	9.540	-6.363	1.13E-09	8.20E-09	11.538	Down
ZNF33B	-2.176	7.936	-6.315	1.46E-09	1.05E-08	11.283	Down
HDAC9	-3.158	7.484	-6.276	1.81E-09	1.29E-08	11.077	Down
GPR97	-3.341	7.486	-6.236	2.25E-09	1.59E-08	10.865	Down
YTHDF3	-2.212	9.534	-6.231	2.31E-09	1.63E-08	10.837	Down
PDZD3	-2.187	5.401	-6.220	2.45E-09	1.72E-08	10.781	Down
GABRA2	-2.879	5.519	-6.213	2.56E-09	1.78E-08	10.741	Down
JAK1	-1.807	10.644	-6.204	2.67E-09	1.86E-08	10.698	Down
C1orf61	-3.114	8.034	-6.193	2.85E-09	1.97E-08	10.636	Down
GYPA	-3.318	5.196	-6.166	3.28E-09	2.25E-08	10.499	Down
SPEN	-1.130	9.826	-6.163	3.33E-09	2.27E-08	10.483	Down
SLC17A4	-3.910	6.585	-6.138	3.83E-09	2.60E-08	10.350	Down
NAA15	-1.782	8.521	-6.109	4.47E-09	3.02E-08	10.200	Down
TAT	-3.500	6.860	-6.070	5.51E-09	3.67E-08	9.997	Down

ARHGAP29	-2.633	9.750	-6.064	5.67E-09	3.76E-08	9.969	Down
KIAA1467	-2.467	8.402	-6.055	5.97E-09	3.94E-08	9.919	Down
TBC1D12	-1.430	8.356	-6.045	6.27E-09	4.13E-08	9.871	Down
POLR3B	-1.294	8.805	-5.991	8.36E-09	5.43E-08	9.592	Down
FSTL4	-1.925	5.855	-5.946	1.06E-08	6.83E-08	9.361	Down
ITSN1	-2.034	8.838	-5.885	1.46E-08	9.29E-08	9.051	Down
CRISPLD2	-2.380	9.025	-5.845	1.81E-08	1.14E-07	8.847	Down
JRK	-1.595	9.076	-5.754	2.88E-08	1.75E-07	8.396	Down
ABCC9	-2.058	7.411	-5.745	3.02E-08	1.83E-07	8.351	Down
STAT5B	-1.523	9.003	-5.742	3.07E-08	1.86E-07	8.332	Down
RIMS1	-3.298	6.192	-5.737	3.15E-08	1.89E-07	8.308	Down
CDH4	-2.564	7.494	-5.734	3.20E-08	1.91E-07	8.294	Down
LRCH3	-1.628	8.004	-5.728	3.30E-08	1.97E-07	8.262	Down
ZDHHC18	-1.918	8.802	-5.687	4.07E-08	2.38E-07	8.061	Down
DNAH17	-2.346	6.885	-5.672	4.38E-08	2.54E-07	7.991	Down
UBA1	-2.334	11.492	-5.662	4.61E-08	2.66E-07	7.941	Down
STX6	-1.176	9.943	-5.636	5.28E-08	3.02E-07	7.810	Down
PIK3R2	-1.740	8.284	-5.610	6.02E-08	3.43E-07	7.683	Down
NDST1	-1.748	8.332	-5.608	6.06E-08	3.44E-07	7.676	Down
SH3YL1	-2.651	10.795	-5.607	6.11E-08	3.45E-07	7.668	Down
MAPK1	-1.361	9.903	-5.606	6.13E-08	3.45E-07	7.666	Down
PTPRO	-5.373	11.908	-5.584	6.87E-08	3.84E-07	7.556	Down
COPA	-2.706	11.245	-5.571	7.31E-08	4.04E-07	7.495	Down
MS4A3	-3.182	5.161	-5.555	7.94E-08	4.36E-07	7.415	Down
TRMT2B	-1.972	8.834	-5.539	8.61E-08	4.71E-07	7.337	Down
FBXL14	-1.684	8.717	-5.518	9.55E-08	5.16E-07	7.238	Down
TIA1	-1.462	10.520	-5.506	1.01E-07	5.46E-07	7.180	Down
CACNB2	-1.907	7.228	-5.489	1.10E-07	5.87E-07	7.097	Down
SAMHD1	-2.661	8.762	-5.476	1.18E-07	6.23E-07	7.035	Down
NRXN1	-2.978	5.971	-5.451	1.33E-07	7.01E-07	6.915	Down
IRF6	-1.779	10.533	-5.438	1.42E-07	7.44E-07	6.854	Down
CNKSR2	-2.884	6.546	-5.434	1.45E-07	7.55E-07	6.837	Down
VPS53	-1.694	7.370	-5.425	1.52E-07	7.88E-07	6.792	Down
LILRA5	-2.204	7.160	-5.414	1.60E-07	8.22E-07	6.742	Down
VPS8	-1.211	8.594	-5.395	1.76E-07	8.98E-07	6.650	Down
NEK1	-1.455	8.315	-5.342	2.28E-07	1.15E-06	6.401	Down
GRM5	-2.906	5.457	-5.230	3.92E-07	1.92E-06	5.877	Down
AFG3L2	-1.385	10.050	-5.214	4.23E-07	2.04E-06	5.805	Down
SLC4A5	-1.822	6.975	-5.214	4.23E-07	2.04E-06	5.804	Down
ZNF586	-1.378	8.424	-5.197	4.61E-07	2.20E-06	5.722	Down
UBXN4	-2.742	10.366	-5.173	5.15E-07	2.44E-06	5.616	Down
NOS1	-2.496	7.840	-5.096	7.42E-07	3.45E-06	5.264	Down
KCNK10	-2.148	6.315	-5.082	7.92E-07	3.67E-06	5.201	Down
SMAD4	-1.243	8.607	-5.078	8.10E-07	3.74E-06	5.180	Down
CXADR	-2.784	10.313	-5.060	8.80E-07	4.04E-06	5.101	Down

SMC1A	-1.654	10.464	-5.048	9.31E-07	4.25E-06	5.046	Down
F11	-2.743	6.200	-5.047	9.36E-07	4.26E-06	5.042	Down
TTN	-1.680	7.736	-5.029	1.02E-06	4.62E-06	4.959	Down
PPA2	-1.278	10.254	-5.028	1.03E-06	4.63E-06	4.954	Down
SRPK2	-1.546	9.918	-5.020	1.06E-06	4.78E-06	4.921	Down
EYA4	-2.657	5.994	-5.009	1.12E-06	5.03E-06	4.869	Down
SIRT2	-1.502	9.816	-4.994	1.20E-06	5.35E-06	4.804	Down
MCM9	-1.418	8.358	-4.990	1.22E-06	5.44E-06	4.785	Down
RHOA	-1.324	13.246	-4.970	1.34E-06	5.93E-06	4.698	Down
EGFR	-2.094	8.493	-4.937	1.56E-06	6.82E-06	4.551	Down
GPR12	-1.959	8.307	-4.910	1.77E-06	7.65E-06	4.433	Down
STK38	-1.238	10.208	-4.889	1.95E-06	8.37E-06	4.339	Down
PIAS2	-1.441	8.872	-4.888	1.96E-06	8.38E-06	4.335	Down
INPP5A	-1.092	9.046	-4.857	2.25E-06	9.60E-06	4.199	Down
ADAM23	-2.018	7.336	-4.843	2.40E-06	1.02E-05	4.140	Down
PIGL	-1.865	8.282	-4.817	2.71E-06	1.14E-05	4.023	Down
NDUFA10	-1.171	10.359	-4.795	2.99E-06	1.26E-05	3.929	Down
SLC1A2	-1.929	7.331	-4.784	3.14E-06	1.31E-05	3.882	Down
DR1	-1.101	9.644	-4.772	3.32E-06	1.37E-05	3.829	Down
CSNK1A1	-1.249	11.439	-4.761	3.48E-06	1.44E-05	3.784	Down
ZNF654	-1.752	7.889	-4.724	4.10E-06	1.68E-05	3.627	Down
MBTPS2	-1.728	7.666	-4.720	4.19E-06	1.71E-05	3.606	Down
RBM15	-1.951	9.122	-4.714	4.30E-06	1.74E-05	3.581	Down
NR1H4	-2.593	5.616	-4.700	4.57E-06	1.84E-05	3.523	Down
TAF5	-1.756	8.315	-4.698	4.62E-06	1.85E-05	3.512	Down
HEATR2	-1.172	10.054	-4.659	5.48E-06	2.18E-05	3.350	Down
IRAK1	-2.043	11.583	-4.657	5.54E-06	2.20E-05	3.339	Down
LYZ	-2.976	11.957	-4.636	6.08E-06	2.39E-05	3.251	Down
IKZF1	-1.547	9.216	-4.630	6.23E-06	2.44E-05	3.228	Down
KLHL4	-2.019	6.654	-4.627	6.32E-06	2.47E-05	3.214	Down
DMWD	-1.142	10.018	-4.612	6.73E-06	2.62E-05	3.153	Down
MACF1	-1.454	10.452	-4.606	6.93E-06	2.68E-05	3.126	Down
TMEM126B	-1.443	10.355	-4.584	7.64E-06	2.94E-05	3.033	Down
ABCD3	-1.829	9.875	-4.571	8.07E-06	3.09E-05	2.980	Down
ACSM5	-2.109	7.355	-4.570	8.12E-06	3.10E-05	2.975	Down
AMMECR1	-1.451	8.635	-4.559	8.51E-06	3.24E-05	2.930	Down
DNTT	-1.903	4.182	-4.550	8.86E-06	3.35E-05	2.892	Down
LRPPRC	-1.282	10.936	-4.470	1.25E-05	4.63E-05	2.564	Down
ERCC8	-1.248	8.116	-4.467	1.26E-05	4.68E-05	2.553	Down
HSD17B12	-1.690	11.148	-4.462	1.29E-05	4.75E-05	2.534	Down
CTNNA3	-2.235	4.930	-4.443	1.40E-05	5.13E-05	2.456	Down
FBXL18	-1.060	7.816	-4.440	1.42E-05	5.18E-05	2.444	Down
CD74	-2.329	12.643	-4.416	1.57E-05	5.71E-05	2.347	Down
AGK	-1.812	8.779	-4.410	1.61E-05	5.86E-05	2.321	Down
DYNLRB1	-1.627	11.419	-4.408	1.63E-05	5.88E-05	2.314	Down

MYT1L	-2.368	5.586	-4.401	1.67E-05	6.04E-05	2.286	Down
ZNF264	-1.511	8.534	-4.387	1.78E-05	6.40E-05	2.227	Down
TBX5	-2.073	6.911	-4.384	1.80E-05	6.43E-05	2.218	Down
ACACB	-2.216	7.518	-4.379	1.84E-05	6.55E-05	2.199	Down
RECK	-2.746	11.320	-4.331	2.25E-05	7.95E-05	2.006	Down
CCND3	-1.298	9.981	-4.310	2.46E-05	8.62E-05	1.922	Down
NTRK3	-1.826	8.549	-4.265	2.97E-05	0.000103547	1.744	Down
TRPV5	-1.794	7.042	-4.241	3.27E-05	0.000112984	1.650	Down
NR5A2	-2.105	6.356	-4.199	3.88E-05	0.000132013	1.489	Down
CAMKMT	-1.813	6.859	-4.185	4.11E-05	0.000139484	1.435	Down
CTTN	-3.129	10.261	-4.138	4.99E-05	0.000168158	1.252	Down
CTNNA1	-1.124	11.394	-4.101	5.78E-05	0.000193723	1.112	Down
CHPT1	-1.649	10.308	-4.087	6.12E-05	0.000203491	1.059	Down
MDFIC	-1.785	9.518	-4.076	6.40E-05	0.000211855	1.017	Down
KIF5C	-1.493	8.274	-4.072	6.50E-05	0.000214696	1.002	Down
TMC7	-1.965	6.071	-4.013	8.20E-05	0.0002674	0.783	Down
GNB5	-1.272	7.649	-3.999	8.67E-05	0.000281968	0.731	Down
TET3	-1.637	6.941	-3.980	9.36E-05	0.000301254	0.658	Down
ATF6B	-1.069	8.682	-3.960	0.00010115	0.000323697	0.585	Down
POLR2B	-1.240	11.307	-3.944	0.000107591	0.000341853	0.527	Down
TXLNA	-1.682	8.507	-3.938	0.000109975	0.000347941	0.506	Down
NUMA1	-1.302	9.475	-3.925	0.000115859	0.000365005	0.457	Down
ADAM9	-1.891	10.039	-3.910	0.000122901	0.000386372	0.402	Down
ENTPD3	-1.915	7.310	-3.887	0.000133942	0.000418898	0.321	Down
ZFHX4	-1.475	7.989	-3.887	0.000134092	0.000418898	0.320	Down
PRF1	-1.794	8.739	-3.864	0.000146829	0.000455813	0.235	Down
SIK2	-1.560	7.803	-3.859	0.000149682	0.000462738	0.216	Down
SCTR	-2.222	5.773	-3.835	0.000163461	0.000503242	0.134	Down
ZNF81	-2.514	5.954	-3.819	0.000174069	0.0005315	0.075	Down
IGSF3	-1.194	9.827	-3.815	0.000176876	0.000538964	0.060	Down
RHOQ	-1.399	10.226	-3.789	0.000194728	0.00059094	-0.030	Down
KIDINS220	-1.046	9.493	-3.779	0.000202796	0.00061168	-0.068	Down
ARHGAP5	-1.043	8.510	-3.777	0.000204313	0.000615008	-0.075	Down
ANGPT1	-1.596	8.391	-3.747	0.000228406	0.000682007	-0.180	Down
ARHGEF2	-1.422	10.397	-3.731	0.000242393	0.000719077	-0.235	Down
FOXJ2	-1.130	9.237	-3.731	0.000242755	0.000719077	-0.237	Down
ROCK1	-1.772	9.852	-3.700	0.000272389	0.000801688	-0.344	Down
TRIM37	-1.080	9.596	-3.682	0.000290957	0.000851678	-0.406	Down
TAL1	-1.702	6.864	-3.662	0.000313686	0.000912821	-0.476	Down
NMNAT2	-2.115	6.355	-3.656	0.000319724	0.000928573	-0.494	Down
C12orf49	-1.669	9.113	-3.649	0.000327955	0.000945096	-0.518	Down
NCALD	-1.853	8.777	-3.641	0.000337979	0.00097166	-0.546	Down
TXNRD1	-2.625	9.337	-3.641	0.00033848	0.00097166	-0.547	Down
SUOX	-1.309	8.597	-3.639	0.000341182	0.000973778	-0.555	Down
RFC2	-1.063	8.975	-3.627	0.000356316	0.001013065	-0.595	Down

SYNJ2	-1.075	8.770	-3.620	0.000365344	0.001032827	-0.618	Down
DIP2C	-1.258	9.448	-3.597	0.000397635	0.001113529	-0.697	Down
PDE4D	-1.674	7.266	-3.581	0.000421042	0.001176861	-0.751	Down
SLC44A4	-2.287	5.964	-3.548	0.000474109	0.001320224	-0.861	Down
MOCS1	-1.145	7.555	-3.534	0.000497682	0.001383277	-0.906	Down
ELOVL5	-1.758	10.299	-3.532	0.00050122	0.001390511	-0.913	Down
PRDM11	-1.369	7.404	-3.529	0.000506854	0.001403524	-0.923	Down
NID1	-1.187	7.675	-3.492	0.000578028	0.001594671	-1.045	Down
TYMS	-1.432	11.835	-3.484	0.000595849	0.001637758	-1.074	Down
ERBB3	-1.390	9.885	-3.477	0.000610984	0.001673174	-1.097	Down
MOG	-1.820	6.683	-3.470	0.000625095	0.001705535	-1.118	Down
MCF2	-2.077	5.626	-3.464	0.000637867	0.001737196	-1.137	Down
RHOF	-1.129	8.370	-3.461	0.000646619	0.00175781	-1.149	Down
GPR6	-2.057	4.966	-3.452	0.000665398	0.001797397	-1.176	Down
BNC1	-1.913	6.472	-3.452	0.000666016	0.001797397	-1.177	Down
ALPL	-1.386	6.869	-3.443	0.000688086	0.001849669	-1.207	Down
LYST	-1.067	9.273	-3.441	0.000693447	0.001851266	-1.214	Down
SCN1A	-2.358	4.164	-3.435	0.000708693	0.001878478	-1.234	Down
AKR1C1	-2.385	9.630	-3.411	0.000770233	0.002027143	-1.312	Down
CEP112	-1.383	6.445	-3.408	0.000778129	0.002044308	-1.321	Down
DLEU2	-1.031	7.546	-3.383	0.000848178	0.002212702	-1.401	Down
B4GALNT1	-1.492	7.481	-3.369	0.00088865	0.002306149	-1.444	Down
SP140L	-1.458	8.115	-3.353	0.000941429	0.002430392	-1.497	Down
ZNF451	-1.109	8.797	-3.348	0.000956042	0.002459575	-1.511	Down
CD55	-1.219	9.828	-3.307	0.001098996	0.002803099	-1.640	Down
RAP2A	-1.480	10.334	-3.294	0.00114899	0.002910644	-1.681	Down
GSTCD	-1.297	7.488	-3.293	0.001153857	0.002918001	-1.685	Down
STAP1	-2.255	6.308	-3.281	0.001202106	0.003024588	-1.723	Down
ALDH3B1	-1.301	7.933	-3.275	0.001226531	0.003077603	-1.741	Down
SLC6A11	-1.873	5.159	-3.273	0.00123377	0.003088579	-1.747	Down
PAPPA	-1.397	6.645	-3.267	0.001261068	0.003141052	-1.767	Down
BTG4	-1.588	4.207	-3.244	0.001362482	0.003376685	-1.838	Down
FAM208B	-1.157	9.845	-3.240	0.001380125	0.003414718	-1.850	Down
EBF2	-1.768	5.783	-3.226	0.001444204	0.003567329	-1.892	Down
CBY1	-1.124	9.267	-3.213	0.001509604	0.003710382	-1.932	Down
C6orf123	-1.469	6.798	-3.208	0.001535954	0.003768916	-1.948	Down
KCNQ3	-1.139	7.317	-3.178	0.001692855	0.004126681	-2.037	Down
IDH1	-1.019	10.959	-3.171	0.001732319	0.00421597	-2.059	Down
SSBP1	-1.109	10.950	-3.110	0.00211528	0.005089678	-2.242	Down
HOMER2	-1.305	8.182	-3.104	0.002159749	0.005186559	-2.261	Down
ARVCF	-1.235	8.453	-3.082	0.002319581	0.005518747	-2.326	Down
NEDD9	-1.220	8.120	-3.041	0.002646556	0.006197526	-2.446	Down
ARHGEF12	-1.151	9.302	-3.030	0.002733362	0.006360735	-2.476	Down
ARPP21	-1.549	4.215	-3.023	0.002801174	0.0064982	-2.498	Down
MYLK3	-1.739	4.910	-3.019	0.00283411	0.006543977	-2.509	Down

RBFOX1	-1.510	6.440	-3.011	0.002902616	0.006691768	-2.530	Down
VRK3	-1.055	8.714	-2.991	0.003092006	0.007117359	-2.588	Down
BCL2L14	-1.215	8.561	-2.982	0.003182728	0.007292321	-2.614	Down
IL16	-1.018	8.871	-2.980	0.003202741	0.007326886	-2.620	Down
RGR	-1.817	6.436	-2.967	0.003337522	0.007600145	-2.657	Down
PTK2	-1.253	10.081	-2.951	0.003509413	0.007967171	-2.703	Down
GLS2	-1.294	6.120	-2.946	0.003561811	0.008037046	-2.716	Down
VNN3	-1.670	5.716	-2.945	0.003578121	0.008058681	-2.720	Down
GVINP1	-1.070	8.106	-2.942	0.003605897	0.00809965	-2.727	Down
GABRB1	-1.450	6.441	-2.941	0.003624646	0.008129485	-2.732	Down
SLC1A6	-1.363	5.041	-2.932	0.003725313	0.008317629	-2.757	Down
CNDP2	-1.117	10.634	-2.927	0.003776613	0.008415807	-2.769	Down
POSTN	-2.357	10.900	-2.927	0.003780604	0.008415807	-2.770	Down
SNTG1	-1.318	6.512	-2.926	0.003792862	0.008430471	-2.773	Down
HIF3A	-1.267	6.976	-2.918	0.003890544	0.008621817	-2.796	Down
CA6	-1.872	6.813	-2.879	0.004384739	0.009616676	-2.905	Down
FHL5	-1.775	5.607	-2.852	0.004757542	0.010373115	-2.978	Down
KCNIP2	-1.323	6.733	-2.840	0.004925516	0.010705144	-3.010	Down
ABCC4	-1.482	7.052	-2.840	0.004931631	0.010705144	-3.011	Down
TTBK2	-1.182	6.625	-2.811	0.005388936	0.011546611	-3.091	Down
TLE6	-1.014	6.729	-2.805	0.005476174	0.011699815	-3.105	Down
SLC11A1	-1.770	8.358	-2.796	0.00563061	0.012012506	-3.130	Down
EDIL3	-1.166	6.812	-2.792	0.005700832	0.012144895	-3.141	Down
MBD1	-1.093	9.255	-2.789	0.005753283	0.012239101	-3.150	Down
LITAF	-2.129	10.759	-2.775	0.005984903	0.012659389	-3.185	Down
SLC38A4	-1.527	4.849	-2.735	0.006743722	0.014123822	-3.292	Down
RAPGEF6	-1.026	8.052	-2.684	0.007824054	0.01606957	-3.425	Down
CELF2	-1.149	9.867	-2.678	0.007953918	0.016313759	-3.440	Down
DST	-1.149	9.300	-2.663	0.00831351	0.01698103	-3.479	Down
BICD1	-1.213	7.895	-2.649	0.008652778	0.017625591	-3.515	Down
DOCK5	-1.085	7.351	-2.614	0.009553316	0.019301332	-3.603	Down
POLR1B	-1.257	9.098	-2.606	0.009785802	0.019744216	-3.625	Down
GATA4	-1.504	4.913	-2.597	0.010046979	0.02024371	-3.648	Down
TNPO1	-1.270	10.887	-2.585	0.01037647	0.020822957	-3.677	Down
PIEZO2	-1.050	7.521	-2.561	0.011117953	0.022220962	-3.738	Down
CADPS	-1.186	6.159	-2.548	0.011502572	0.022928048	-3.768	Down
HBB	-3.236	13.381	-2.485	0.013679059	0.027084901	-3.921	Down
SHANK2	-1.119	8.358	-2.484	0.013719637	0.027129122	-3.924	Down
ADAM22	-1.281	7.566	-2.460	0.01467511	0.028750841	-3.983	Down
PADI2	-1.097	10.255	-2.428	0.015976912	0.030814096	-4.058	Down
SLC4A1	-1.958	6.925	-2.392	0.017602438	0.033643734	-4.142	Down
HIST1H1T	-1.337	5.127	-2.388	0.017760079	0.03390146	-4.150	Down
SCIN	-1.223	6.672	-2.368	0.018721686	0.035645515	-4.196	Down
CLCNKB	-1.108	5.048	-2.344	0.019945113	0.037829569	-4.251	Down
FGF22	-1.212	5.956	-2.320	0.021239007	0.040028395	-4.306	Down

PPFIA2	-1.093	5.786	-2.315	0.02154438	0.040399109	-4.319	Down
SLC18A2	-1.377	4.643	-2.266	0.024429602	0.044958933	-4.427	Down
JMJD6	-1.015	8.772	-2.260	0.024789678	0.045508952	-4.440	Down
TLL1	-1.054	5.565	-2.237	0.026309127	0.048061022	-4.491	Down
SPOCK3	-1.491	4.891	-2.223	0.02723417	0.049628935	-4.521	Down

Table S5. Significant genes for the prediction of pCR rate

Gene	OR	Low.95.CI	High.95.CI	P.value
ATP4B	0.669	0.523	0.856	0.001
FBXO22	1.372	1.141	1.649	0.001
SMEK2	1.456	1.072	1.977	0.016
FCN2	0.751	0.592	0.951	0.018
RRP8	1.512	1.051	2.175	0.026
TET3	1.361	1.034	1.792	0.028

Table S6. Results of binary logistic regression analysis

Variables	P.Value
Age	0.839
Clinical stage	
I	Reference
IIA	0.898
IIB	0.724
IIIA	0.395
IIIB	0.248
IIIC	0.716
T stage	
T1	Reference
T2	0.113
T3	0.007
T4	0.026
Nodal status	
Negative	Reference
Positive	0.279
Grade	
Grade1	Reference
Grade2	0.182
Grade3	0.065
pRS	<0.0001

Table S7. Differentially expressed genes of high pRS low pRS group

Gene	logFC	AveExpr	t	P.Value	adj.P.Val	B	threshold
GIT2	1.376	5.944	7.404	2.73E-12	2.05E-09	17.501	Up
DYNC1H1	1.214	10.082	7.403	2.75E-12	2.05E-09	17.493	Up
FN1	1.469	12.424	7.213	8.59E-12	2.30E-09	16.398	Up
CNPY2	1.074	10.798	7.078	1.91E-11	4.06E-09	15.629	Up
PTPN11	1.136	10.243	6.986	3.28E-11	6.10E-09	15.109	Up
STOML1	1.108	7.961	6.846	7.39E-11	1.01E-08	14.328	Up
CMTM6	1.836	10.561	6.843	7.50E-11	1.01E-08	14.315	Up
DIAPH1	1.336	9.108	6.636	2.45E-10	2.28E-08	13.177	Up
LEPROT	1.872	9.973	6.589	3.19E-10	2.56E-08	12.924	Up
ROCK1	1.241	9.852	6.364	1.12E-09	6.16E-08	11.721	Up
FAM189B	1.646	9.116	6.253	2.04E-09	1.07E-07	11.143	Up
PHIP	1.312	9.514	6.250	2.09E-09	1.07E-07	11.123	Up
PABPC1	1.331	14.264	6.125	4.10E-09	1.69E-07	10.477	Up
EEF1A1	2.105	14.942	6.104	4.59E-09	1.80E-07	10.369	Up
KPNA4	1.375	7.931	6.091	4.91E-09	1.87E-07	10.304	Up
PRSS23	1.336	8.319	6.066	5.61E-09	1.99E-07	10.177	Up
TPM1	1.022	11.448	6.028	6.88E-09	2.27E-07	9.981	Up
UBR4	1.180	9.429	5.978	8.94E-09	2.71E-07	9.731	Up
CCNG2	1.221	9.290	5.961	9.78E-09	2.81E-07	9.645	Up
AKAP12	1.221	7.014	5.957	1.00E-08	2.81E-07	9.623	Up
TUBA1B	2.114	13.659	5.934	1.13E-08	3.06E-07	9.505	Up
TNPO1	1.160	10.887	5.898	1.36E-08	3.44E-07	9.329	Up
ANXA2	1.254	13.283	5.898	1.37E-08	3.44E-07	9.326	Up
RPS15	1.678	13.631	5.846	1.79E-08	4.22E-07	9.069	Up
A2M	1.333	11.007	5.746	3.00E-08	6.28E-07	8.575	Up
WDR3	1.117	9.059	5.719	3.45E-08	7.13E-07	8.440	Up
MEX3C	1.169	8.127	5.707	3.67E-08	7.37E-07	8.383	Up
MMP2	1.166	9.081	5.662	4.61E-08	8.81E-07	8.163	Up
SSPN	1.098	7.376	5.550	8.13E-08	1.46E-06	7.623	Up
HOPX	1.132	7.998	5.525	9.19E-08	1.59E-06	7.507	Up
ALG5	1.205	9.823	5.486	1.12E-07	1.89E-06	7.318	Up
VIM	1.315	13.257	5.454	1.31E-07	2.08E-06	7.169	Up
RCBTB2	1.173	7.514	5.413	1.61E-07	2.44E-06	6.972	Up
RPS9	1.606	12.465	5.409	1.64E-07	2.46E-06	6.958	Up
TMED5	1.086	9.879	5.405	1.67E-07	2.49E-06	6.937	Up
EIF4G2	1.308	12.143	5.336	2.34E-07	3.19E-06	6.617	Up
RNH1	1.253	9.947	5.294	2.87E-07	3.71E-06	6.422	Up
STMN1	1.121	9.089	5.201	4.51E-07	5.32E-06	5.994	Up
BTG3	1.095	10.019	5.161	5.46E-07	6.15E-06	5.812	Up
EMP1	1.147	9.480	5.120	6.63E-07	7.01E-06	5.629	Up

TXNRD1	1.701	9.337	5.087	7.75E-07	7.79E-06	5.480	Up
CTTN	1.680	10.261	5.032	1.00E-06	9.58E-06	5.234	Up
RAP1A	1.163	9.689	4.981	1.27E-06	1.17E-05	5.011	Up
ELOVL5	1.010	10.299	4.965	1.37E-06	1.25E-05	4.938	Up
LGALS3	1.357	11.952	4.942	1.52E-06	1.34E-05	4.840	Up
STK24	1.245	11.463	4.802	2.89E-06	2.29E-05	4.234	Up
FUT6	1.070	9.630	4.633	6.14E-06	4.33E-05	3.523	Up
WNK1	1.027	9.787	4.598	7.16E-06	4.91E-05	3.378	Up
ERGIC3	1.057	10.543	4.481	1.19E-05	7.53E-05	2.900	Up
UBXN4	1.067	10.366	4.382	1.82E-05	0.000106422	2.502	Up
ACAN	1.071	8.315	4.316	2.39E-05	0.000131898	2.244	Up
PTPRO	1.903	11.908	4.244	3.23E-05	0.000170731	1.965	Up
LITAF	1.445	10.759	4.224	3.51E-05	0.000182543	1.886	Up
SLC11A1	1.171	8.358	4.044	7.25E-05	0.000331573	1.209	Up
SOX3	1.012	5.881	3.956	0.000102613	0.000444858	0.885	Up
CLU	1.227	10.016	3.857	0.000150588	0.000600332	0.529	Up
CRABP1	1.012	8.815	2.869	0.004511283	0.010997177	-2.576	Up
BBX	0.571	9.457	7.324	4.43E-12	2.20E-09	17.035	NoSig
G3BP1	0.860	10.222	7.251	6.85E-12	2.30E-09	16.616	NoSig
ZKSCAN3	-0.901	8.416	-7.200	9.29E-12	2.30E-09	16.323	NoSig
ZNF292	0.782	9.375	6.853	7.09E-11	1.01E-08	14.369	NoSig
DCTN5	-0.659	9.042	-6.810	9.10E-11	1.12E-08	14.129	NoSig
MCFD2	0.735	8.983	6.763	1.18E-10	1.26E-08	13.875	NoSig
CEP164	-0.975	8.782	-6.635	2.46E-10	2.28E-08	13.175	NoSig
TTN	-0.769	7.736	-6.535	4.32E-10	3.21E-08	12.633	NoSig
N4BP2L2	0.905	9.731	6.440	7.31E-10	4.73E-08	12.128	NoSig
SPG11	0.973	8.942	6.387	9.82E-10	5.81E-08	11.846	NoSig
BICD2	0.910	8.636	6.384	9.98E-10	5.81E-08	11.830	NoSig
GGNBP2	0.838	9.356	6.233	2.28E-09	1.13E-07	11.038	NoSig
CLTA	0.851	11.698	6.198	2.76E-09	1.32E-07	10.855	NoSig
ARMC8	-0.608	9.032	-6.159	3.42E-09	1.54E-07	10.651	NoSig
SMC3	0.696	9.067	6.146	3.66E-09	1.60E-07	10.586	NoSig
SLC38A10	0.909	9.175	6.132	3.95E-09	1.68E-07	10.514	NoSig
PTCH1	0.935	7.362	6.105	4.55E-09	1.80E-07	10.378	NoSig
RHOA	-0.630	13.246	-6.079	5.22E-09	1.91E-07	10.245	NoSig
DCAF8	-0.506	9.171	-6.059	5.84E-09	2.02E-07	10.138	NoSig
TXLNG	-0.954	7.314	-5.985	8.65E-09	2.71E-07	9.763	NoSig
BECN1	0.682	9.508	5.982	8.76E-09	2.71E-07	9.750	NoSig
NDUFS4	0.751	9.565	5.964	9.65E-09	2.81E-07	9.658	NoSig
MOCS1	-0.746	7.555	-5.939	1.10E-08	3.03E-07	9.532	NoSig
CKAP5	-0.591	10.041	-5.923	1.20E-08	3.17E-07	9.453	NoSig
CELF1	0.570	11.038	5.907	1.30E-08	3.40E-07	9.371	NoSig
DNAJA4	-0.547	9.593	-5.892	1.40E-08	3.48E-07	9.299	NoSig
PDHA1	-0.818	11.248	-5.879	1.50E-08	3.67E-07	9.234	NoSig
DNAJC2	0.954	9.783	5.853	1.73E-08	4.14E-07	9.102	NoSig

PALLD	0.830	10.842	5.843	1.82E-08	4.22E-07	9.053	NoSig
SUPT6H	-0.472	9.463	-5.840	1.85E-08	4.22E-07	9.038	NoSig
SUZ12	0.677	10.035	5.801	2.26E-08	5.10E-07	8.843	NoSig
CKAP2	0.772	8.853	5.784	2.46E-08	5.47E-07	8.763	NoSig
CCDC7	-0.726	7.809	-5.777	2.56E-08	5.60E-07	8.726	NoSig
CACNA1D	-0.791	7.546	-5.767	2.69E-08	5.80E-07	8.678	NoSig
POLR2B	-0.733	11.307	-5.759	2.80E-08	5.95E-07	8.640	NoSig
SRGAP3	-0.642	8.202	-5.705	3.71E-08	7.37E-07	8.372	NoSig
STXBP3	0.778	8.417	5.704	3.72E-08	7.37E-07	8.369	NoSig
C4orf27	0.674	9.349	5.697	3.86E-08	7.55E-07	8.334	NoSig
ZNF573	0.884	7.373	5.662	4.62E-08	8.81E-07	8.162	NoSig
PIK3R2	-0.697	8.284	-5.612	5.96E-08	1.12E-06	7.920	NoSig
HSD17B4	0.705	9.490	5.585	6.83E-08	1.27E-06	7.790	NoSig
CRYZL1	-0.717	8.177	-5.578	7.06E-08	1.30E-06	7.758	NoSig
KRAS	0.862	10.224	5.575	7.15E-08	1.30E-06	7.746	NoSig
SYF2	0.966	8.969	5.543	8.43E-08	1.49E-06	7.590	NoSig
PRR14L	-0.840	8.622	-5.540	8.54E-08	1.49E-06	7.577	NoSig
HNRNPM	-0.742	11.876	-5.512	9.85E-08	1.68E-06	7.441	NoSig
MRPS27	0.770	8.933	5.466	1.24E-07	2.02E-06	7.223	NoSig
PSIP1	0.834	9.581	5.466	1.24E-07	2.02E-06	7.222	NoSig
TGFB3	0.969	8.420	5.465	1.24E-07	2.02E-06	7.218	NoSig
CARS2	0.991	9.435	5.454	1.32E-07	2.08E-06	7.165	NoSig
ADCY7	0.805	8.082	5.437	1.42E-07	2.23E-06	7.090	NoSig
PLXNA1	0.700	8.948	5.426	1.51E-07	2.33E-06	7.037	NoSig
DDX42	-0.627	10.405	-5.422	1.54E-07	2.36E-06	7.015	NoSig
MLLT10	-0.520	8.761	-5.396	1.75E-07	2.57E-06	6.895	NoSig
LMF1	0.995	6.745	5.380	1.89E-07	2.76E-06	6.819	NoSig
CD80	-0.594	8.572	-5.377	1.92E-07	2.77E-06	6.806	NoSig
SPTLC1	0.675	10.238	5.369	2.00E-07	2.85E-06	6.769	NoSig
NDUFB6	0.960	10.442	5.356	2.13E-07	3.02E-06	6.706	NoSig
KIAA1109	-0.755	8.391	-5.347	2.22E-07	3.12E-06	6.667	NoSig
RAP2A	0.987	10.334	5.345	2.24E-07	3.12E-06	6.658	NoSig
CHERP	0.691	9.729	5.342	2.28E-07	3.14E-06	6.642	NoSig
IST1	0.556	10.684	5.330	2.42E-07	3.27E-06	6.585	NoSig
RSU1	0.891	10.193	5.316	2.59E-07	3.44E-06	6.522	NoSig
RHOQ	0.849	10.226	5.316	2.59E-07	3.44E-06	6.521	NoSig
ZNF286A	-0.756	7.875	-5.300	2.80E-07	3.65E-06	6.447	NoSig
FSTL4	-0.618	5.855	-5.269	3.25E-07	4.14E-06	6.304	NoSig
GOLGA3	0.599	9.004	5.267	3.28E-07	4.14E-06	6.296	NoSig
PHC3	-0.772	7.215	-5.252	3.53E-07	4.38E-06	6.227	NoSig
GEMIN7	-0.753	7.288	-5.251	3.54E-07	4.38E-06	6.225	NoSig
TP73	-0.871	4.189	-5.237	3.79E-07	4.65E-06	6.160	NoSig
ZNF7	0.707	8.448	5.221	4.10E-07	4.97E-06	6.085	NoSig
C11orf58	0.837	10.933	5.220	4.11E-07	4.97E-06	6.082	NoSig
CNKSR2	-0.953	6.546	-5.210	4.31E-07	5.17E-06	6.036	NoSig

RPS24	0.943	14.428	5.202	4.49E-07	5.32E-06	5.998	NoSig
EDIL3	-0.746	6.812	-5.191	4.72E-07	5.52E-06	5.951	NoSig
MCTS1	0.661	9.930	5.188	4.80E-07	5.58E-06	5.934	NoSig
TLR4	-0.581	8.215	-5.182	4.93E-07	5.68E-06	5.909	NoSig
LIG3	-0.754	8.434	-5.172	5.17E-07	5.91E-06	5.864	NoSig
DDX3X	0.565	11.351	5.144	5.91E-07	6.51E-06	5.737	NoSig
SERPINC1	-0.702	4.050	-5.136	6.15E-07	6.71E-06	5.700	NoSig
PARP11	-0.951	6.273	-5.135	6.18E-07	6.71E-06	5.695	NoSig
HEXA	0.726	9.617	5.133	6.23E-07	6.71E-06	5.687	NoSig
C2	0.973	8.484	5.123	6.53E-07	6.99E-06	5.642	NoSig
PDS5B	0.545	8.846	5.118	6.70E-07	7.01E-06	5.618	NoSig
GTF3C3	0.604	8.636	5.118	6.70E-07	7.01E-06	5.618	NoSig
SQLE	0.722	10.396	5.116	6.75E-07	7.01E-06	5.612	NoSig
GSTCD	-0.680	7.488	-5.109	6.97E-07	7.19E-06	5.581	NoSig
CSNK1A1	0.483	11.439	5.098	7.35E-07	7.48E-06	5.531	NoSig
MACF1	0.641	10.452	5.090	7.65E-07	7.74E-06	5.493	NoSig
CLCNKB	-0.841	5.048	-5.061	8.77E-07	8.68E-06	5.363	NoSig
KIAA0226	0.397	8.923	5.060	8.82E-07	8.68E-06	5.358	NoSig
MFN2	0.767	9.555	5.051	9.19E-07	8.97E-06	5.318	NoSig
CACNB2	-0.610	7.228	-5.050	9.23E-07	8.97E-06	5.315	NoSig
ATM	-0.557	9.117	-5.041	9.63E-07	9.24E-06	5.274	NoSig
SLC4A1AP	-0.411	9.429	-5.041	9.63E-07	9.24E-06	5.274	NoSig
IPP	-0.689	7.920	-5.029	1.02E-06	9.62E-06	5.223	NoSig
ZMYM3	-0.421	9.377	-5.002	1.16E-06	1.08E-05	5.100	NoSig
PIEZO1	0.971	9.417	5.001	1.16E-06	1.08E-05	5.100	NoSig
RQCD1	-0.337	9.983	-4.998	1.18E-06	1.09E-05	5.084	NoSig
ZNF75D	-0.471	8.046	-4.957	1.42E-06	1.29E-05	4.904	NoSig
CEP70	-0.583	8.617	-4.956	1.43E-06	1.29E-05	4.899	NoSig
JRKL	0.846	7.803	4.953	1.45E-06	1.30E-05	4.889	NoSig
LPAR6	0.721	8.748	4.950	1.47E-06	1.31E-05	4.875	NoSig
GDAP2	-0.371	8.085	-4.948	1.48E-06	1.31E-05	4.866	NoSig
LUC7L	-0.794	8.713	-4.933	1.59E-06	1.39E-05	4.799	NoSig
SNRPB2	0.886	10.385	4.920	1.69E-06	1.46E-05	4.741	NoSig
DDX10	0.679	9.262	4.916	1.72E-06	1.48E-05	4.724	NoSig
PLXND1	0.768	9.906	4.915	1.73E-06	1.48E-05	4.720	NoSig
AVL9	0.666	8.732	4.904	1.82E-06	1.54E-05	4.673	NoSig
PERP	0.951	10.542	4.876	2.07E-06	1.73E-05	4.550	NoSig
PSMD6	0.632	10.776	4.861	2.21E-06	1.82E-05	4.488	NoSig
VPS35	0.804	11.249	4.840	2.43E-06	2.00E-05	4.397	NoSig
SREBF1	0.690	9.137	4.833	2.51E-06	2.05E-05	4.368	NoSig
EFCAB6	-0.888	4.314	-4.823	2.63E-06	2.14E-05	4.323	NoSig
SMCHD1	-0.716	9.364	-4.819	2.67E-06	2.16E-05	4.308	NoSig
ATP5O	0.867	11.788	4.816	2.72E-06	2.19E-05	4.292	NoSig
PDE8A	-0.439	8.943	-4.811	2.78E-06	2.22E-05	4.270	NoSig
LPP	0.535	10.227	4.803	2.88E-06	2.29E-05	4.237	NoSig

MGA	0.890	6.658	4.800	2.92E-06	2.30E-05	4.223	NoSig
TOR1A	0.765	8.732	4.798	2.94E-06	2.30E-05	4.218	NoSig
RTN4	0.583	12.325	4.796	2.97E-06	2.31E-05	4.210	NoSig
HSPA6	-0.768	9.429	-4.792	3.03E-06	2.35E-05	4.189	NoSig
ARL3	0.648	8.931	4.779	3.21E-06	2.48E-05	4.134	NoSig
CTBP1	0.526	10.800	4.770	3.34E-06	2.56E-05	4.097	NoSig
ATP5H	0.754	12.044	4.742	3.79E-06	2.89E-05	3.978	NoSig
DNTT	-0.662	4.182	-4.733	3.95E-06	2.98E-05	3.939	NoSig
MRPL23	0.822	9.087	4.728	4.03E-06	3.03E-05	3.919	NoSig
SRRM2	-0.757	11.802	-4.724	4.11E-06	3.07E-05	3.901	NoSig
MKL1	-0.405	8.552	-4.718	4.22E-06	3.14E-05	3.876	NoSig
AFG3L2	-0.487	10.050	-4.715	4.28E-06	3.17E-05	3.864	NoSig
FGF1	-0.442	7.729	-4.707	4.43E-06	3.26E-05	3.832	NoSig
HS3ST3B1	-0.774	6.818	-4.699	4.60E-06	3.37E-05	3.796	NoSig
DIP2A	-0.670	9.013	-4.685	4.89E-06	3.56E-05	3.738	NoSig
ARPC5	-0.323	12.204	-4.676	5.08E-06	3.68E-05	3.702	NoSig
SH2D3C	-0.444	7.402	-4.671	5.20E-06	3.75E-05	3.680	NoSig
JMJD6	0.968	8.772	4.645	5.82E-06	4.18E-05	3.574	NoSig
CCRN4L	-0.403	8.601	-4.636	6.07E-06	4.31E-05	3.534	NoSig
CLSPN	-0.873	5.779	-4.635	6.08E-06	4.31E-05	3.532	NoSig
SOX4	0.822	11.581	4.631	6.20E-06	4.35E-05	3.515	NoSig
ORAI2	-0.489	8.145	-4.628	6.28E-06	4.39E-05	3.501	NoSig
TFB2M	0.761	9.245	4.615	6.66E-06	4.63E-05	3.447	NoSig
MEF2BNB	-0.864	7.261	-4.614	6.70E-06	4.63E-05	3.441	NoSig
NEK11	-0.369	8.792	-4.601	7.07E-06	4.87E-05	3.390	NoSig
PNN	-0.392	12.626	-4.588	7.48E-06	5.10E-05	3.337	NoSig
STX16	-0.460	10.554	-4.579	7.78E-06	5.28E-05	3.301	NoSig
MBTPS2	-0.556	7.666	-4.570	8.12E-06	5.49E-05	3.260	NoSig
WARS2	0.712	7.784	4.567	8.21E-06	5.53E-05	3.249	NoSig
TIA1	-0.464	10.520	-4.564	8.34E-06	5.58E-05	3.235	NoSig
ZSCAN5A	0.710	7.015	4.563	8.36E-06	5.58E-05	3.232	NoSig
MYNN	0.368	8.079	4.557	8.57E-06	5.69E-05	3.209	NoSig
CRTAP	-0.627	8.993	-4.551	8.80E-06	5.82E-05	3.184	NoSig
LAPTM4B	0.778	12.664	4.533	9.51E-06	6.25E-05	3.111	NoSig
SKIV2L2	0.446	9.190	4.532	9.54E-06	6.25E-05	3.108	NoSig
SART3	0.584	9.074	4.528	9.71E-06	6.33E-05	3.092	NoSig
ATP5SL	0.346	9.434	4.526	9.81E-06	6.37E-05	3.082	NoSig
SF3B2	0.779	9.533	4.517	1.02E-05	6.60E-05	3.044	NoSig
SMARCC2	0.662	8.467	4.503	1.08E-05	6.98E-05	2.988	NoSig
CYB5R3	0.500	11.545	4.501	1.09E-05	7.00E-05	2.981	NoSig
SLC38A4	-0.871	4.849	-4.496	1.12E-05	7.14E-05	2.959	NoSig
SNX13	0.361	8.906	4.492	1.13E-05	7.21E-05	2.945	NoSig
ASCC1	-0.574	8.839	-4.472	1.24E-05	7.79E-05	2.864	NoSig
TM2D1	0.424	9.068	4.467	1.27E-05	7.93E-05	2.842	NoSig
BRF1	-0.789	6.602	-4.466	1.27E-05	7.93E-05	2.840	NoSig

PGK1	0.757	12.543	4.465	1.28E-05	7.94E-05	2.835	NoSig
CDKL1	-0.947	4.133	-4.447	1.38E-05	8.53E-05	2.764	NoSig
MAGI1	-0.508	8.302	-4.444	1.40E-05	8.61E-05	2.751	NoSig
NOC2L	0.576	8.692	4.440	1.42E-05	8.71E-05	2.736	NoSig
HUNK	-0.605	7.055	-4.439	1.43E-05	8.72E-05	2.731	NoSig
KIAA1107	-0.491	6.869	-4.432	1.47E-05	8.94E-05	2.704	NoSig
SRPK2	-0.494	9.918	-4.425	1.51E-05	9.16E-05	2.674	NoSig
KDELR1	-0.832	9.517	-4.425	1.51E-05	9.16E-05	2.674	NoSig
TMEM135	0.734	7.445	4.416	1.57E-05	9.42E-05	2.639	NoSig
ATP11B	-0.802	8.786	-4.414	1.59E-05	9.47E-05	2.631	NoSig
GATM	0.950	7.522	4.413	1.59E-05	9.47E-05	2.626	NoSig
GOLM1	0.901	8.419	4.410	1.62E-05	9.57E-05	2.613	NoSig
PAK6	-0.602	7.752	-4.401	1.68E-05	9.91E-05	2.577	NoSig
JRK	-0.415	9.076	-4.392	1.74E-05	0.000102352	2.543	NoSig
ATP5S	-0.356	8.314	-4.379	1.84E-05	0.000106842	2.492	NoSig
SNX19	-0.806	7.954	-4.379	1.84E-05	0.000106842	2.491	NoSig
YEATS2	0.395	9.097	4.375	1.87E-05	0.000108356	2.474	NoSig
MAP3K11	0.735	9.313	4.363	1.96E-05	0.000113236	2.429	NoSig
METTL17	0.933	9.303	4.358	2.01E-05	0.000115015	2.410	NoSig
CAND1	0.614	8.954	4.355	2.03E-05	0.000115833	2.397	NoSig
TRIM44	0.531	9.773	4.353	2.05E-05	0.000116455	2.389	NoSig
EYA3	-0.984	5.810	-4.334	2.22E-05	0.000125172	2.314	NoSig
AGFG2	-0.554	7.627	-4.324	2.32E-05	0.000129707	2.275	NoSig
PDGFRA	0.962	7.500	4.324	2.32E-05	0.000129707	2.274	NoSig
CNNM2	-0.477	8.512	-4.319	2.37E-05	0.000131765	2.255	NoSig
PRMT1	0.910	10.657	4.317	2.39E-05	0.000131898	2.247	NoSig
VPS53	-0.478	7.370	-4.317	2.39E-05	0.000131898	2.246	NoSig
ERC1	-0.328	9.071	-4.309	2.47E-05	0.000135227	2.214	NoSig
SCN8A	-0.699	3.811	-4.308	2.47E-05	0.000135227	2.214	NoSig
TXNDC9	0.357	10.426	4.280	2.79E-05	0.000151745	2.102	NoSig
FAM184A	-0.520	8.099	-4.278	2.81E-05	0.000152545	2.094	NoSig
TEX10	-0.298	9.679	-4.272	2.88E-05	0.000155554	2.072	NoSig
GPNMB	0.853	11.785	4.268	2.93E-05	0.000157863	2.055	NoSig
POFUT2	0.664	8.334	4.266	2.95E-05	0.000158521	2.048	NoSig
HSD17B12	0.688	11.148	4.263	2.98E-05	0.000159455	2.039	NoSig
TRIB3	0.672	9.403	4.252	3.12E-05	0.000166293	1.996	NoSig
BRD3	0.646	9.762	4.250	3.15E-05	0.00016752	1.986	NoSig
KAT6A	0.533	10.214	4.243	3.25E-05	0.000171321	1.958	NoSig
TIMM17B	0.974	8.876	4.240	3.28E-05	0.000172565	1.948	NoSig
ATN1	-0.376	9.000	-4.234	3.37E-05	0.000176191	1.925	NoSig
NLRX1	0.651	8.017	4.224	3.51E-05	0.000182543	1.886	NoSig
GON4L	-0.347	9.800	-4.222	3.53E-05	0.00018292	1.880	NoSig
FGFR1OP	-0.604	8.930	-4.219	3.59E-05	0.000185178	1.866	NoSig
TMEM126B	0.539	10.355	4.216	3.63E-05	0.000186823	1.854	NoSig
IKZF1	-0.474	9.216	-4.206	3.77E-05	0.000192749	1.818	NoSig

CARD8	-0.501	8.544	-4.205	3.78E-05	0.000192749	1.815	NoSig
ADAM21	-0.574	7.885	-4.195	3.94E-05	0.000199508	1.777	NoSig
FAM69A	-0.520	8.744	-4.192	3.99E-05	0.000200807	1.765	NoSig
MTHFSD	-0.643	7.819	-4.192	4.00E-05	0.000200807	1.764	NoSig
PMP22	0.812	9.962	4.182	4.16E-05	0.000208215	1.727	NoSig
GHITM	-0.374	11.738	-4.163	4.51E-05	0.000224954	1.652	NoSig
ZNF586	-0.412	8.424	-4.161	4.53E-05	0.000225297	1.647	NoSig
IDH1	-0.475	10.959	-4.155	4.64E-05	0.000229938	1.625	NoSig
CALCOCO2	0.480	9.676	4.147	4.80E-05	0.000236952	1.594	NoSig
TCEA1	0.684	11.442	4.143	4.88E-05	0.000240455	1.577	NoSig
TXNL4A	0.542	11.289	4.141	4.92E-05	0.000240972	1.571	NoSig
LMNA	0.484	11.225	4.141	4.93E-05	0.000240972	1.569	NoSig
NEK4	0.503	7.637	4.138	4.97E-05	0.000242548	1.560	NoSig
C2CD3	0.353	8.486	4.137	5.00E-05	0.000243139	1.554	NoSig
PMS1	0.411	9.328	4.132	5.11E-05	0.000247311	1.536	NoSig
HS1BP3	0.558	9.032	4.127	5.20E-05	0.000251038	1.519	NoSig
GPD1	-0.771	5.628	-4.120	5.36E-05	0.000258156	1.489	NoSig
SLC18A2	-0.917	4.643	-4.099	5.82E-05	0.000277405	1.413	NoSig
ANKMY1	-0.780	5.442	-4.096	5.89E-05	0.000279885	1.402	NoSig
HPRT1	0.852	10.226	4.095	5.93E-05	0.000280766	1.396	NoSig
MKL2	0.298	9.387	4.087	6.12E-05	0.000288717	1.367	NoSig
GTF2H5	0.501	9.767	4.083	6.21E-05	0.00029209	1.353	NoSig
HIVEP1	0.375	7.577	4.080	6.29E-05	0.000294136	1.341	NoSig
KLF7	-0.457	9.514	-4.078	6.35E-05	0.000295871	1.332	NoSig
HIP1	0.451	7.696	4.068	6.60E-05	0.000306736	1.296	NoSig
YTHDC2	-0.620	8.284	-4.059	6.83E-05	0.000316328	1.264	NoSig
CYB5R1	0.649	9.843	4.056	6.91E-05	0.000319179	1.253	NoSig
PGD	0.952	9.173	4.055	6.93E-05	0.000319179	1.250	NoSig
CLCC1	-0.609	9.307	-4.049	7.12E-05	0.000326573	1.226	NoSig
PLCG2	0.730	9.207	4.037	7.45E-05	0.000339963	1.183	NoSig
SCARB1	0.511	9.457	4.032	7.62E-05	0.000346299	1.163	NoSig
CCDC90B	0.321	9.591	4.019	8.01E-05	0.000362395	1.115	NoSig
OSBP	0.336	9.396	4.019	8.02E-05	0.000362395	1.115	NoSig
RGS12	-0.339	8.074	-4.017	8.07E-05	0.000363607	1.109	NoSig
RBM25	-0.431	9.991	-4.014	8.18E-05	0.000367639	1.096	NoSig
CNP	0.414	10.320	4.003	8.53E-05	0.000382135	1.057	NoSig
NOS1AP	-0.493	4.902	-4.001	8.59E-05	0.000383409	1.051	NoSig
ARHGEF2	0.623	10.397	3.999	8.68E-05	0.000386537	1.041	NoSig
INSL3	-0.455	7.190	-3.987	9.08E-05	0.000402957	0.999	NoSig
PIAS2	-0.424	8.872	-3.985	9.17E-05	0.000405953	0.989	NoSig
SCN1A	-0.899	4.164	-3.980	9.34E-05	0.000412141	0.973	NoSig
ATF2	0.535	7.708	3.974	9.58E-05	0.000421471	0.949	NoSig
RAD51B	-0.467	6.620	-3.967	9.83E-05	0.000429872	0.925	NoSig
AMN	-0.624	7.711	-3.959	0.000101317	0.000441812	0.897	NoSig
PIGG	0.357	9.342	3.957	0.000102231	0.000444495	0.889	NoSig

CAMKMT	-0.646	6.859	-3.952	0.000104272	0.000450735	0.870	NoSig
PAPOLG	0.510	7.120	3.946	0.000106672	0.000459774	0.849	NoSig
DNAJA3	-0.251	10.468	-3.937	0.000110621	0.000475415	0.815	NoSig
GGA1	0.716	11.256	3.933	0.000112102	0.000480227	0.803	NoSig
PRKD3	0.514	8.720	3.928	0.000114276	0.000486899	0.785	NoSig
RASGRF1	-0.476	7.574	-3.926	0.00011546	0.000490539	0.775	NoSig
TIMM10	-0.584	9.992	-3.920	0.000118182	0.000500676	0.754	NoSig
TGOLN2	0.328	10.626	3.916	0.000120013	0.000506989	0.739	NoSig
SLC26A1	0.713	7.031	3.907	0.00012426	0.000523443	0.707	NoSig
WAPAL	-0.529	9.068	-3.904	0.000125501	0.000527175	0.698	NoSig
NFX1	-0.358	9.280	-3.903	0.000126189	0.000528572	0.693	NoSig
HSP90AB1	-0.502	13.983	-3.896	0.00012952	0.000538346	0.669	NoSig
PHKG2	0.551	8.320	3.896	0.000129574	0.000538346	0.668	NoSig
C19orf26	-0.833	2.981	-3.895	0.000129847	0.000538346	0.666	NoSig
NKTR	0.383	10.307	3.895	0.000129971	0.000538346	0.665	NoSig
GSN	0.528	10.685	3.892	0.00013171	0.000544036	0.653	NoSig
RNASET2	0.772	11.060	3.890	0.000132592	0.00054616	0.647	NoSig
ATAD2B	-0.563	8.364	-3.877	0.000139239	0.000569792	0.601	NoSig
KLK8	0.827	8.550	3.877	0.000139478	0.000569792	0.600	NoSig
NEDD9	0.513	8.120	3.874	0.000141169	0.000575121	0.589	NoSig
MCOLN3	-0.902	5.738	-3.871	0.000142569	0.000579233	0.580	NoSig
MYO10	0.609	9.884	3.867	0.000144663	0.000586142	0.566	NoSig
ACP1	0.437	10.630	3.867	0.000145091	0.000586279	0.563	NoSig
SCARF1	-0.720	6.285	-3.865	0.000145928	0.000587767	0.558	NoSig
IRAK3	-0.960	5.594	-3.865	0.00014625	0.000587767	0.556	NoSig
ARHGAP19	0.724	6.238	3.862	0.000147439	0.000590948	0.548	NoSig
FBXO42	0.418	8.123	3.862	0.000147858	0.000591036	0.546	NoSig
VPS37C	-0.542	9.725	-3.854	0.000152438	0.000606084	0.517	NoSig
USP25	-0.260	10.027	-3.849	0.000154916	0.000614293	0.503	NoSig
ARIH1	0.396	9.317	3.827	0.000168869	0.000667841	0.423	NoSig
DCAF13	0.649	6.580	3.820	0.000173114	0.000682813	0.400	NoSig
SLC4A8	-0.893	4.666	-3.809	0.000180428	0.00070978	0.361	NoSig
PCNXL2	-0.469	8.235	-3.807	0.000181808	0.000713322	0.354	NoSig
PDZD3	-0.467	5.401	-3.802	0.000185551	0.00072609	0.335	NoSig
BICD1	-0.573	7.895	-3.792	0.000192494	0.000751281	0.301	NoSig
NAP1L4	-0.291	10.005	-3.792	0.000193081	0.000751601	0.298	NoSig
DYNLRB1	0.596	11.419	3.788	0.000195914	0.000760636	0.285	NoSig
STRAP	0.475	11.745	3.779	0.000202606	0.000782532	0.254	NoSig
DDX6	-0.508	7.911	-3.764	0.00021404	0.000824552	0.203	NoSig
SLC8A1	-0.857	6.958	-3.762	0.000215472	0.000827923	0.197	NoSig
FBXL18	-0.322	7.816	-3.760	0.00021722	0.000832488	0.189	NoSig
OSBPL1A	0.398	9.489	3.759	0.000218165	0.000833828	0.185	NoSig
NFE2L2	0.374	10.217	3.759	0.000218691	0.000833828	0.183	NoSig
THADA	-0.324	9.102	-3.758	0.00021927	0.000833899	0.181	NoSig
TSPAN3	0.506	10.262	3.748	0.000227661	0.0008636	0.146	NoSig

CRISPLD2	0.557	9.025	3.745	0.000230079	0.000870555	0.136	NoSig
P2RY10	-0.753	6.097	-3.740	0.000234261	0.000882795	0.119	NoSig
DGKE	-0.671	6.214	-3.740	0.000234502	0.000882795	0.119	NoSig
SETD6	-0.836	8.160	-3.734	0.000240164	0.000901828	0.096	NoSig
UBA1	0.677	11.492	3.733	0.000241001	0.000902692	0.093	NoSig
ETS1	-0.833	7.394	-3.731	0.000242841	0.000907296	0.086	NoSig
ANGPT1	0.644	8.391	3.727	0.000246221	0.000917619	0.073	NoSig
DNM3	-0.390	7.634	-3.721	0.000251267	0.000934084	0.055	NoSig
RNF32	-0.858	5.178	-3.718	0.000254553	0.000943939	0.043	NoSig
QTRTD1	0.565	7.253	3.716	0.000256205	0.000947705	0.037	NoSig
UBTF	-0.535	8.257	-3.715	0.000257216	0.000949084	0.033	NoSig
BAP1	0.449	8.868	3.713	0.000259052	0.000953492	0.026	NoSig
NOX5	-0.540	6.957	-3.706	0.000266031	0.000976485	0.002	NoSig
KCNIP2	-0.581	6.733	-3.705	0.000266613	0.000976485	0.000	NoSig
PAPPA	-0.533	6.645	-3.703	0.00026938	0.000984197	-0.010	NoSig
SSBP1	0.562	10.950	3.697	0.000275155	0.001002834	-0.029	NoSig
SREK1IP1	-0.912	7.170	-3.696	0.000276224	0.001004266	-0.033	NoSig
TP63	0.739	7.878	3.691	0.000281679	0.001021602	-0.051	NoSig
ASMTL.AS1	-0.857	3.415	-3.685	0.000287243	0.001039247	-0.069	NoSig
BCL2L13	0.319	8.867	3.675	0.000298516	0.001077409	-0.104	NoSig
ACAT1	0.442	10.235	3.671	0.000302537	0.001089278	-0.117	NoSig
IBA57	-0.745	6.114	-3.667	0.000306969	0.001102567	-0.130	NoSig
LRPPRC	0.409	10.936	3.662	0.000313309	0.001122626	-0.149	NoSig
ABHD11	0.514	9.800	3.660	0.000315577	0.001128035	-0.156	NoSig
CMAHP	0.503	7.783	3.659	0.000316348	0.001128082	-0.158	NoSig
SHB	-0.425	8.800	-3.658	0.000317736	0.001130321	-0.162	NoSig
ARHGEF12	0.455	9.302	3.644	0.000334969	0.001188781	-0.211	NoSig
PDZRN3	0.770	7.026	3.639	0.000341268	0.001208251	-0.228	NoSig
RABGAP1L	0.519	9.235	3.635	0.000345318	0.001219687	-0.239	NoSig
CAST	0.334	10.182	3.630	0.000352308	0.001241427	-0.257	NoSig
PPIP5K1	-0.639	6.359	-3.626	0.000357311	0.001255495	-0.270	NoSig
CYTH2	-0.437	8.864	-3.625	0.000357989	0.001255495	-0.272	NoSig
EXD2	-0.312	9.021	-3.617	0.00036969	0.00129348	-0.302	NoSig
CCDC57	-0.749	5.618	-3.606	0.00038485	0.001343362	-0.339	NoSig
PTPRE	0.523	8.657	3.603	0.000388081	0.001351468	-0.346	NoSig
C5orf22	-0.403	9.424	-3.589	0.000408837	0.001419658	-0.394	NoSig
CCNL1	-0.452	10.494	-3.588	0.000409572	0.001419658	-0.396	NoSig
NDUFA7	0.775	8.522	3.579	0.000423335	0.001463953	-0.426	NoSig
TBL3	0.605	7.545	3.571	0.00043547	0.001502423	-0.452	NoSig
DNAJC7	-0.344	9.482	-3.565	0.0004464	0.001536565	-0.475	NoSig
NAP1L1	0.398	11.783	3.553	0.000465877	0.001599906	-0.515	NoSig
DYNC2H1	-0.352	7.607	-3.535	0.000495774	0.001698655	-0.572	NoSig
HGSNAT	0.451	9.210	3.530	0.00050608	0.00172998	-0.591	NoSig
BCL2L11	-0.644	7.235	-3.526	0.000512252	0.001747063	-0.602	NoSig
SLC9A7	-0.920	5.551	-3.522	0.000519572	0.00176797	-0.615	NoSig

MAK	-0.728	5.240	-3.519	0.000525754	0.001784924	-0.626	NoSig
MEG3	0.634	6.761	3.511	0.000540032	0.001829221	-0.650	NoSig
SNAPC5	-0.399	9.494	-3.507	0.000548894	0.001855012	-0.665	NoSig
DPY19L4	0.338	8.733	3.494	0.000575473	0.001940428	-0.709	NoSig
ARHGAP25	0.629	8.768	3.486	0.000590831	0.001987705	-0.733	NoSig
ERBB3	0.492	9.885	3.483	0.000597288	0.002000274	-0.743	NoSig
NBR2	-0.918	5.279	-3.483	0.000597505	0.002000274	-0.743	NoSig
SLC16A1	-0.442	9.232	-3.482	0.000598602	0.002000274	-0.745	NoSig
COPA	0.758	11.245	3.476	0.000612933	0.002043567	-0.766	NoSig
SUOX	-0.452	8.597	-3.475	0.000614521	0.002044278	-0.769	NoSig
PDE8B	-0.381	8.038	-3.474	0.000617238	0.002048736	-0.773	NoSig
SRC	-0.532	6.086	-3.471	0.000623662	0.002065446	-0.782	NoSig
HEATR6	-0.393	7.513	-3.467	0.000632063	0.002088618	-0.795	NoSig
CNDP2	0.527	10.634	3.462	0.000643255	0.002120887	-0.811	NoSig
TSPYL1	0.375	9.632	3.453	0.000663352	0.00218231	-0.839	NoSig
POLH	-0.858	6.460	-3.451	0.000668726	0.002195135	-0.846	NoSig
DOCK4	-0.456	8.048	-3.444	0.000684838	0.00224307	-0.868	NoSig
XCR1	-0.537	5.212	-3.444	0.000686411	0.002243283	-0.870	NoSig
JPH2	-0.687	3.717	-3.441	0.000691546	0.002255106	-0.877	NoSig
RFX4	-0.658	3.620	-3.439	0.000696863	0.002267474	-0.884	NoSig
ABCC5	0.459	9.622	3.438	0.000698842	0.002268949	-0.887	NoSig
TTLL4	-0.300	8.508	-3.435	0.000706884	0.002288239	-0.897	NoSig
DGKH	-0.488	7.790	-3.435	0.000707861	0.002288239	-0.898	NoSig
PIK3C2A	-0.614	9.118	-3.413	0.00076379	0.002463679	-0.968	NoSig
HSDL2	0.537	7.891	3.410	0.00077076	0.002480779	-0.976	NoSig
SYNE2	-0.440	9.383	-3.407	0.00078023	0.002505837	-0.988	NoSig
TOR1AIP1	0.503	9.710	3.406	0.000783604	0.002511247	-0.992	NoSig
PIGK	0.344	8.236	3.404	0.000788999	0.0025231	-0.998	NoSig
CEP68	-0.261	8.855	-3.402	0.000794162	0.00253416	-1.004	NoSig
CLIP1	-0.659	8.877	-3.396	0.00081091	0.002581786	-1.023	NoSig
ZBTB24	-0.434	8.855	-3.395	0.000812559	0.002581786	-1.025	NoSig
PQLC2	-0.385	7.842	-3.389	0.000829809	0.002628313	-1.044	NoSig
TLL1	-0.535	5.565	-3.389	0.000830738	0.002628313	-1.045	NoSig
DNAH6	-0.339	6.005	-3.368	0.000891651	0.002815041	-1.110	NoSig
NAA16	-0.336	8.284	-3.366	0.00089982	0.002831132	-1.118	NoSig
DNAJC11	-0.208	9.400	-3.365	0.000900913	0.002831132	-1.119	NoSig
RNF103	0.386	9.720	3.365	0.000902459	0.002831132	-1.121	NoSig
SCPEP1	0.370	10.111	3.364	0.000906113	0.002836611	-1.124	NoSig
IRAK1	0.625	11.583	3.358	0.000923701	0.002885596	-1.142	NoSig
MOXD1	-0.408	8.318	-3.356	0.000931233	0.002903028	-1.149	NoSig
PRKAB2	-0.286	8.663	-3.354	0.000937235	0.002915624	-1.155	NoSig
SPEN	-0.222	9.826	-3.345	0.000966604	0.002995813	-1.183	NoSig
DSE	0.525	8.696	3.344	0.000969056	0.002995813	-1.186	NoSig
TRIM52	-0.543	7.850	-3.343	0.000971482	0.002997082	-1.188	NoSig
CYP2U1	-0.664	4.088	-3.340	0.000983777	0.003028728	-1.199	NoSig

NOS1	0.805	7.840	3.336	0.000997186	0.003063287	-1.212	NoSig
NCOA6	0.262	9.806	3.335	0.001000466	0.003063287	-1.215	NoSig
FBXO28	-0.286	9.686	-3.335	0.001001182	0.003063287	-1.215	NoSig
PCCB	0.518	9.341	3.331	0.001013272	0.003093913	-1.226	NoSig
CDK8	-0.422	7.903	-3.326	0.001032894	0.003147362	-1.244	NoSig
MAP4K4	-0.338	8.724	-3.319	0.001055089	0.00320842	-1.263	NoSig
TMBIM4	0.325	9.832	3.314	0.00107507	0.003260781	-1.280	NoSig
PTK7	0.520	8.727	3.313	0.001076694	0.003260781	-1.282	NoSig
MAST4	0.496	7.991	3.291	0.001161716	0.00351112	-1.351	NoSig
GABRG2	0.651	6.346	3.288	0.00117192	0.003527621	-1.359	NoSig
TAF5L	-0.354	9.470	-3.286	0.001180656	0.003546737	-1.366	NoSig
CNOT7	0.379	10.799	3.283	0.001195242	0.003583315	-1.377	NoSig
FCRL2	-0.547	7.053	-3.278	0.001213815	0.003631677	-1.391	NoSig
CFL1	0.620	14.723	3.276	0.001220129	0.003643236	-1.396	NoSig
MLXIP	0.351	9.269	3.275	0.001224398	0.003648657	-1.399	NoSig
DGKB	-0.732	4.019	-3.274	0.001229043	0.003655174	-1.402	NoSig
HPS4	-0.560	7.574	-3.272	0.0012408	0.003682773	-1.411	NoSig
ZNF264	0.475	8.534	3.270	0.001247378	0.003694923	-1.416	NoSig
ZMYM6	-0.468	8.281	-3.267	0.001260762	0.003727144	-1.426	NoSig
ZNF107	-0.616	7.911	-3.260	0.001291602	0.003810737	-1.448	NoSig
TAGLN	0.871	10.038	3.256	0.00130657	0.003847268	-1.458	NoSig
SYMPK	-0.727	6.952	-3.255	0.001311058	0.003852851	-1.461	NoSig
ST18	-0.764	3.639	-3.244	0.00136098	0.003991672	-1.495	NoSig
UCHL1	0.827	9.462	3.241	0.001372687	0.004018082	-1.503	NoSig
P4HB	0.363	12.989	3.237	0.001393526	0.004071067	-1.517	NoSig
WTAP	0.432	9.696	3.234	0.00140589	0.004099134	-1.525	NoSig
OSGIN2	-0.400	8.097	-3.231	0.001422868	0.004140517	-1.536	NoSig
WDR78	-0.299	7.298	-3.230	0.001425804	0.004140959	-1.537	NoSig
SRGN	0.709	11.284	3.229	0.001431427	0.004149186	-1.541	NoSig
WDR37	0.295	8.989	3.224	0.001452773	0.004202865	-1.554	NoSig
CYP2E1	-0.548	6.311	-3.217	0.001490229	0.004302857	-1.578	NoSig
HFE	-0.361	8.725	-3.204	0.001554108	0.004478602	-1.616	NoSig
ENTPD5	-0.404	7.713	-3.195	0.001602571	0.004609328	-1.644	NoSig
UBE3C	-0.286	9.545	-3.192	0.001619331	0.004648543	-1.653	NoSig
PSEN1	-0.254	9.433	-3.191	0.001625373	0.004656896	-1.656	NoSig
RBM8A	0.378	10.104	3.179	0.001690961	0.004835497	-1.692	NoSig
HINT1	-0.331	12.865	-3.175	0.001710188	0.004881093	-1.703	NoSig
GRK5	0.491	8.382	3.169	0.001743795	0.004967478	-1.720	NoSig
AGK	0.543	8.779	3.169	0.001748056	0.004970094	-1.722	NoSig
TNRC6B	-0.470	8.004	-3.166	0.001761857	0.004998688	-1.729	NoSig
KIAA1731	-0.949	4.300	-3.166	0.001764836	0.004998688	-1.731	NoSig
TSGA10	-0.541	6.474	-3.164	0.001773139	0.005012658	-1.735	NoSig
RABEPK	0.429	8.670	3.161	0.001792842	0.005058739	-1.745	NoSig
ELL	-0.451	7.411	-3.158	0.001810259	0.005090878	-1.754	NoSig
HLA.F.AS1	0.432	8.266	3.158	0.001811079	0.005090878	-1.754	NoSig

GAB1	-0.458	7.138	-3.154	0.001832147	0.005140383	-1.765	NoSig
ATG2B	-0.519	7.386	-3.147	0.001874655	0.005249739	-1.786	NoSig
SMTN	0.500	8.333	3.147	0.001879076	0.00525223	-1.788	NoSig
SLC39A14	0.482	9.484	3.143	0.001901291	0.005304352	-1.798	NoSig
GATA1	-0.524	6.633	-3.141	0.001913211	0.005327611	-1.804	NoSig
DDX50	0.380	9.745	3.122	0.002034882	0.00565583	-1.860	NoSig
QKI	-0.439	10.864	-3.118	0.0020607	0.005716904	-1.871	NoSig
SNED1	-0.434	7.534	-3.107	0.002139068	0.005923267	-1.905	NoSig
C5orf42	-0.883	5.455	-3.096	0.002214043	0.006119484	-1.936	NoSig
C1S	0.861	10.774	3.089	0.002267435	0.006255429	-1.958	NoSig
APOL6	-0.468	9.178	-3.087	0.002277972	0.00627286	-1.962	NoSig
TPR	-0.294	11.386	-3.086	0.002287744	0.006288123	-1.966	NoSig
PGLS	0.333	9.649	3.084	0.002299144	0.006297471	-1.970	NoSig
MYOZ3	-0.419	7.008	-3.084	0.002299615	0.006297471	-1.970	NoSig
BEND5	-0.318	7.142	-3.078	0.002346965	0.006415325	-1.989	NoSig
EAF2	0.497	7.436	3.076	0.002361317	0.006442712	-1.994	NoSig
IL12RB1	-0.523	5.745	-3.071	0.00239888	0.006527226	-2.009	NoSig
ST7L	-0.642	6.080	-3.071	0.002401071	0.006527226	-2.009	NoSig
THRA	-0.313	8.114	-3.066	0.002439128	0.006614145	-2.024	NoSig
TMEM144	-0.510	5.487	-3.066	0.00244194	0.006614145	-2.025	NoSig
PDE4D	-0.525	7.266	-3.064	0.002454876	0.006637093	-2.029	NoSig
IKZF2	-0.629	6.959	-3.057	0.00250824	0.006769062	-2.049	NoSig
RAPGEF6	-0.464	8.052	-3.050	0.002565046	0.006909825	-2.069	NoSig
SP1	-0.829	7.067	-3.047	0.002589821	0.006963949	-2.078	NoSig
TM2D3	-0.371	10.739	-3.045	0.002605512	0.006993495	-2.083	NoSig
GPR173	-0.523	6.055	-3.043	0.002629073	0.007031095	-2.091	NoSig
ZNF277	-0.373	8.558	-3.042	0.002632841	0.007031095	-2.093	NoSig
POF1B	-0.745	5.274	-3.041	0.002643122	0.007031095	-2.096	NoSig
EPHB3	0.565	9.699	3.041	0.0026456	0.007031095	-2.097	NoSig
ACACB	-0.554	7.518	-3.040	0.002648037	0.007031095	-2.098	NoSig
CCDC144A	-0.779	4.544	-3.040	0.002650269	0.007031095	-2.099	NoSig
RBL1	-0.436	6.789	-3.040	0.002652619	0.007031095	-2.099	NoSig
ARHGAP5	-0.300	8.510	-3.036	0.002688566	0.007112332	-2.111	NoSig
IGSF3	0.331	9.827	3.035	0.002692833	0.007112332	-2.113	NoSig
PECAM1	0.377	9.675	3.034	0.002703613	0.007128142	-2.116	NoSig
TPH1	-0.723	3.803	-3.030	0.002738261	0.007184599	-2.128	NoSig
CDON	-0.632	4.368	-3.030	0.002738416	0.007184599	-2.128	NoSig
CXorf36	-0.473	5.391	-3.030	0.002739521	0.007184599	-2.128	NoSig
SERPINB5	0.923	8.211	3.026	0.002767208	0.007244434	-2.137	NoSig
RAF1	-0.196	10.276	-3.024	0.002787874	0.007285708	-2.144	NoSig
MDFIC	0.477	9.518	3.017	0.002852247	0.007438363	-2.165	NoSig
ADAM23	0.504	7.336	3.016	0.002856292	0.007438363	-2.166	NoSig
CLEC7A	-0.597	9.423	-3.012	0.00289373	0.007522686	-2.178	NoSig
TTC37	-0.320	8.371	-3.010	0.002911143	0.007554746	-2.183	NoSig
KIAA0513	0.417	7.688	3.010	0.002916398	0.007555198	-2.185	NoSig

CSAD	-0.394	7.966	-3.008	0.002930147	0.00757479	-2.189	NoSig
MYLK	0.754	8.878	3.008	0.002934149	0.00757479	-2.190	NoSig
NAMPT	-0.453	10.415	-3.003	0.002977979	0.007674619	-2.203	NoSig
SNTG1	0.566	6.512	3.001	0.002999733	0.007717305	-2.210	NoSig
F11	0.695	6.200	2.999	0.003018672	0.007752617	-2.216	NoSig
ELMO2	0.287	7.927	2.998	0.003024108	0.007753186	-2.217	NoSig
MCF2	-0.593	5.626	-2.991	0.003098498	0.007930234	-2.239	NoSig
WWP2	-0.346	9.295	-2.988	0.00312955	0.007995945	-2.248	NoSig
MAF	0.388	8.136	2.979	0.003214312	0.008184387	-2.272	NoSig
ST8SIA4	-0.519	6.972	-2.975	0.003256852	0.008278528	-2.284	NoSig
YTHDF3	-0.410	9.534	-2.973	0.003270775	0.008299732	-2.288	NoSig
TNNT2	0.540	5.980	2.959	0.003418659	0.008660215	-2.327	NoSig
ZNF85	-0.262	9.392	-2.953	0.003489609	0.0088205	-2.346	NoSig
NPAS3	0.651	6.727	2.952	0.003493796	0.0088205	-2.347	NoSig
TTBK2	-0.452	6.625	-2.944	0.003583747	0.009032258	-2.370	NoSig
CCDC88A	0.495	7.015	2.927	0.003776925	0.009503025	-2.417	NoSig
NMNAT2	-0.587	6.355	-2.921	0.003848548	0.009666877	-2.434	NoSig
UBE2I	0.371	11.091	2.919	0.003875369	0.009717832	-2.440	NoSig
SLC2A4RG	-0.520	9.360	-2.915	0.003925999	0.009828216	-2.452	NoSig
CAPRIN2	-0.296	8.764	-2.914	0.003939439	0.009845286	-2.455	NoSig
PLA2G4C	0.479	7.742	2.911	0.003965706	0.009894303	-2.461	NoSig
ACTR2	-0.356	11.988	-2.909	0.003992644	0.009944826	-2.467	NoSig
CLASP2	-0.349	8.382	-2.905	0.004048732	0.010067667	-2.479	NoSig
ADAM9	-0.519	10.039	-2.891	0.004227439	0.010494493	-2.518	NoSig
DYRK3	0.469	7.601	2.887	0.004269786	0.010581954	-2.527	NoSig
RERE	0.392	9.454	2.885	0.004299663	0.010638267	-2.533	NoSig
PSMD10	-0.293	10.389	-2.882	0.00433674	0.010695344	-2.541	NoSig
GALNT1	-0.303	11.187	-2.882	0.004337117	0.010695344	-2.541	NoSig
WFDC2	0.602	8.970	2.877	0.004406468	0.010848375	-2.555	NoSig
PRMT5	-0.492	9.185	-2.875	0.004432128	0.010891424	-2.560	NoSig
VASH1	0.332	8.035	2.874	0.004444237	0.010891424	-2.562	NoSig
MKNK1	0.299	9.505	2.874	0.004445928	0.010891424	-2.563	NoSig
CFI	-0.544	8.202	-2.873	0.004462731	0.010914607	-2.566	NoSig
SLC9A5	-0.482	6.446	-2.871	0.004489864	0.010962937	-2.572	NoSig
MAGIX	-0.647	5.184	-2.869	0.004520479	0.011001559	-2.578	NoSig
KLHL35	-0.399	7.351	-2.867	0.004542398	0.011009187	-2.582	NoSig
BCL10	0.403	8.917	2.867	0.004542701	0.011009187	-2.582	NoSig
ALB	-0.601	5.800	-2.867	0.004545824	0.011009187	-2.583	NoSig
RBMS3	0.492	7.757	2.854	0.004732742	0.011443232	-2.619	NoSig
CLDN11	0.450	7.484	2.853	0.004743726	0.011451169	-2.621	NoSig
CBX3	0.237	11.968	2.851	0.004771481	0.011499502	-2.626	NoSig
BTG1	0.404	10.968	2.844	0.004870222	0.011712266	-2.644	NoSig
ADAMTS9	-0.218	8.585	-2.844	0.004875516	0.011712266	-2.645	NoSig
OSMR	-0.360	7.729	-2.843	0.004884151	0.011714085	-2.647	NoSig
JAK2	-0.334	8.085	-2.835	0.005009322	0.011994947	-2.669	NoSig

ITGA9	-0.457	7.058	-2.828	0.005112333	0.012221928	-2.687	NoSig
CELF2	0.435	9.867	2.814	0.005336964	0.012694583	-2.726	NoSig
IQCG	-0.399	9.138	-2.814	0.005337915	0.012694583	-2.726	NoSig
ALDH1A3	0.810	7.689	2.813	0.005345936	0.012694583	-2.727	NoSig
POLR1B	0.527	9.098	2.813	0.005346214	0.012694583	-2.727	NoSig
NAGA	-0.227	9.301	-2.813	0.005352726	0.012694583	-2.728	NoSig
ULK2	-0.262	7.807	-2.800	0.00556532	0.013177757	-2.763	NoSig
MAP3K2	-0.256	8.003	-2.792	0.005698156	0.013470839	-2.784	NoSig
EXOSC10	0.302	8.443	2.791	0.005708162	0.013473073	-2.786	NoSig
PTCD2	-0.523	5.593	-2.784	0.005838406	0.013740722	-2.806	NoSig
CBY1	0.347	9.267	2.784	0.005840038	0.013740722	-2.806	NoSig
SERPINB8	0.347	7.422	2.783	0.005853176	0.013749879	-2.808	NoSig
ZNF254	-0.669	6.093	-2.772	0.006037254	0.014159931	-2.835	NoSig
ZNF41	-0.649	5.093	-2.770	0.006088004	0.014256475	-2.843	NoSig
PRR5L	-0.532	6.423	-2.767	0.006128831	0.014329514	-2.849	NoSig
LARP4	-0.268	9.665	-2.760	0.006269238	0.014630104	-2.869	NoSig
VASH2	0.655	7.224	2.759	0.006277072	0.014630104	-2.870	NoSig
PER3	-0.413	7.877	-2.756	0.006338749	0.014750735	-2.879	NoSig
MAP3K6	0.557	7.506	2.754	0.006378856	0.014820873	-2.884	NoSig
ACSL4	-0.613	6.929	-2.749	0.006468117	0.015004821	-2.897	NoSig
TMC7	-0.450	6.071	-2.748	0.006492194	0.015037215	-2.900	NoSig
TMEM151B	-0.328	7.446	-2.746	0.006519725	0.015077499	-2.904	NoSig
LRCH3	-0.306	8.004	-2.745	0.006556657	0.015139362	-2.909	NoSig
LYZ	-0.613	11.957	-2.733	0.006784763	0.015641771	-2.939	NoSig
CPEB3	-0.708	5.223	-2.716	0.007122647	0.016395319	-2.982	NoSig
NT5DC2	0.522	9.004	2.712	0.007220046	0.01659383	-2.994	NoSig
VSTM4	-0.496	4.154	-2.709	0.007278632	0.016702663	-3.001	NoSig
ZNF146	-0.290	10.661	-2.704	0.007384636	0.016919806	-3.014	NoSig
PDXK	0.359	10.585	2.702	0.007427214	0.016991181	-3.019	NoSig
RIN3	0.413	8.404	2.701	0.007439909	0.016994078	-3.021	NoSig
SLC17A4	0.700	6.585	2.696	0.007546215	0.017210462	-3.033	NoSig
PDE1C	-0.401	7.895	-2.693	0.007619532	0.017351063	-3.042	NoSig
WDR19	0.320	8.143	2.690	0.007684218	0.017471609	-3.049	NoSig
COTL1	0.406	10.500	2.683	0.007854909	0.017832443	-3.069	NoSig
CEP41	0.398	7.355	2.681	0.007901931	0.017911847	-3.074	NoSig
CD6	0.434	8.628	2.678	0.007962901	0.018022579	-3.081	NoSig
FKBP15	0.219	9.794	2.677	0.007982867	0.018040309	-3.083	NoSig
BCL2L2	0.210	9.018	2.673	0.008070771	0.018211285	-3.092	NoSig
SEC62	0.301	10.438	2.672	0.008093049	0.018233885	-3.095	NoSig
ANKH	-0.628	5.575	-2.668	0.00819589	0.018437652	-3.106	NoSig
MTMR11	-0.293	8.846	-2.666	0.008253528	0.018539269	-3.112	NoSig
CRYBB2P1	-0.487	6.756	-2.660	0.008377903	0.01879026	-3.125	NoSig
USPL1	0.209	8.529	2.658	0.008422758	0.018826055	-3.130	NoSig
KIAA0430	-0.325	9.448	-2.658	0.008427736	0.018826055	-3.131	NoSig
PLXNA3	-0.280	8.269	-2.658	0.008431844	0.018826055	-3.131	NoSig

MTDH	0.306	11.689	2.652	0.008575489	0.019092365	-3.146	NoSig
PRDM11	-0.343	7.404	-2.652	0.008576978	0.019092365	-3.146	NoSig
NDST2	0.249	8.348	2.652	0.008589638	0.019092365	-3.147	NoSig
EPHA7	-0.613	4.594	-2.650	0.008618284	0.019127446	-3.150	NoSig
RAI1	-0.637	5.332	-2.647	0.00871615	0.019315821	-3.160	NoSig
STAP1	-0.643	6.308	-2.643	0.008813304	0.019502058	-3.170	NoSig
DOK5	-0.302	8.999	-2.630	0.009143121	0.020201814	-3.202	NoSig
FAM53B	0.265	9.720	2.624	0.009299527	0.02051691	-3.217	NoSig
SUV39H2	-0.663	6.318	-2.620	0.009404153	0.020717002	-3.227	NoSig
G6PC	-0.628	3.434	-2.618	0.009459434	0.020807957	-3.232	NoSig
FAR2	-0.665	7.174	-2.617	0.009475558	0.020812636	-3.234	NoSig
NELL1	-0.340	5.010	-2.613	0.009581731	0.0210148	-3.244	NoSig
FRAS1	0.450	7.326	2.612	0.009610204	0.021046206	-3.246	NoSig
PADI2	0.397	10.255	2.609	0.009690939	0.021191803	-3.254	NoSig
KLF6	-0.557	9.723	-2.607	0.009762933	0.021317887	-3.260	NoSig
RPAP3	-0.381	8.232	-2.604	0.009848646	0.021455023	-3.268	NoSig
SLC9A1	0.432	6.240	2.603	0.009854594	0.021455023	-3.268	NoSig
IGFBP5	0.686	10.474	2.601	0.009934374	0.021597097	-3.275	NoSig
HIF3A	-0.382	6.976	-2.591	0.01020214	0.022135224	-3.299	NoSig
NFS1	0.205	8.906	2.591	0.010211677	0.022135224	-3.300	NoSig
UNC5C	-0.481	5.639	-2.589	0.010261287	0.022210384	-3.304	NoSig
PPP2R5C	-0.306	9.831	-2.581	0.010485242	0.022662143	-3.323	NoSig
MANEA	-0.755	5.696	-2.580	0.010518266	0.022700524	-3.326	NoSig
NAV2	0.409	8.600	2.577	0.010621542	0.022890193	-3.334	NoSig
CHML	-0.515	6.741	-2.573	0.010728462	0.023087154	-3.343	NoSig
ICK	0.429	6.172	2.569	0.010843843	0.023301726	-3.352	NoSig
SCIN	-0.454	6.672	-2.566	0.010940947	0.023455334	-3.360	NoSig
PRUNE2	0.352	7.783	2.566	0.010946874	0.023455334	-3.361	NoSig
FBXO7	-0.266	11.382	-2.556	0.011251994	0.024074409	-3.385	NoSig
C12orf49	0.469	9.113	2.554	0.011328191	0.024202614	-3.391	NoSig
NBR1	0.191	9.502	2.535	0.011931753	0.025455547	-3.436	NoSig
SCRN3	-0.302	6.993	-2.531	0.01207712	0.025728763	-3.447	NoSig
JAK1	-0.284	10.644	-2.522	0.01237634	0.026328495	-3.468	NoSig
HDAC4	0.348	7.390	2.516	0.012589551	0.026731625	-3.483	NoSig
CASC5	-0.770	4.939	-2.515	0.012601795	0.026731625	-3.484	NoSig
CSPP1	-0.313	8.872	-2.514	0.012631505	0.026756478	-3.486	NoSig
CSRNP3	-0.472	4.670	-2.514	0.012656966	0.026772273	-3.488	NoSig
RRP8	0.277	8.432	2.513	0.012676177	0.026774823	-3.489	NoSig
KAT6B	0.404	8.770	2.513	0.012698193	0.02678328	-3.490	NoSig
PPP1R12B	-0.222	8.592	-2.510	0.012784014	0.026926103	-3.496	NoSig
TBC1D12	-0.224	8.356	-2.505	0.012955288	0.02724825	-3.508	NoSig
GIN1	-0.238	7.714	-2.504	0.012993897	0.027290855	-3.511	NoSig
FKBP4	0.405	10.697	2.499	0.013194285	0.027672641	-3.524	NoSig
ITSN1	0.366	8.838	2.495	0.013324841	0.027907096	-3.532	NoSig
SSTR3	-0.316	4.872	-2.493	0.013385059	0.027993788	-3.536	NoSig

SCTR	-0.483	5.773	-2.491	0.013490602	0.028174895	-3.543	NoSig
ZBTB3	-0.493	6.310	-2.486	0.013666368	0.028501949	-3.554	NoSig
BAHCC1	0.491	6.838	2.480	0.01386994	0.028885995	-3.567	NoSig
ACAP2	-0.367	9.093	-2.477	0.013994215	0.029104052	-3.575	NoSig
AREG	0.556	6.434	2.476	0.014022016	0.02911506	-3.577	NoSig
MBNL1	0.265	10.930	2.476	0.014038667	0.02911506	-3.578	NoSig
PTPN21	-0.482	6.435	-2.470	0.014260869	0.029533576	-3.592	NoSig
ASXL2	0.159	9.737	2.470	0.014280189	0.029533576	-3.593	NoSig
PACS2	-0.191	8.841	-2.468	0.014344864	0.02962613	-3.597	NoSig
LRRC15	0.350	8.590	2.467	0.014393076	0.029684471	-3.600	NoSig
NFYC	0.307	9.110	2.462	0.014587417	0.030043615	-3.611	NoSig
EIF4G3	-0.296	8.789	-2.460	0.014655035	0.03014113	-3.615	NoSig
PEX2	0.252	9.250	2.459	0.014700252	0.030192368	-3.618	NoSig
UBA6	-0.286	8.062	-2.458	0.014750908	0.03025462	-3.621	NoSig
CALML4	-0.445	7.579	-2.448	0.015129801	0.030989	-3.643	NoSig
MAN1A2	-0.194	10.158	-2.444	0.015295872	0.031286054	-3.652	NoSig
VRK3	0.311	8.714	2.442	0.015398584	0.031426768	-3.658	NoSig
HCG18	-0.412	7.102	-2.442	0.015406936	0.031426768	-3.659	NoSig
IFIH1	0.503	8.709	2.440	0.015456227	0.031484123	-3.662	NoSig
CAD	0.257	9.258	2.434	0.015740835	0.032020001	-3.677	NoSig
RMND1	-0.272	7.590	-2.433	0.015773468	0.03204255	-3.679	NoSig
LSM14B	-0.557	6.679	-2.429	0.015942399	0.032331788	-3.688	NoSig
CHRM3	0.528	6.649	2.429	0.015959336	0.032331788	-3.689	NoSig
HEATR2	0.221	10.054	2.428	0.015993043	0.032355994	-3.691	NoSig
LOC389906	-0.727	5.978	-2.420	0.016322026	0.032976701	-3.709	NoSig
PTK2	0.346	10.081	2.417	0.0164796	0.033249885	-3.717	NoSig
SH3YL1	0.427	10.795	2.403	0.017081534	0.034417671	-3.748	NoSig
TMEM134	0.195	9.496	2.399	0.017255441	0.03472103	-3.757	NoSig
ACVR2B	0.292	8.719	2.398	0.017327244	0.034818395	-3.761	NoSig
LRRC48	-0.249	7.395	-2.395	0.017450648	0.035010376	-3.767	NoSig
BTRC	0.287	7.879	2.395	0.017469872	0.035010376	-3.768	NoSig
GNB5	0.279	7.649	2.394	0.017514815	0.035053203	-3.770	NoSig
VNN3	-0.447	5.716	-2.388	0.017762286	0.035500697	-3.782	NoSig
LRRC41	-0.305	9.072	-2.384	0.017951692	0.035831096	-3.791	NoSig
CD209	-0.252	7.874	-2.383	0.01800961	0.035898512	-3.794	NoSig
SDS	0.303	8.329	2.382	0.018043208	0.035917336	-3.796	NoSig
VCPIP1	-0.510	6.326	-2.381	0.018103749	0.035989671	-3.798	NoSig
ZNF230	-0.443	6.000	-2.376	0.01836233	0.036454986	-3.811	NoSig
SOBP	0.398	7.800	2.375	0.018390257	0.03646175	-3.812	NoSig
MEIS1	-0.289	7.573	-2.367	0.01878717	0.037199096	-3.830	NoSig
SAMHD1	-0.434	8.762	-2.366	0.018849801	0.037273477	-3.833	NoSig
FAM178A	-0.217	7.894	-2.365	0.018903171	0.037329371	-3.836	NoSig
PEX13	-0.482	7.775	-2.362	0.019022559	0.037515312	-3.841	NoSig
CD55	-0.309	9.828	-2.357	0.019316201	0.038043962	-3.854	NoSig
SYNRG	0.316	8.411	2.356	0.019371094	0.03810161	-3.857	NoSig

SLC30A5	-0.318	8.468	-2.352	0.019525709	0.038354992	-3.864	NoSig
ERCC8	-0.232	8.116	-2.351	0.019585594	0.038421871	-3.866	NoSig
CSF3R	-0.500	7.553	-2.346	0.019854922	0.038898906	-3.878	NoSig
CARHSP1	0.438	8.453	2.345	0.019926202	0.038987187	-3.881	NoSig
CLDND1	-0.380	9.239	-2.330	0.020726625	0.040499988	-3.915	NoSig
DHX30	-0.143	9.453	-2.327	0.020885641	0.04075715	-3.922	NoSig
RBPMS	0.416	8.908	2.326	0.020941143	0.040811901	-3.924	NoSig
ACTR3B	-0.360	8.749	-2.324	0.021049045	0.040930433	-3.928	NoSig
METTL8	-0.719	6.574	-2.324	0.021057015	0.040930433	-3.929	NoSig
NDUFA10	0.244	10.359	2.320	0.021236254	0.041224947	-3.936	NoSig
FOXC1	0.420	9.884	2.316	0.021496711	0.041676153	-3.946	NoSig
DLEU2	-0.272	7.546	-2.314	0.02156185	0.041748008	-3.949	NoSig
BIN3	0.228	8.769	2.314	0.021590273	0.041748681	-3.950	NoSig
TRAPPC10	-0.477	7.120	-2.309	0.021886143	0.042265837	-3.962	NoSig
NEUROD2	0.478	7.596	2.307	0.021987788	0.042407057	-3.966	NoSig
CHPT1	0.361	10.308	2.306	0.022044622	0.042461597	-3.968	NoSig
KIDINS220	0.217	9.493	2.304	0.022141087	0.042577917	-3.972	NoSig
HIPK1	0.205	9.111	2.304	0.022162278	0.042577917	-3.973	NoSig
CLN6	-0.784	6.326	-2.303	0.022209266	0.042613134	-3.974	NoSig
GFOD1	0.222	9.350	2.300	0.022377201	0.042880022	-3.981	NoSig
MARK1	-0.245	8.034	-2.299	0.022459467	0.042982274	-3.984	NoSig
CYP4B1	-0.607	5.299	-2.297	0.022560765	0.04312064	-3.988	NoSig
HDAC9	-0.421	7.484	-2.295	0.022644256	0.043224658	-3.991	NoSig
DENND4A	-0.614	5.794	-2.292	0.022850222	0.043561898	-3.999	NoSig
CTNNB1	-0.309	11.149	-2.291	0.02293189	0.043661614	-4.002	NoSig
TAF5	-0.321	8.315	-2.289	0.023003146	0.043741275	-4.004	NoSig
PIGL	0.364	8.282	2.288	0.0230885	0.043813594	-4.008	NoSig
NEMF	-0.293	9.410	-2.288	0.023100106	0.043813594	-4.008	NoSig
MCCC2	0.312	9.851	2.287	0.023138332	0.043830191	-4.009	NoSig
MBNL2	0.442	9.205	2.285	0.023276859	0.044036501	-4.015	NoSig
LMO7	-0.493	5.799	-2.282	0.023448193	0.044304273	-4.021	NoSig
PPFIA2	-0.353	5.786	-2.279	0.023643816	0.044617201	-4.028	NoSig
LINS	-0.591	5.614	-2.277	0.023731899	0.044726659	-4.031	NoSig
ZNF493	-0.573	5.939	-2.275	0.023890666	0.044968887	-4.037	NoSig
DOPEY1	0.182	8.341	2.271	0.024093119	0.045292071	-4.044	NoSig
PRR14	-0.166	10.162	-2.271	0.024123287	0.045292071	-4.045	NoSig
ATF1	-0.443	8.049	-2.270	0.024153741	0.045292071	-4.046	NoSig
RARS2	-0.711	4.803	-2.264	0.024559325	0.045994605	-4.060	NoSig
MAPK1	-0.194	9.903	-2.263	0.024590976	0.04599595	-4.062	NoSig
TBC1D1	0.204	9.387	2.260	0.024797277	0.046323556	-4.069	NoSig
FGD6	-0.442	6.992	-2.259	0.024837754	0.046340954	-4.070	NoSig
IREB2	-0.323	7.079	-2.257	0.024970805	0.046530811	-4.075	NoSig
KIAA1467	0.381	8.402	2.255	0.025134579	0.04677737	-4.080	NoSig
PTRF	0.518	9.569	2.251	0.02534051	0.047101673	-4.087	NoSig
SAR1B	-0.318	8.926	-2.248	0.02556002	0.047450375	-4.095	NoSig

FOXJ2	0.286	9.237	2.243	0.025855897	0.0479398	-4.104	NoSig
DISC1	0.297	6.983	2.240	0.026106025	0.048343286	-4.113	NoSig
PTPRS	-0.465	5.312	-2.238	0.026213992	0.048482843	-4.116	NoSig
GPR6	-0.444	4.966	-2.237	0.026274942	0.048535204	-4.118	NoSig
APBB1IP	-0.372	7.265	-2.235	0.026388547	0.048684577	-4.122	NoSig
NRP1	0.463	7.812	2.231	0.026707981	0.049212847	-4.132	NoSig
MTUS2	-0.418	4.039	-2.227	0.026955855	0.049608115	-4.140	NoSig
MCF2L	0.259	9.374	2.226	0.026998118	0.049624477	-4.141	NoSig
AGBL3	-0.676	3.486	-2.224	0.027168984	0.049876888	-4.147	NoSig
DTYMK	-0.332	8.817	-2.220	0.027417185	0.050270473	-4.154	NoSig
FRMD4A	0.318	7.912	2.217	0.027666937	0.05066593	-4.162	NoSig
TMLHE	-0.438	5.517	-2.214	0.027833668	0.050908566	-4.167	NoSig
TTF2	0.309	6.987	2.210	0.02816313	0.051447879	-4.177	NoSig
TRIM37	0.234	9.596	2.203	0.028634903	0.052245522	-4.191	NoSig
SLC6A11	-0.417	5.159	-2.196	0.029130979	0.053085497	-4.206	NoSig
ATP6V1H	0.230	9.835	2.189	0.029656303	0.053976649	-4.221	NoSig
SYN2	-0.365	8.053	-2.187	0.029783472	0.054141837	-4.225	NoSig
PCSK6	0.314	7.651	2.181	0.030228735	0.054884162	-4.237	NoSig
PDE1A	-0.375	6.238	-2.180	0.030335398	0.055010655	-4.240	NoSig
EBF2	-0.418	5.783	-2.175	0.030676016	0.055553581	-4.250	NoSig
PRF1	-0.344	8.739	-2.175	0.030709512	0.055553581	-4.251	NoSig
TXLNA	0.344	8.507	2.169	0.031150748	0.056283307	-4.263	NoSig
ZCCHC10	0.276	7.427	2.168	0.031251016	0.056395948	-4.265	NoSig
KDM4C	0.152	8.960	2.167	0.031320447	0.056452732	-4.267	NoSig
SLC44A4	-0.467	5.964	-2.163	0.031584853	0.056860384	-4.274	NoSig
PTPN7	0.237	8.750	2.154	0.032299218	0.058076103	-4.293	NoSig
PWP2	0.398	7.919	2.154	0.032358753	0.058112882	-4.295	NoSig
RUFY2	-0.409	6.598	-2.150	0.032661354	0.058585566	-4.303	NoSig
LTB	0.448	8.945	2.146	0.032926096	0.058989283	-4.310	NoSig
RPS6KA5	0.325	7.960	2.145	0.033011844	0.059071736	-4.312	NoSig
HP1BP3	-0.494	6.273	-2.141	0.033334803	0.059577947	-4.320	NoSig
RHOBTB2	-0.342	5.955	-2.138	0.033598185	0.059976591	-4.327	NoSig
CNTN1	0.378	7.720	2.132	0.034091497	0.060784241	-4.339	NoSig
BSDC1	0.181	9.389	2.132	0.034140095	0.060797989	-4.340	NoSig
UHRF1BP1L	-0.481	6.920	-2.127	0.034550985	0.061456119	-4.350	NoSig
KCTD17	-0.352	6.066	-2.122	0.034911472	0.061999932	-4.359	NoSig
RBFOX1	0.425	6.440	2.122	0.03494011	0.061999932	-4.360	NoSig
KDSR	0.171	9.494	2.120	0.03508437	0.062116674	-4.363	NoSig
OTOF	0.410	4.029	2.120	0.035119028	0.062116674	-4.364	NoSig
ZNF135	-0.594	5.936	-2.120	0.035131219	0.062116674	-4.364	NoSig
HOMER2	-0.295	8.182	-2.118	0.035303049	0.062346358	-4.368	NoSig
RPRD1A	-0.280	8.787	-2.115	0.035506599	0.06263145	-4.373	NoSig
ITGAL	-0.353	7.479	-2.111	0.035858483	0.063177208	-4.381	NoSig
RARA	0.342	7.549	2.103	0.036572073	0.064358191	-4.398	NoSig
CRTAC1	0.450	7.285	2.102	0.036657376	0.064403798	-4.400	NoSig

GRIK2	-0.261	4.010	-2.102	0.036684611	0.064403798	-4.401	NoSig
FBXO22	-0.446	5.191	-2.099	0.036922827	0.064745571	-4.406	NoSig
HIST1H1T	-0.387	5.127	-2.096	0.037179972	0.065119691	-4.412	NoSig
VPS13B	-0.276	8.755	-2.084	0.038322083	0.067041102	-4.437	NoSig
ATXN1	0.294	8.442	2.083	0.038438502	0.067165749	-4.440	NoSig
GINS4	-0.319	7.457	-2.073	0.039283623	0.06856191	-4.458	NoSig
CBS	-0.364	8.587	-2.068	0.0397779	0.069343186	-4.468	NoSig
SIRT2	0.252	9.816	2.066	0.040032096	0.069704598	-4.474	NoSig
ZNF506	-0.362	6.967	-2.062	0.040415601	0.070268184	-4.482	NoSig
ZNF451	-0.260	8.797	-2.061	0.04045028	0.070268184	-4.483	NoSig
RP2	0.279	6.416	2.060	0.040593315	0.070434375	-4.485	NoSig
ABCC9	-0.255	7.411	-2.038	0.042729225	0.074054029	-4.528	NoSig
ARHGAP22	0.319	7.192	2.035	0.043009504	0.074453007	-4.534	NoSig
ARPP21	-0.344	4.215	-2.025	0.044043337	0.076154002	-4.554	NoSig
C11orf57	-0.161	8.951	-2.021	0.044498847	0.076852248	-4.562	NoSig
TEAD1	-0.309	7.898	-2.016	0.04501786	0.077658419	-4.572	NoSig
TONSL	-0.479	3.563	-2.011	0.045547064	0.078480283	-4.581	NoSig
FGFR4	-0.405	6.200	-2.010	0.045670128	0.07851886	-4.584	NoSig
ADAMTSL3	0.248	6.979	2.010	0.04567506	0.07851886	-4.584	NoSig
KCNMB1	0.280	8.752	2.009	0.045745226	0.078548673	-4.585	NoSig
TRIM36	0.387	5.600	1.994	0.047329352	0.081175025	-4.613	NoSig
C10orf76	-0.316	7.346	-1.992	0.0475974	0.081540708	-4.618	NoSig
RHOB	0.346	9.587	1.989	0.047923422	0.08200475	-4.624	NoSig
TYMS	0.293	11.835	1.984	0.04843888	0.08279151	-4.633	NoSig
ZBED1	0.204	9.030	1.976	0.049339862	0.084234643	-4.648	NoSig
TROAP	0.176	9.177	1.975	0.049490415	0.084394779	-4.650	NoSig
SLC5A3	-0.272	9.625	-1.970	0.050136707	0.085339779	-4.661	NoSig
ASPHD1	-0.443	4.613	-1.969	0.050159359	0.085339779	-4.661	NoSig
ZNF26	-0.180	8.625	-1.966	0.050594454	0.08598166	-4.669	NoSig
SLC29A2	-0.325	7.003	-1.965	0.050709391	0.086078612	-4.670	NoSig
AHNAK2	-0.481	8.413	-1.963	0.050918515	0.086335041	-4.674	NoSig
TNR	-0.218	7.305	-1.960	0.051195754	0.086706248	-4.678	NoSig
DIP2C	-0.239	9.448	-1.954	0.051925566	0.087842226	-4.690	NoSig
ATP4B	-0.309	6.160	-1.953	0.052061016	0.087971285	-4.692	NoSig
PPARA	0.229	6.974	1.951	0.052346008	0.088352457	-4.697	NoSig
DOCK5	0.303	7.351	1.950	0.052438696	0.08840855	-4.698	NoSig
KANSL3	-0.438	5.562	-1.945	0.053055333	0.089346864	-4.708	NoSig
EMID1	0.435	7.362	1.944	0.053118396	0.089351872	-4.709	NoSig
PTPRB	0.360	5.826	1.943	0.053337387	0.089618863	-4.712	NoSig
C1orf61	0.433	8.034	1.939	0.053763562	0.090232975	-4.719	NoSig
FBN2	0.341	7.201	1.934	0.054377499	0.091106102	-4.728	NoSig
ARHGAP29	0.299	9.750	1.934	0.054406334	0.091106102	-4.728	NoSig
MS4A4A	-0.422	8.141	-1.933	0.054571093	0.09122331	-4.731	NoSig
RFC2	-0.197	8.975	-1.932	0.054599022	0.09122331	-4.731	NoSig
EGFR	0.289	8.493	1.926	0.055384377	0.09236847	-4.743	NoSig

H6PD	-0.279	7.497	-1.926	0.055408659	0.09236847	-4.743	NoSig
RANBP10	-0.155	8.459	-1.923	0.055769297	0.09286556	-4.749	NoSig
CLK4	-0.190	9.234	-1.920	0.056089917	0.093294974	-4.754	NoSig
FCER1G	-0.259	10.648	-1.917	0.056559679	0.09397122	-4.760	NoSig
ZNF207	-0.154	11.415	-1.915	0.056798334	0.094246832	-4.764	NoSig
SEPW1	-0.210	11.545	-1.914	0.056852326	0.094246832	-4.765	NoSig
ZNF654	-0.268	7.889	-1.908	0.057699224	0.095350685	-4.777	NoSig
OR2H1	0.313	6.587	1.908	0.0577015	0.095350685	-4.777	NoSig
TAPBP	0.237	11.091	1.908	0.057710569	0.095350685	-4.777	NoSig
MPP6	-0.469	6.017	-1.907	0.057796304	0.095386353	-4.778	NoSig
OGDH	-0.169	8.630	-1.899	0.058831237	0.096986751	-4.793	NoSig
BTN2A2	0.158	9.126	1.897	0.059108266	0.097335539	-4.797	NoSig
FAM188A	-0.310	8.024	-1.894	0.059533122	0.097926717	-4.802	NoSig
SLC33A1	-0.201	9.108	-1.889	0.060264658	0.099020494	-4.812	NoSig
DNAH3	0.502	10.157	1.882	0.061107422	0.100294411	-4.824	NoSig
MBP	0.245	9.241	1.879	0.061499467	0.10082658	-4.829	NoSig
FCER1A	0.353	6.200	1.877	0.061864817	0.101313858	-4.834	NoSig
SIRPB1	-0.257	6.012	-1.875	0.062164361	0.101692414	-4.838	NoSig
KCNK1	0.303	9.414	1.873	0.062327402	0.101847084	-4.840	NoSig
ST6GALNAC5	0.309	7.777	1.870	0.062837492	0.102567893	-4.847	NoSig
SMAD4	-0.162	8.607	-1.868	0.063024617	0.102760533	-4.849	NoSig
SPAG16	0.303	7.196	1.864	0.063683667	0.103721372	-4.857	NoSig
SMC1A	-0.232	10.464	-1.863	0.063848779	0.103876514	-4.860	NoSig
ARHGDIB	-0.305	11.803	-1.856	0.064751867	0.10523063	-4.871	NoSig
C6orf123	-0.282	6.798	-1.851	0.065475371	0.106290259	-4.880	NoSig
FGF7	-0.274	6.921	-1.850	0.065631794	0.106363198	-4.882	NoSig
NAALAD2	-0.423	3.919	-1.850	0.065663359	0.106363198	-4.882	NoSig
STIM1	-0.134	8.280	-1.849	0.065772756	0.10642447	-4.884	NoSig
NR1H4	0.375	5.616	1.845	0.066324327	0.107097338	-4.890	NoSig
LRRK1	-0.246	7.372	-1.845	0.066332648	0.107097338	-4.891	NoSig
ZAP70	-0.367	6.661	-1.842	0.06685918	0.107830369	-4.897	NoSig
VPS8	0.147	8.594	1.840	0.067176435	0.108224657	-4.901	NoSig
MAOB	0.555	7.612	1.833	0.068204579	0.10976213	-4.913	NoSig
ERAP2	-0.530	7.049	-1.832	0.068359846	0.109893072	-4.915	NoSig
ETV4	-0.325	5.746	-1.830	0.068542702	0.109977656	-4.917	NoSig
MPHOSPH9	-0.548	5.364	-1.830	0.068560382	0.109977656	-4.917	NoSig
GNG12	-0.244	9.599	-1.828	0.068885588	0.110380248	-4.921	NoSig
ATF3	0.214	9.283	1.825	0.069307719	0.110827574	-4.926	NoSig
ABI3BP	-0.374	5.652	-1.825	0.069313816	0.110827574	-4.926	NoSig
CDHR1	0.272	3.389	1.824	0.06951332	0.111027183	-4.929	NoSig
SREK1	0.133	9.347	1.817	0.070614943	0.112665687	-4.941	NoSig
CXADR	0.398	10.313	1.809	0.071825035	0.114382542	-4.955	NoSig
GPRIN2	0.221	8.924	1.809	0.071844851	0.114382542	-4.955	NoSig
TATDN2	0.161	9.837	1.806	0.072319483	0.114935274	-4.961	NoSig
MUC4	0.353	5.493	1.806	0.072346615	0.114935274	-4.961	NoSig

C5orf28	-0.248	7.673	-1.800	0.073176596	0.116129773	-4.970	NoSig
PRR16	-0.546	4.462	-1.795	0.074022128	0.11734638	-4.979	NoSig
PAK1	0.361	6.208	1.788	0.075085292	0.118854831	-4.991	NoSig
RBM15	-0.291	9.122	-1.788	0.075133518	0.118854831	-4.991	NoSig
PPP1R11	-0.184	11.130	-1.786	0.075421966	0.119180894	-4.994	NoSig
CHRNA10	-0.166	7.159	-1.786	0.075499934	0.119180894	-4.995	NoSig
COBL	0.447	8.214	1.785	0.075679281	0.119337317	-4.997	NoSig
ITGB8	-0.264	7.222	-1.783	0.07600992	0.119731728	-5.001	NoSig
ATP6V1B1	-0.382	6.758	-1.779	0.076531507	0.120368794	-5.006	NoSig
B4GALNT1	0.284	7.481	1.779	0.076651978	0.120368794	-5.007	NoSig
TBC1D8B	-0.198	8.083	-1.779	0.076657194	0.120368794	-5.008	NoSig
CBR4	-0.209	8.246	-1.778	0.076760024	0.120403117	-5.009	NoSig
PTPRC	0.339	10.049	1.777	0.076884239	0.120470878	-5.010	NoSig
NT5C	0.265	7.395	1.776	0.077131759	0.120687994	-5.013	NoSig
ZNF638	0.185	10.599	1.776	0.077185126	0.120687994	-5.013	NoSig
PAK2	-0.164	9.338	-1.771	0.077888596	0.121660024	-5.020	NoSig
KCNK10	0.281	6.315	1.757	0.080378044	0.125416738	-5.046	NoSig
MPDU1	0.319	7.569	1.756	0.080514292	0.125497644	-5.047	NoSig
PAQR3	-0.292	7.275	-1.737	0.083763902	0.130317968	-5.079	NoSig
PBLD	-0.189	7.176	-1.737	0.083782096	0.130317968	-5.079	NoSig
USP49	-0.355	4.324	-1.724	0.086163369	0.133786858	-5.101	NoSig
MKS1	-0.154	7.965	-1.724	0.086192206	0.133786858	-5.101	NoSig
RNF170	0.263	7.026	1.721	0.086608341	0.1342926	-5.105	NoSig
DTWD1	-0.377	6.694	-1.720	0.086739641	0.13435609	-5.106	NoSig
FCN2	-0.276	7.756	-1.710	0.08874508	0.13731939	-5.125	NoSig
SPAG9	0.164	9.241	1.709	0.08884072	0.13732448	-5.125	NoSig
ARHGEF10	0.357	8.018	1.705	0.089657337	0.138442846	-5.133	NoSig
RANGAP1	-0.158	8.821	-1.701	0.090264697	0.139236104	-5.138	NoSig
MAD2L1	0.244	9.093	1.699	0.090739164	0.13982294	-5.142	NoSig
OTUB2	0.266	5.232	1.695	0.091433141	0.140746461	-5.148	NoSig
MAPKBP1	-0.345	6.331	-1.694	0.091730165	0.141057658	-5.151	NoSig
SLC2A11	-0.332	6.013	-1.684	0.093625234	0.143823061	-5.167	NoSig
ABI1	-0.172	9.931	-1.680	0.094300613	0.144711055	-5.173	NoSig
SEC23IP	0.139	8.671	1.675	0.095330802	0.146141136	-5.181	NoSig
GABRB2	0.343	6.939	1.674	0.095462335	0.146192061	-5.182	NoSig
ZDHHC18	0.229	8.802	1.674	0.095607184	0.146263254	-5.184	NoSig
CDH7	0.235	3.671	1.668	0.096642298	0.147694859	-5.192	NoSig
KRT7	0.370	10.825	1.663	0.097778418	0.149277729	-5.201	NoSig
PHTF2	0.167	8.759	1.659	0.098607044	0.150388384	-5.208	NoSig
RAC1	0.151	12.639	1.658	0.098818911	0.150557091	-5.210	NoSig
CCDC69	-0.429	6.459	-1.656	0.099068133	0.1506356	-5.212	NoSig
SYCE1L	-0.495	6.098	-1.656	0.099073044	0.1506356	-5.212	NoSig
TFAP2B	-0.620	6.221	-1.651	0.100138179	0.152099562	-5.220	NoSig
DDAH1	0.252	10.190	1.647	0.101013853	0.15327306	-5.227	NoSig
ELAVL3	-0.208	4.808	-1.646	0.101140447	0.153308711	-5.228	NoSig

GALE	0.171	7.990	1.640	0.102366821	0.155009637	-5.237	NoSig
SP140L	-0.245	8.115	-1.639	0.102719425	0.155357507	-5.240	NoSig
SVEP1	-0.251	6.115	-1.638	0.102805506	0.155357507	-5.241	NoSig
NUMA1	0.187	9.475	1.629	0.104639567	0.157968565	-5.255	NoSig
MCTP2	0.191	7.744	1.627	0.105158267	0.158487779	-5.259	NoSig
PPA2	-0.143	10.254	-1.627	0.105196663	0.158487779	-5.259	NoSig
GAD2	0.327	6.076	1.625	0.105602327	0.158937915	-5.262	NoSig
MCM9	-0.159	8.358	-1.622	0.106230345	0.159721459	-5.266	NoSig
FCGR2A	-0.249	9.174	-1.619	0.106877208	0.160531726	-5.271	NoSig
DOCK2	0.340	7.277	1.618	0.107037525	0.160610293	-5.272	NoSig
ZNF215	0.207	4.860	1.617	0.10739477	0.160983894	-5.275	NoSig
DDX31	-0.253	6.128	-1.610	0.108806726	0.162867417	-5.285	NoSig
GRK4	0.240	6.461	1.610	0.108870351	0.162867417	-5.286	NoSig
FXYD2	-0.261	6.557	-1.608	0.109202997	0.163200861	-5.288	NoSig
EPB41L4A	-0.259	5.585	-1.607	0.109509528	0.163494647	-5.290	NoSig
TRPV5	0.263	7.042	1.601	0.110773367	0.164965412	-5.299	NoSig
CD44	-0.227	11.948	-1.601	0.110791373	0.164965412	-5.299	NoSig
PHF20L1	0.151	8.445	1.601	0.110827469	0.164965412	-5.300	NoSig
GPR12	0.245	8.307	1.598	0.111467192	0.165751714	-5.304	NoSig
CDH4	0.295	7.494	1.597	0.111704514	0.165938673	-5.306	NoSig
HLA.DOB	0.379	6.962	1.595	0.112197382	0.166504498	-5.309	NoSig
NOL3	0.228	8.350	1.592	0.112719929	0.167113195	-5.313	NoSig
UEVLD	-0.199	7.518	-1.591	0.113092172	0.167498068	-5.315	NoSig
C18orf25	-0.170	7.893	-1.589	0.113569797	0.168038097	-5.319	NoSig
GRIK5	0.342	8.226	1.584	0.1145685	0.169347275	-5.325	NoSig
RGS7	-0.274	5.962	-1.582	0.114990523	0.169802292	-5.328	NoSig
KCNE4	-0.376	5.649	-1.577	0.116187412	0.171399485	-5.336	NoSig
CNTLN	-0.297	6.353	-1.565	0.11893613	0.175280501	-5.354	NoSig
SOS1	0.195	8.568	1.564	0.119133545	0.175397605	-5.356	NoSig
PGM5	-0.260	4.632	-1.564	0.119348958	0.175540949	-5.357	NoSig
KIFC3	0.189	7.749	1.562	0.119663487	0.175829649	-5.359	NoSig
PDZK1IP1	-0.291	9.814	-1.562	0.119825107	0.17589332	-5.360	NoSig
WBSCR16	0.127	8.978	1.560	0.120087358	0.17610444	-5.362	NoSig
GABRB1	0.279	6.441	1.560	0.120250159	0.176169445	-5.363	NoSig
HEMK1	0.130	8.106	1.556	0.12104609	0.17716096	-5.368	NoSig
CLEC2B	0.272	9.262	1.554	0.121546139	0.177717905	-5.371	NoSig
TLE6	-0.187	6.729	-1.553	0.121835178	0.17796553	-5.373	NoSig
GPR126	-0.226	8.151	-1.548	0.123031964	0.179537322	-5.380	NoSig
STX6	-0.113	9.943	-1.547	0.123367238	0.179741069	-5.383	NoSig
HMGA2	-0.277	7.158	-1.546	0.123413337	0.179741069	-5.383	NoSig
LYST	0.181	9.273	1.545	0.123764219	0.180075727	-5.385	NoSig
CP	-0.371	8.926	-1.541	0.124721602	0.181291321	-5.391	NoSig
DET1	-0.113	8.252	-1.538	0.125492787	0.182234154	-5.396	NoSig
SPOCK3	-0.343	4.891	-1.536	0.125866037	0.182597851	-5.398	NoSig
VNN1	-0.382	5.882	-1.531	0.127267192	0.184450599	-5.407	NoSig

SNAP25	0.376	7.813	1.529	0.127766714	0.184994259	-5.410	NoSig
CASS4	-0.287	4.556	-1.521	0.129714583	0.187631893	-5.421	NoSig
C16orf45	-0.234	7.277	-1.519	0.130208484	0.18816328	-5.424	NoSig
SLC17A1	0.340	7.000	1.511	0.132273595	0.190958485	-5.436	NoSig
SYNJ2	0.151	8.770	1.510	0.132399595	0.190958485	-5.437	NoSig
RRP12	-0.207	7.271	-1.509	0.132660086	0.191148786	-5.438	NoSig
RUNX1T1	-0.150	8.685	-1.502	0.13455334	0.193563095	-5.449	NoSig
CA6	0.340	6.813	1.502	0.134595992	0.193563095	-5.449	NoSig
HBB	0.844	13.381	1.499	0.135236106	0.194295739	-5.453	NoSig
CCL5	-0.257	10.034	-1.498	0.13556008	0.194573204	-5.455	NoSig
AMMECR1	0.165	8.635	1.493	0.136839588	0.196220316	-5.462	NoSig
BRWD1	-0.303	7.141	-1.485	0.1389359	0.198867426	-5.474	NoSig
HEATR1	0.122	10.298	1.485	0.138953097	0.198867426	-5.474	NoSig
RECK	0.356	11.320	1.484	0.139222015	0.199060708	-5.475	NoSig
SPP1	-0.315	12.502	-1.484	0.139357632	0.199063207	-5.476	NoSig
TRPM3	0.271	7.827	1.479	0.140536263	0.200554149	-5.482	NoSig
DIXDC1	-0.296	6.543	-1.478	0.140915259	0.200902196	-5.484	NoSig
SCAMP1	0.135	10.016	1.476	0.141379682	0.201371253	-5.487	NoSig
SRCAP	0.178	7.925	1.466	0.144165233	0.205142298	-5.502	NoSig
LPGAT1	-0.182	9.560	-1.465	0.144466433	0.205374365	-5.503	NoSig
SLC25A28	0.203	8.555	1.463	0.144939288	0.20567218	-5.506	NoSig
DST	-0.230	9.300	-1.463	0.144952552	0.20567218	-5.506	NoSig
DZANK1	-0.281	6.884	-1.461	0.145315304	0.205990331	-5.508	NoSig
PRPSAP1	0.129	10.140	1.460	0.145811323	0.206496607	-5.510	NoSig
CUX2	-0.274	5.424	-1.458	0.146142506	0.206768703	-5.512	NoSig
PEX14	-0.238	8.174	-1.456	0.14683508	0.207551107	-5.515	NoSig
CA8	0.310	6.706	1.453	0.147562276	0.208380915	-5.519	NoSig
KIF5C	-0.182	8.274	-1.450	0.148490047	0.209492126	-5.524	NoSig
ARHGEF7	0.113	9.818	1.447	0.149393516	0.210566975	-5.528	NoSig
TMEM80	-0.177	7.546	-1.442	0.150738644	0.212261708	-5.535	NoSig
ATP9B	0.183	7.546	1.440	0.151396515	0.212986393	-5.538	NoSig
SV2C	0.272	6.278	1.437	0.152118004	0.213799123	-5.542	NoSig
TRMT2B	0.202	8.834	1.430	0.153992347	0.216229103	-5.551	NoSig
CDK12	-0.156	8.033	-1.429	0.154495097	0.216664789	-5.554	NoSig
WWC1	0.138	9.228	1.428	0.154594043	0.216664789	-5.554	NoSig
ZFYVE16	-0.283	7.803	-1.420	0.156890279	0.219675936	-5.565	NoSig
CARD14	0.174	7.811	1.415	0.158527712	0.221759838	-5.573	NoSig
TTC23	-0.125	8.261	-1.413	0.159075553	0.222317056	-5.575	NoSig
WNT4	-0.316	5.260	-1.408	0.160456712	0.224036742	-5.582	NoSig
CACNG5	-0.256	5.517	-1.397	0.163774089	0.2284541	-5.597	NoSig
GPR97	0.297	7.486	1.393	0.164906606	0.229818297	-5.602	NoSig
RUVBL2	-0.259	9.996	-1.389	0.166169733	0.231361791	-5.608	NoSig
SAMSN1	-0.235	8.064	-1.388	0.166618271	0.231769288	-5.610	NoSig
DPP6	0.248	6.916	1.375	0.170438178	0.236861281	-5.627	NoSig
ABCA8	-0.292	5.675	-1.371	0.171656614	0.238331825	-5.632	NoSig

IQSEC3	-0.216	5.541	-1.364	0.173851395	0.241153941	-5.641	NoSig
KCNK15	0.314	4.270	1.352	0.177640668	0.246180497	-5.657	NoSig
ITGB2	-0.193	10.455	-1.349	0.178573932	0.247243423	-5.661	NoSig
ESRRA	0.099	9.105	1.349	0.17877919	0.247297354	-5.662	NoSig
CCDC82	-0.204	6.679	-1.348	0.179175183	0.247614774	-5.664	NoSig
ADAM22	0.262	7.566	1.342	0.181084255	0.250020694	-5.671	NoSig
DHX35	0.244	6.646	1.340	0.181495918	0.250244811	-5.673	NoSig
FCHSD2	0.254	8.012	1.340	0.181583154	0.250244811	-5.673	NoSig
HDGFRP3	0.166	8.339	1.339	0.18189057	0.250414576	-5.675	NoSig
KCNQ3	-0.161	7.317	-1.339	0.182104769	0.250414576	-5.675	NoSig
PAX7	-0.200	4.530	-1.338	0.182211548	0.250414576	-5.676	NoSig
AKT2	0.233	7.126	1.337	0.182726967	0.250891044	-5.678	NoSig
IL16	0.174	8.871	1.335	0.183323645	0.2514781	-5.680	NoSig
FYCO1	-0.131	7.937	-1.334	0.183576494	0.251592855	-5.681	NoSig
TNK2	0.199	8.174	1.331	0.18469743	0.252896021	-5.686	NoSig
TMCC2	0.228	7.234	1.329	0.185280734	0.253461317	-5.688	NoSig
CDCP1	0.184	8.837	1.326	0.186041818	0.254268551	-5.691	NoSig
PDE6B	-0.243	4.798	-1.324	0.186826325	0.255106286	-5.694	NoSig
STAT5B	0.137	9.003	1.319	0.188415049	0.257039613	-5.700	NoSig
ABCC4	-0.248	7.052	-1.317	0.189133846	0.257783711	-5.703	NoSig
BBS9	0.139	7.062	1.315	0.189746048	0.258381295	-5.705	NoSig
TRMT61A	-0.155	8.272	-1.312	0.190896789	0.259710454	-5.710	NoSig
AKR1C1	0.334	9.630	1.311	0.191092649	0.259739277	-5.711	NoSig
NRXN1	0.300	5.971	1.309	0.191770884	0.260423109	-5.713	NoSig
ZNF12	-0.203	8.696	-1.305	0.193144603	0.262049293	-5.718	NoSig
ATP10A	0.136	7.654	1.299	0.195370219	0.264827271	-5.727	NoSig
EYA4	0.275	5.994	1.297	0.195812939	0.265185647	-5.728	NoSig
LAMA3	-0.308	6.285	-1.296	0.196360995	0.265685896	-5.730	NoSig
CTNNA3	0.234	4.930	1.295	0.196760127	0.265968408	-5.732	NoSig
ASAH1	-0.164	12.058	-1.294	0.196927517	0.265968408	-5.732	NoSig
SNRK	0.126	9.420	1.291	0.198029223	0.267213661	-5.736	NoSig
DENND1B	0.133	8.361	1.289	0.198677737	0.26784569	-5.739	NoSig
DLGAP4	0.098	8.021	1.286	0.199810489	0.268972102	-5.743	NoSig
PELO	-0.146	8.777	-1.286	0.199875032	0.268972102	-5.743	NoSig
NAA15	0.133	8.521	1.284	0.200603059	0.26970773	-5.746	NoSig
HILPDA	-0.135	10.325	-1.267	0.206311618	0.27704523	-5.766	NoSig
GPR125	0.152	8.793	1.267	0.206613818	0.27704523	-5.767	NoSig
CYP11B1	0.211	7.259	1.267	0.206619476	0.27704523	-5.767	NoSig
HRH3	-0.158	5.419	-1.266	0.206890468	0.277158673	-5.768	NoSig
RECQL4	-0.324	6.729	-1.262	0.208201916	0.278664491	-5.772	NoSig
TYR	0.278	6.686	1.261	0.208616939	0.278934159	-5.774	NoSig
MITF	0.124	7.837	1.261	0.20877856	0.278934159	-5.774	NoSig
CD79A	0.204	8.347	1.260	0.209123517	0.279144228	-5.776	NoSig
STAB1	0.135	9.531	1.258	0.209705439	0.279669944	-5.778	NoSig
SLC1A2	0.194	7.331	1.257	0.210181608	0.28005381	-5.779	NoSig

PRKCA	0.177	7.911	1.256	0.210427543	0.280130489	-5.780	NoSig
ATF6B	-0.115	8.682	-1.255	0.21068802	0.280226374	-5.781	NoSig
BEST1	-0.192	6.818	-1.254	0.211036299	0.280438764	-5.782	NoSig
EFNA2	0.201	5.183	1.253	0.211647784	0.281000227	-5.784	NoSig
ZMAT3	-0.130	8.005	-1.250	0.212600353	0.282013136	-5.787	NoSig
SIM1	-0.258	5.979	-1.248	0.213171264	0.282518421	-5.789	NoSig
PLD1	0.113	8.456	1.245	0.2143329	0.283805006	-5.793	NoSig
ZDHHC14	-0.227	6.243	-1.243	0.215180653	0.284674049	-5.796	NoSig
LTBR	0.117	9.218	1.236	0.217942237	0.288071205	-5.805	NoSig
RPP30	0.105	9.336	1.233	0.218802595	0.288951562	-5.808	NoSig
NLGN4X	-0.309	5.254	-1.232	0.21912034	0.289114415	-5.809	NoSig
ENTPD3	0.212	7.310	1.224	0.222398774	0.293179945	-5.819	NoSig
PCDH9	0.229	6.125	1.219	0.223970367	0.294990199	-5.824	NoSig
TRIOBP	-0.114	10.058	-1.216	0.225206392	0.296326377	-5.828	NoSig
POLR3B	0.108	8.805	1.216	0.225383412	0.296326377	-5.829	NoSig
WDR60	-0.263	5.861	-1.213	0.226462136	0.297481622	-5.832	NoSig
GOSR1	0.098	9.473	1.209	0.228059643	0.299315701	-5.837	NoSig
CEPT1	-0.122	8.858	-1.199	0.231621864	0.303548679	-5.848	NoSig
DUOX1	0.218	6.652	1.199	0.231693175	0.303548679	-5.848	NoSig
PCNP	-0.143	10.944	-1.195	0.233333006	0.305427975	-5.853	NoSig
PIKFYVE	-0.195	7.932	-1.189	0.235868918	0.308475885	-5.861	NoSig
C2orf72	-0.180	7.060	-1.185	0.237231297	0.309652002	-5.865	NoSig
VIL1	-0.233	5.337	-1.185	0.23733614	0.309652002	-5.865	NoSig
ZNF160	-0.182	10.705	-1.185	0.237392927	0.309652002	-5.865	NoSig
MBD1	0.154	9.255	1.182	0.23828302	0.310540623	-5.868	NoSig
ZFC3H1	0.067	9.350	1.178	0.239978126	0.312475895	-5.873	NoSig
SMPDL3A	0.152	8.001	1.171	0.242780567	0.315848384	-5.881	NoSig
PTGS2	-0.276	6.092	-1.170	0.243443722	0.316434279	-5.883	NoSig
TAT	-0.249	6.860	-1.166	0.244848745	0.317982606	-5.887	NoSig
MACROD1	-0.181	8.328	-1.157	0.248523067	0.322472776	-5.897	NoSig
ANKRD28	-0.131	8.618	-1.152	0.250695887	0.32500853	-5.903	NoSig
MYO1D	0.303	6.177	1.138	0.25640885	0.33191112	-5.918	NoSig
FAM208B	0.136	9.845	1.138	0.256466629	0.33191112	-5.918	NoSig
PTPRM	-0.219	8.398	-1.136	0.25702741	0.332347616	-5.920	NoSig
PARVA	0.087	8.450	1.135	0.257523208	0.332699401	-5.921	NoSig
RASAL2	-0.256	6.441	-1.118	0.264740424	0.341726571	-5.940	NoSig
SIK2	-0.155	7.803	-1.104	0.270825403	0.349277862	-5.956	NoSig
NDST1	-0.122	8.332	-1.103	0.271138024	0.349378026	-5.956	NoSig
RAPGEF5	-0.110	8.462	-1.099	0.27284729	0.351276122	-5.961	NoSig
PTK6	-0.304	5.192	-1.092	0.276124629	0.355001704	-5.969	NoSig
TTLL12	-0.086	10.150	-1.092	0.276218541	0.355001704	-5.969	NoSig
SERPINE1	0.204	5.943	1.089	0.277483394	0.356159206	-5.972	NoSig
ABCD3	-0.151	9.875	-1.088	0.277598197	0.356159206	-5.972	NoSig
GOLGA7	-0.089	10.999	-1.084	0.279623689	0.358448643	-5.977	NoSig
RSRC1	-0.106	8.895	-1.083	0.279909719	0.358506246	-5.978	NoSig

RCBTB1	-0.112	7.511	-1.082	0.280600312	0.359081466	-5.979	NoSig
DPYD	0.149	7.640	1.080	0.281292337	0.359657528	-5.981	NoSig
COL1A1	0.351	11.520	1.073	0.284468712	0.363406335	-5.989	NoSig
RAG1	-0.205	5.029	-1.069	0.286276133	0.365401381	-5.993	NoSig
DNAJC13	-0.151	8.800	-1.068	0.286750292	0.365692697	-5.994	NoSig
MFAP3	0.123	8.431	1.066	0.287770561	0.366679369	-5.996	NoSig
INPP5B	0.181	6.879	1.063	0.288976623	0.367900889	-5.999	NoSig
SYNCRIP	-0.100	11.540	-1.058	0.291144195	0.370251801	-6.004	NoSig
NID1	0.127	7.675	1.058	0.291321188	0.370251801	-6.004	NoSig
TBX5	0.189	6.911	1.056	0.291971346	0.370573082	-6.006	NoSig
CAMTA1	-0.339	5.674	-1.056	0.292072395	0.370573082	-6.006	NoSig
C16orf62	0.114	7.944	1.044	0.297552094	0.376887683	-6.018	NoSig
PAAF1	-0.193	7.858	-1.044	0.297556247	0.376887683	-6.018	NoSig
IL11RA	-0.127	7.621	-1.042	0.298725596	0.378046775	-6.021	NoSig
DSG2	-0.194	10.797	-1.038	0.300587688	0.38007984	-6.025	NoSig
ITGB1	-0.131	12.693	-1.037	0.300847985	0.380085772	-6.026	NoSig
EDN1	0.250	7.281	1.034	0.30212489	0.381374967	-6.028	NoSig
PHKA2	-0.136	8.498	-1.032	0.30327138	0.382497491	-6.031	NoSig
FGF4	0.233	8.047	1.024	0.307112897	0.387014303	-6.039	NoSig
CCND3	0.109	9.981	1.023	0.307610666	0.387313345	-6.040	NoSig
RGS13	-0.245	5.501	-1.014	0.311729281	0.39216704	-6.049	NoSig
LLGL2	-0.101	8.857	-1.010	0.313807776	0.394448151	-6.053	NoSig
FANCC	0.111	9.074	1.006	0.315431592	0.396154373	-6.057	NoSig
SLC39A6	0.146	11.131	1.005	0.316144106	0.396714165	-6.058	NoSig
C11orf63	0.166	6.153	1.002	0.317642955	0.398258915	-6.061	NoSig
ABCB9	0.194	6.370	0.998	0.31918757	0.399858396	-6.064	NoSig
RNF216	0.089	8.468	0.997	0.320084889	0.400644975	-6.066	NoSig
FARP2	-0.110	7.798	-0.996	0.320430171	0.400739836	-6.067	NoSig
FBXL14	0.119	8.717	0.993	0.321569108	0.401826272	-6.069	NoSig
TAF2	0.107	9.922	0.992	0.322475124	0.402620075	-6.071	NoSig
SETMAR	-0.139	7.765	-0.984	0.326204895	0.406794484	-6.078	NoSig
NADSYN1	-0.194	7.150	-0.983	0.326537703	0.406794484	-6.079	NoSig
TERT	-0.157	5.569	-0.983	0.326639283	0.406794484	-6.079	NoSig
VHL	0.314	6.175	0.981	0.327799384	0.407897643	-6.081	NoSig
CD96	0.139	6.886	0.980	0.328285064	0.408160443	-6.082	NoSig
BAGE	-0.206	5.857	-0.977	0.329841692	0.409457214	-6.085	NoSig
GTPBP3	0.089	9.653	0.977	0.329878777	0.409457214	-6.085	NoSig
TET3	-0.137	6.941	-0.975	0.330426177	0.4097946	-6.086	NoSig
RIMS1	-0.200	6.192	-0.972	0.331974394	0.411371603	-6.089	NoSig
SMEK2	0.131	6.385	0.967	0.334475632	0.413975202	-6.094	NoSig
EXD3	0.127	7.857	0.966	0.334884921	0.413975202	-6.095	NoSig
KLHL29	-0.211	5.140	-0.966	0.334910671	0.413975202	-6.095	NoSig
ITGBL1	-0.157	7.733	-0.961	0.337538373	0.416876712	-6.100	NoSig
PARK2	-0.164	6.444	-0.958	0.339153615	0.418524005	-6.103	NoSig
MFI2	-0.202	6.007	-0.957	0.339820193	0.418998861	-6.104	NoSig

POSTN	0.284	10.900	0.954	0.341172256	0.420317435	-6.107	NoSig
MYLK3	-0.186	4.910	-0.949	0.343898321	0.423325168	-6.112	NoSig
GAMT	-0.230	5.642	-0.937	0.349724118	0.430140417	-6.122	NoSig
ZNF428	-0.201	5.515	-0.936	0.35015936	0.430319809	-6.123	NoSig
RHEB	0.099	12.090	0.934	0.351090759	0.431108141	-6.125	NoSig
YIF1B	-0.085	7.803	-0.930	0.35356805	0.433791824	-6.129	NoSig
WFDC8	-0.138	6.397	-0.928	0.354569354	0.43466169	-6.131	NoSig
TMEM53	-0.097	8.178	-0.924	0.356477585	0.436640996	-6.134	NoSig
SDC3	-0.165	8.324	-0.923	0.357054385	0.436987548	-6.135	NoSig
TAL1	0.149	6.864	0.917	0.360111399	0.440366489	-6.141	NoSig
DBF4B	0.141	6.663	0.914	0.361918623	0.442212812	-6.144	NoSig
MOSPD1	-0.076	8.952	-0.910	0.363625434	0.443933514	-6.147	NoSig
KLK2	0.149	5.925	0.909	0.364186905	0.444254248	-6.148	NoSig
CBFA2T2	0.111	8.428	0.904	0.36699333	0.447310723	-6.152	NoSig
DMWD	-0.085	10.018	-0.901	0.368314521	0.448553392	-6.154	NoSig
FUS	0.070	10.732	0.897	0.370817548	0.451201803	-6.159	NoSig
SAE1	-0.064	10.385	-0.896	0.371096036	0.451201803	-6.159	NoSig
SSBP2	-0.092	7.956	-0.891	0.374054628	0.454427477	-6.164	NoSig
PGC	0.170	7.362	0.889	0.374766241	0.454799375	-6.165	NoSig
S100B	0.271	5.565	0.889	0.37516395	0.454799375	-6.166	NoSig
UBE2W	0.072	8.237	0.888	0.3752783	0.454799375	-6.166	NoSig
PACSIN2	-0.095	10.795	-0.888	0.375584524	0.454799827	-6.166	NoSig
DPY19L1	-0.140	7.600	-0.885	0.376867775	0.45598241	-6.168	NoSig
ZBTB38	-0.102	9.537	-0.884	0.377672278	0.456584291	-6.170	NoSig
HNMT	0.131	8.630	0.883	0.378257814	0.456763788	-6.171	NoSig
FILIP1L	-0.147	8.369	-0.883	0.378435095	0.456763788	-6.171	NoSig
KLHDC4	0.100	8.357	0.882	0.378817754	0.456854825	-6.172	NoSig
CENPI	-0.118	6.643	-0.879	0.38058107	0.458609442	-6.174	NoSig
GUCA1A	0.162	6.741	0.877	0.381257513	0.459052568	-6.176	NoSig
PIGQ	-0.085	7.534	-0.867	0.387023803	0.465345532	-6.185	NoSig
DAAM1	-0.142	8.664	-0.867	0.387109901	0.465345532	-6.185	NoSig
NR5A2	-0.147	6.356	-0.854	0.394074224	0.47333471	-6.195	NoSig
PLCE1	0.147	6.414	0.850	0.396426876	0.475776242	-6.199	NoSig
ZFHX4	-0.110	7.989	-0.849	0.396761473	0.475793799	-6.199	NoSig
FRY	0.152	7.077	0.848	0.397327615	0.47608877	-6.200	NoSig
RNF130	0.082	10.293	0.845	0.398913456	0.477604113	-6.203	NoSig
BANK1	-0.229	6.118	-0.844	0.399412343	0.477816697	-6.203	NoSig
ADAMTS5	0.109	7.861	0.838	0.40287124	0.481567149	-6.209	NoSig
RBBP6	0.069	9.693	0.836	0.404060451	0.48222607	-6.210	NoSig
NEUROD1	0.164	4.745	0.836	0.404071071	0.48222607	-6.210	NoSig
HERC6	0.153	8.580	0.834	0.405249407	0.482877966	-6.212	NoSig
MS4A3	-0.169	5.161	-0.834	0.405266779	0.482877966	-6.212	NoSig
ZMYND11	-0.087	11.388	-0.827	0.40929782	0.48728073	-6.218	NoSig
GSTO1	0.062	11.578	0.826	0.409617291	0.48728073	-6.218	NoSig
MAPK12	-0.120	7.214	-0.821	0.412640666	0.490484948	-6.223	NoSig

PTBP2	-0.095	8.241	-0.818	0.414285641	0.491686407	-6.225	NoSig
CYP17A1	-0.142	7.477	-0.818	0.414312756	0.491686407	-6.225	NoSig
SMPDL3B	-0.216	4.873	-0.809	0.419467093	0.497174211	-6.232	NoSig
LRRC16A	-0.076	8.743	-0.808	0.41970882	0.497174211	-6.233	NoSig
RNF141	-0.101	7.946	-0.808	0.41994002	0.497174211	-6.233	NoSig
KIF26B	-0.190	4.584	-0.807	0.420773097	0.497764196	-6.234	NoSig
SAMD14	0.115	6.050	0.797	0.426311033	0.503914551	-6.242	NoSig
RHOF	0.089	8.370	0.793	0.42837537	0.505952482	-6.244	NoSig
GINS2	0.190	8.198	0.792	0.429035153	0.506329581	-6.245	NoSig
TRAM2	-0.079	9.091	-0.788	0.431673704	0.50903949	-6.249	NoSig
SLC4A4	0.181	8.677	0.787	0.432118567	0.509160308	-6.249	NoSig
TFE3	0.110	8.516	0.785	0.433396407	0.510261645	-6.251	NoSig
PAX8	0.133	10.387	0.780	0.436504826	0.513514776	-6.255	NoSig
PAX1	0.152	7.180	0.778	0.437662994	0.514470254	-6.257	NoSig
PIK3IP1	0.112	8.660	0.774	0.439638497	0.516384238	-6.259	NoSig
ITPKB	0.147	8.139	0.772	0.440877501	0.517430816	-6.261	NoSig
TAF11	-0.072	9.263	-0.770	0.44199886	0.518337779	-6.262	NoSig
CNOT1	0.080	10.918	0.769	0.442693738	0.518743568	-6.263	NoSig
MCTP1	0.152	6.378	0.764	0.445646251	0.521792106	-6.267	NoSig
CYTH4	0.098	8.429	0.759	0.448657097	0.524904094	-6.271	NoSig
MYT1L	-0.141	5.586	-0.757	0.44994249	0.525994091	-6.272	NoSig
TRPV6	-0.177	5.798	-0.755	0.4510668	0.526894212	-6.274	NoSig
COQ10B	0.120	9.485	0.754	0.451624162	0.527131185	-6.274	NoSig
FAM120A	-0.068	12.147	-0.753	0.452354873	0.527569958	-6.275	NoSig
INPP5A	0.058	9.046	0.744	0.457893832	0.533611386	-6.282	NoSig
CTNND2	0.135	6.614	0.739	0.460544757	0.536280387	-6.285	NoSig
MYCBP2	0.093	9.790	0.727	0.46801946	0.544557854	-6.294	NoSig
ECE2	0.110	7.481	0.720	0.472458721	0.549236041	-6.299	NoSig
ADAM15	0.165	7.429	0.719	0.472778839	0.549236041	-6.299	NoSig
TTC12	-0.089	7.076	-0.712	0.477270429	0.554021177	-6.305	NoSig
NEK1	-0.067	8.315	-0.704	0.482127847	0.559223174	-6.310	NoSig
ZNF24	-0.051	10.512	-0.703	0.483102812	0.559917289	-6.311	NoSig
PVR	-0.055	8.613	-0.692	0.489393379	0.56676632	-6.318	NoSig
ACOT11	0.108	6.911	0.690	0.490754103	0.567899884	-6.319	NoSig
ACSM5	0.113	7.355	0.689	0.49157514	0.568145552	-6.320	NoSig
TCTN1	-0.073	8.520	-0.689	0.491730548	0.568145552	-6.321	NoSig
RGS5	0.160	7.731	0.688	0.492363534	0.568415092	-6.321	NoSig
IFT122	0.069	8.999	0.687	0.492728348	0.568415092	-6.322	NoSig
TIE1	0.180	3.447	0.683	0.495169831	0.57078879	-6.324	NoSig
TK1	0.088	9.776	0.678	0.498452874	0.574128136	-6.328	NoSig
FIP1L1	-0.109	8.111	-0.674	0.500986816	0.576600151	-6.330	NoSig
NSMAF	0.059	9.564	0.672	0.5022845	0.577210907	-6.332	NoSig
MOB3B	0.071	8.711	0.672	0.502293823	0.577210907	-6.332	NoSig
MICALL2	0.130	8.062	0.668	0.504873866	0.579725382	-6.334	NoSig
LRP1	0.072	7.700	0.667	0.505261664	0.579725382	-6.335	NoSig

CD79B	-0.130	6.527	-0.665	0.507006463	0.581094677	-6.337	NoSig
ESRRB	-0.105	3.916	-0.664	0.507236645	0.581094677	-6.337	NoSig
RBM6	-0.053	9.237	-0.659	0.510792764	0.584451189	-6.340	NoSig
FGF22	0.114	5.956	0.658	0.51095262	0.584451189	-6.341	NoSig
SLC5A12	-0.157	3.503	-0.654	0.513736445	0.587183777	-6.343	NoSig
UROS	0.064	8.765	0.651	0.515769444	0.588923463	-6.345	NoSig
C19orf66	0.084	9.439	0.650	0.516279502	0.588923463	-6.346	NoSig
GYPE	0.138	4.072	0.650	0.516446668	0.588923463	-6.346	NoSig
APH1A	-0.075	10.656	-0.646	0.518693806	0.591032712	-6.348	NoSig
C1QTNF3	0.072	7.563	0.639	0.523605509	0.596172582	-6.353	NoSig
WDR62	-0.083	7.124	-0.634	0.526511126	0.599022222	-6.356	NoSig
ZNF33B	0.076	7.936	0.624	0.533569972	0.606589104	-6.363	NoSig
CDH19	0.155	6.271	0.620	0.535957471	0.608420819	-6.365	NoSig
ZHX2	-0.091	8.796	-0.620	0.535999511	0.608420819	-6.365	NoSig
KAZALD1	-0.118	6.377	-0.610	0.542290969	0.615009439	-6.371	NoSig
SCEL	0.164	3.691	0.610	0.542631059	0.615009439	-6.371	NoSig
ADAMTS20	-0.101	5.952	-0.592	0.554380664	0.627847713	-6.381	NoSig
CNR1	-0.101	6.353	-0.589	0.556417103	0.629674454	-6.383	NoSig
BRS3	0.118	6.605	0.586	0.558552055	0.631609814	-6.385	NoSig
LILRA5	-0.089	7.160	-0.582	0.561110691	0.634020971	-6.387	NoSig
GABRA2	0.102	5.519	0.576	0.564918365	0.637838731	-6.390	NoSig
C3orf52	-0.098	6.628	-0.574	0.566433555	0.639064261	-6.392	NoSig
GNG4	0.094	8.324	0.566	0.572200715	0.644431583	-6.396	NoSig
RGR	0.123	6.436	0.565	0.57245939	0.644431583	-6.397	NoSig
NT5E	0.137	6.726	0.565	0.572491003	0.644431583	-6.397	NoSig
RBM14	-0.093	8.651	-0.564	0.573429875	0.64500017	-6.397	NoSig
SLC4A1	-0.160	6.925	-0.562	0.574399104	0.645602017	-6.398	NoSig
TUBGCP3	-0.139	7.639	-0.561	0.575205571	0.646020154	-6.399	NoSig
PTPLA	-0.126	7.541	-0.559	0.576976115	0.64751961	-6.400	NoSig
GPR182	-0.082	7.741	-0.552	0.58128702	0.65186561	-6.404	NoSig
MYL12A	0.053	11.189	0.549	0.583693748	0.654071291	-6.406	NoSig
ARVCF	-0.074	8.453	-0.546	0.585316737	0.655396076	-6.407	NoSig
ZNF787	0.110	5.780	0.544	0.587002884	0.656789532	-6.408	NoSig
TRDN	-0.122	3.184	-0.537	0.591635833	0.661475552	-6.412	NoSig
NTRK3	0.085	8.549	0.535	0.592901474	0.662392556	-6.413	NoSig
ZKSCAN1	-0.083	9.326	-0.530	0.596360979	0.665105317	-6.415	NoSig
INPP4B	0.091	6.407	0.530	0.596658305	0.665105317	-6.416	NoSig
RALGAPA1	-0.092	8.103	-0.530	0.596671481	0.665105317	-6.416	NoSig
MYT1	0.125	7.450	0.528	0.598215824	0.666327289	-6.417	NoSig
HSPA4L	-0.076	7.320	-0.516	0.606378793	0.674914121	-6.423	NoSig
FRYL	0.054	9.625	0.512	0.609282834	0.677639172	-6.425	NoSig
SF1	-0.037	10.409	-0.508	0.612167887	0.680339049	-6.427	NoSig
PRKAA1	-0.100	6.444	-0.504	0.614614341	0.682547816	-6.429	NoSig
SORBS2	0.134	8.350	0.503	0.615701101	0.683244431	-6.429	NoSig
ALOX15B	-0.093	7.475	-0.501	0.616627193	0.683761847	-6.430	NoSig

DNASE1L3	-0.056	7.032	-0.499	0.618271388	0.685074184	-6.431	NoSig
NRP2	-0.050	8.098	-0.498	0.618837957	0.685191394	-6.432	NoSig
HEATR3	0.066	7.917	0.493	0.62267878	0.688931061	-6.434	NoSig
MPHOSPH6	0.056	10.142	0.492	0.623322446	0.689130466	-6.435	NoSig
IQCA1	0.067	7.525	0.489	0.625093491	0.690575053	-6.436	NoSig
DNM2	0.056	8.242	0.488	0.626352149	0.691004213	-6.437	NoSig
ITGA1	-0.147	4.572	-0.487	0.626411351	0.691004213	-6.437	NoSig
SEMA6A	-0.041	7.921	-0.485	0.628306835	0.692444925	-6.438	NoSig
NLRP1	0.077	6.800	0.484	0.628648722	0.692444925	-6.438	NoSig
SLC22A7	-0.083	6.208	-0.481	0.630710188	0.694201369	-6.440	NoSig
IRF6	-0.057	10.533	-0.477	0.633877707	0.697171708	-6.442	NoSig
COL9A1	-0.094	4.733	-0.476	0.634548289	0.697393426	-6.442	NoSig
KLHL4	-0.072	6.654	-0.468	0.639971261	0.702354738	-6.446	NoSig
STK38	0.042	10.208	0.468	0.640007175	0.702354738	-6.446	NoSig
SLC23A2	-0.031	7.715	-0.467	0.640809482	0.702716593	-6.446	NoSig
SIK3	0.030	8.733	0.466	0.641807842	0.703292749	-6.447	NoSig
ANKFY1	-0.033	8.342	-0.464	0.643082267	0.704170346	-6.448	NoSig
PTGIR	-0.075	5.987	-0.461	0.645088088	0.705846937	-6.449	NoSig
GRM5	-0.092	5.457	-0.459	0.646770578	0.707167537	-6.450	NoSig
CDC25A	0.076	6.410	0.453	0.651351565	0.711653033	-6.453	NoSig
KDM6B	-0.076	9.638	-0.435	0.663895167	0.72482534	-6.461	NoSig
DRP2	0.091	7.456	0.433	0.665675553	0.726235912	-6.462	NoSig
CDKL3	0.082	6.465	0.428	0.668818396	0.729129733	-6.463	NoSig
ALDH3B1	0.062	7.933	0.424	0.671764467	0.731804954	-6.465	NoSig
MOG	0.079	6.683	0.419	0.675607603	0.734976765	-6.467	NoSig
SLAMF1	0.049	7.805	0.419	0.675664585	0.734976765	-6.467	NoSig
CDKN3	0.038	9.511	0.418	0.676511043	0.735359591	-6.468	NoSig
LOC100506469	-0.053	9.041	-0.414	0.679069552	0.737601478	-6.469	NoSig
BTG4	-0.071	4.207	-0.412	0.68075553	0.738893046	-6.470	NoSig
TNXB	-0.056	7.049	-0.409	0.682701582	0.740464809	-6.471	NoSig
RALGPS1	-0.057	6.325	-0.407	0.684418626	0.741786076	-6.472	NoSig
FBXW2	0.037	9.043	0.404	0.686615329	0.743624905	-6.473	NoSig
GCLC	0.044	8.776	0.402	0.688090287	0.744679954	-6.474	NoSig
CTNNA1	0.037	11.394	0.401	0.688979151	0.744679999	-6.475	NoSig
STEAP3	-0.037	9.892	-0.401	0.689091916	0.744679999	-6.475	NoSig
TACC1	-0.048	10.513	-0.400	0.689763756	0.74486471	-6.475	NoSig
NLGN4Y	-0.085	4.778	-0.395	0.693069318	0.747891202	-6.477	NoSig
CNPY3	-0.082	7.960	-0.390	0.697087404	0.751681631	-6.479	NoSig
TTLL5	-0.030	8.637	-0.380	0.704470003	0.759091953	-6.483	NoSig
SLC1A6	0.059	5.041	0.378	0.705830811	0.760007542	-6.483	NoSig
NOL9	-0.027	9.661	-0.374	0.708443433	0.76221999	-6.485	NoSig
ATP6V0A2	-0.046	7.219	-0.374	0.708910724	0.76221999	-6.485	NoSig
SERPINB6	-0.041	9.941	-0.369	0.712233099	0.765238887	-6.487	NoSig
CASP8	0.048	8.653	0.367	0.714207517	0.766266709	-6.488	NoSig
TTC26	0.062	6.468	0.367	0.714220349	0.766266709	-6.488	NoSig

TPCN1	0.030	9.352	0.362	0.717481411	0.769210424	-6.489	NoSig
EHBP1L1	-0.045	7.894	-0.361	0.718257283	0.769399735	-6.490	NoSig
MINA	-0.032	9.224	-0.361	0.718692825	0.769399735	-6.490	NoSig
GABRG3	0.056	6.102	0.358	0.720603802	0.770890542	-6.491	NoSig
PIP4K2B	-0.025	9.003	-0.353	0.724430382	0.774427016	-6.492	NoSig
CYP19A1	0.082	6.204	0.350	0.726664191	0.776256933	-6.493	NoSig
ASTN2	-0.058	6.307	-0.347	0.729075001	0.778273171	-6.495	NoSig
RGS11	0.057	5.889	0.339	0.734590101	0.783099472	-6.497	NoSig
ZBTB40	0.040	8.752	0.339	0.734649471	0.783099472	-6.497	NoSig
MYH11	-0.068	6.217	-0.335	0.737949402	0.786053553	-6.499	NoSig
PDK4	0.069	6.068	0.333	0.739348631	0.786980254	-6.499	NoSig
BLZF1	-0.065	6.366	-0.332	0.74014644	0.78726592	-6.499	NoSig
SHANK2	0.050	8.358	0.329	0.742739779	0.78945965	-6.501	NoSig
BNC1	0.062	6.472	0.326	0.744527499	0.790794565	-6.501	NoSig
GVINP1	-0.040	8.106	-0.321	0.748245274	0.794176104	-6.503	NoSig
EIF2B5	0.023	9.290	0.320	0.749552841	0.794996487	-6.503	NoSig
UBE2O	-0.063	6.645	-0.316	0.752592147	0.797651121	-6.505	NoSig
DNAJB9	-0.024	10.294	-0.312	0.755047556	0.799683559	-6.506	NoSig
CYP2A6	0.068	8.444	0.308	0.75836161	0.80262186	-6.507	NoSig
TBC1D16	-0.037	9.071	-0.302	0.762827404	0.806774075	-6.509	NoSig
SFXN3	0.029	8.190	0.296	0.767404909	0.810499552	-6.511	NoSig
ECM2	0.036	7.689	0.296	0.76744006	0.810499552	-6.511	NoSig
C14orf159	0.039	8.034	0.294	0.769172648	0.811752823	-6.511	NoSig
FOXN1	0.029	3.992	0.293	0.769737937	0.811773271	-6.511	NoSig
CEL	-0.065	4.429	-0.289	0.772929025	0.814409843	-6.513	NoSig
PIEZO2	-0.040	7.521	-0.288	0.773333355	0.814409843	-6.513	NoSig
CHD1L	-0.029	10.597	-0.285	0.775620791	0.816240705	-6.514	NoSig
KLF11	-0.026	8.666	-0.275	0.783321554	0.823761776	-6.516	NoSig
MTHFD2L	-0.048	6.234	-0.274	0.784542974	0.824463182	-6.517	NoSig
PGPEP1	-0.019	8.525	-0.270	0.787503017	0.826989397	-6.518	NoSig
STRADA	-0.031	10.005	-0.268	0.78875638	0.827535633	-6.518	NoSig
ARSB	-0.032	7.236	-0.268	0.789136199	0.827535633	-6.518	NoSig
SLC4A5	0.035	6.975	0.260	0.794737573	0.832464529	-6.520	NoSig
FUT8	0.068	7.342	0.260	0.79495604	0.832464529	-6.520	NoSig
KLHL3	0.037	7.204	0.255	0.798874901	0.835979576	-6.522	NoSig
ANO1	0.059	7.148	0.253	0.800558196	0.837151925	-6.522	NoSig
EML4	0.025	8.696	0.252	0.801328501	0.837368574	-6.523	NoSig
RPE	-0.032	7.898	-0.250	0.803113954	0.838644979	-6.523	NoSig
NCALD	-0.043	8.777	-0.249	0.803756876	0.83872735	-6.523	NoSig
ITGAX	0.053	5.969	0.246	0.805856733	0.840328866	-6.524	NoSig
TGIF1	-0.023	9.232	-0.245	0.806882186	0.840808557	-6.524	NoSig
SSH1	0.016	9.183	0.243	0.80811782	0.841506442	-6.525	NoSig
GIGYF2	-0.016	9.578	-0.242	0.808867412	0.841656613	-6.525	NoSig
DYNC2LI1	0.031	7.287	0.241	0.809394053	0.841656613	-6.525	NoSig
SYN1	-0.053	6.173	-0.238	0.812105539	0.843886049	-6.526	NoSig

TRAPPC2	0.023	8.094	0.228	0.819672093	0.851153912	-6.528	NoSig
ENTPD4	0.027	9.088	0.227	0.820492822	0.851411603	-6.528	NoSig
UGGT2	-0.034	8.079	-0.225	0.821835591	0.852210267	-6.529	NoSig
CDC42BPB	-0.052	7.264	-0.223	0.823901583	0.853645527	-6.529	NoSig
MCM3AP.AS1	-0.025	7.396	-0.222	0.824367839	0.853645527	-6.529	NoSig
TIPRL	-0.032	9.411	-0.212	0.831926044	0.86087267	-6.532	NoSig
RECQL5	0.025	7.111	0.200	0.841715879	0.870397435	-6.534	NoSig
ZNF611	-0.025	9.513	-0.196	0.844973676	0.873159038	-6.535	NoSig
TSC22D4	-0.017	7.848	-0.189	0.850429119	0.878186181	-6.536	NoSig
PHF21A	-0.029	8.159	-0.182	0.855905606	0.883228061	-6.537	NoSig
FBXO9	-0.013	11.103	-0.173	0.862826865	0.889752807	-6.539	NoSig
GATA4	-0.032	4.913	-0.168	0.866909106	0.893342925	-6.540	NoSig
CADPS	0.026	6.159	0.163	0.870369998	0.896288218	-6.541	NoSig
THSD4	0.025	6.868	0.162	0.871304948	0.896630074	-6.541	NoSig
DNAJC3	0.026	7.804	0.156	0.876193223	0.901036876	-6.542	NoSig
WFS1	-0.012	9.213	-0.154	0.87812847	0.902402927	-6.542	NoSig
GYPA	0.028	5.196	0.144	0.88584918	0.909708378	-6.544	NoSig
CEP112	0.020	6.445	0.142	0.887398756	0.910670773	-6.544	NoSig
SLC36A1	0.021	7.891	0.132	0.895019818	0.917858255	-6.545	NoSig
ITIH5	0.021	7.527	0.130	0.896665779	0.918912483	-6.545	NoSig
DNAH17	-0.019	6.885	-0.128	0.898007825	0.919654019	-6.546	NoSig
BCL2L14	0.019	8.561	0.126	0.899987754	0.921047344	-6.546	NoSig
RPS6KA2	-0.010	8.696	-0.118	0.905862525	0.926421991	-6.547	NoSig
STAU2	-0.010	8.266	-0.117	0.906619894	0.926559301	-6.547	NoSig
ZAK	-0.014	7.444	-0.116	0.907866844	0.926821338	-6.547	NoSig
LMO3	-0.022	7.889	-0.116	0.908122858	0.926821338	-6.547	NoSig
INTS6	0.016	8.430	0.114	0.9094968	0.927129478	-6.547	NoSig
ALPL	-0.016	6.869	-0.114	0.90967176	0.927129478	-6.547	NoSig
KLHL18	-0.009	9.015	-0.111	0.911422996	0.928278079	-6.548	NoSig
VPS13D	-0.007	8.738	-0.106	0.915710533	0.931434311	-6.548	NoSig
DR1	-0.009	9.644	-0.106	0.915774689	0.931434311	-6.548	NoSig
MAP3K13	-0.022	6.135	-0.099	0.921529044	0.936646404	-6.549	NoSig
SMOX	0.015	8.078	0.096	0.923560752	0.938070245	-6.549	NoSig
PRO2949	0.017	6.443	0.092	0.92688525	0.940328355	-6.550	NoSig
HTR6	-0.019	7.299	-0.092	0.927048667	0.940328355	-6.550	NoSig
USP36	-0.015	6.898	-0.090	0.928559059	0.941218351	-6.550	NoSig
FABP6	-0.017	3.507	-0.081	0.93574506	0.947856202	-6.551	NoSig
PTGFR	-0.021	4.512	-0.079	0.937085988	0.948568322	-6.551	NoSig
TERF2	0.007	9.292	0.073	0.941817571	0.952709338	-6.551	NoSig
CCDC40	0.010	7.392	0.072	0.942779441	0.95303401	-6.551	NoSig
EHD4	0.004	9.134	0.058	0.953697079	0.96341546	-6.552	NoSig
ENAH	0.007	10.634	0.056	0.955736595	0.96482031	-6.552	NoSig
CANT1	-0.006	9.540	-0.050	0.95983295	0.968298233	-6.553	NoSig
RNFT2	-0.010	6.241	-0.047	0.962365278	0.970194691	-6.553	NoSig
PPM1F	-0.004	8.946	-0.044	0.964692982	0.971882428	-6.553	NoSig

CD74	-0.006	12.643	-0.035	0.971961647	0.978542294	-6.553	NoSig
SIM2	-0.006	7.406	-0.030	0.976033112	0.98197648	-6.553	NoSig
ITFG1	0.002	9.535	0.024	0.980792302	0.986097467	-6.553	NoSig
ARMCX5	-0.003	7.907	-0.023	0.981766919	0.986410411	-6.554	NoSig
NBEAL2	0.003	8.018	0.022	0.982434228	0.98641438	-6.554	NoSig
CLDN15	-0.002	7.198	-0.013	0.989373863	0.992711832	-6.554	NoSig
ZNF446	-0.002	6.671	-0.010	0.991749425	0.994178218	-6.554	NoSig
PDSS2	0.001	7.783	0.009	0.992733583	0.994178218	-6.554	NoSig
CYP39A1	-0.002	5.844	-0.009	0.992841058	0.994178218	-6.554	NoSig
FRMD8	0.001	6.186	0.007	0.994544355	0.995213631	-6.554	NoSig
ARID5B	0.000	10.360	-0.002	0.998593909	0.998593909	-6.554	NoSig
AFF4	-1.053	7.506	-6.797	9.79E-11	1.12E-08	14.059	Down
FGD2	-1.381	7.705	-6.591	3.15E-10	2.56E-08	12.935	Down
MYO16	-1.027	4.434	-6.584	3.27E-10	2.56E-08	12.899	Down
GLS2	-1.017	6.120	-6.495	5.41E-10	3.82E-08	12.417	Down
CCDC132	-1.418	6.484	-6.487	5.65E-10	3.82E-08	12.375	Down
MORN1	-1.310	4.596	-6.381	1.02E-09	5.81E-08	11.814	Down
ARMCX4	-1.121	4.664	-6.160	3.38E-09	1.54E-07	10.660	Down
CHD2	-1.283	7.892	-6.078	5.28E-09	1.91E-07	10.235	Down
FHL5	-1.254	5.607	-6.039	6.49E-09	2.19E-07	10.037	Down
NAV3	-1.133	5.719	-6.016	7.32E-09	2.37E-07	9.922	Down
PPIL6	-1.089	4.746	-5.960	9.84E-09	2.81E-07	9.639	Down
ATP8B3	-1.168	4.788	-5.464	1.25E-07	2.02E-06	7.213	Down
TPM4	-1.257	11.394	-5.302	2.77E-07	3.64E-06	6.457	Down
DNASE1	-1.405	4.078	-5.267	3.28E-07	4.14E-06	6.295	Down
UBE2G2	-1.112	9.845	-5.162	5.42E-07	6.15E-06	5.820	Down
RAD54L2	-1.108	6.080	-5.155	5.60E-07	6.22E-06	5.788	Down
GPR37L1	-1.159	4.797	-5.155	5.60E-07	6.22E-06	5.788	Down
FLCN	-1.114	7.364	-5.108	7.01E-07	7.19E-06	5.575	Down
C7orf63	-1.201	5.686	-5.081	7.97E-07	7.95E-06	5.454	Down
RPAIN	-1.036	7.100	-4.974	1.32E-06	1.21E-05	4.977	Down
LUZP1	-1.062	8.182	-4.923	1.67E-06	1.45E-05	4.753	Down
COCH	-1.453	8.151	-4.904	1.82E-06	1.54E-05	4.672	Down
MUC6	-1.466	6.272	-4.903	1.83E-06	1.54E-05	4.667	Down
BPNT1	-1.234	6.208	-4.869	2.14E-06	1.78E-05	4.519	Down
OTUD7B	-1.245	4.454	-4.737	3.88E-06	2.94E-05	3.956	Down
EPB41	-1.502	4.948	-4.643	5.87E-06	4.20E-05	3.565	Down
MCPH1	-1.045	4.018	-4.423	1.52E-05	9.17E-05	2.668	Down
SLC12A1	-1.195	4.894	-4.358	2.01E-05	0.000115015	2.408	Down
FAM13C	-1.347	3.575	-4.345	2.12E-05	0.000120093	2.356	Down
ZBTB44	-1.269	5.710	-4.208	3.74E-05	0.00019188	1.826	Down
ZNF81	-1.057	5.954	-4.202	3.84E-05	0.000194878	1.802	Down
PDE12	-1.085	6.481	-4.106	5.66E-05	0.000271389	1.440	Down
ETV3	-1.192	4.816	-4.104	5.71E-05	0.000273027	1.431	Down
ERICH1	-1.234	5.000	-4.082	6.23E-05	0.00029209	1.350	Down

DDX51	-1.016	4.967	-3.969	9.75E-05	0.000427768	0.932	Down
AFF3	-1.080	4.198	-3.933	0.000112387	0.000480227	0.800	Down
BTBD7	-1.084	5.881	-3.880	0.000137992	0.000566835	0.610	Down
PDE5A	-1.047	5.039	-3.782	0.000200329	0.000775754	0.264	Down
MTSS1L	-1.064	4.458	-3.344	0.000969035	0.002995813	-1.186	Down
EXOSC1	-1.000	5.335	-3.290	0.001167594	0.00352173	-1.356	Down
ANKRD36BP2	-1.232	3.634	-2.979	0.003212245	0.008184387	-2.272	Down
